# Preserving conceptual design integrity: strategies for enhancing interoperability in architectural digital design workflows

**DOI:** 10.1038/s41598-024-78640-8

**Published:** 2024-12-23

**Authors:** Tamir El-Khouly, Omnia Abdelhalim

**Affiliations:** 1 The American University in Cairo, Cairo, Egypt; 2 Ain Shams University, Cairo, Egypt

**Keywords:** Building Information Modelling (BIM), Architectural design, 3D modelling, Rhinoceros, Revit, Interoperability, Construction documentation, Design integrity, Computational modelling, Engineering, Civil engineering, Information technology

## Abstract

This research addresses the persistent challenges of data loss and inefficient workflow integration between 3D digital design tools (Rhinoceros) and Building Information Modelling (BIM) platforms (Revit) in the architecture, engineering, and construction (AEC) industries. The study investigates how design integrity can be preserved during the transition between these platforms, focusing on ensuring compatibility while maintaining the original design intent. The research aims to answer the question: How can design integrity be preserved when transitioning from 3D design tools to BIM platforms while ensuring seamless interoperability? Using an inductive bottom-up approach, empirical data were collected from 35 student projects over three years at The American University in Cairo. Observations were used to classify challenges encountered in transitioning from Rhinoceros to Revit, synthesising the results into evidence-based best practices. Surveys and statistical analyses were conducted on the participants’ (students’) responses to validate the findings from the analysis and observations of their digital workflow experiences. This study fills a gap in existing literature, which often overlooks the technical integration between 3D design tools and BIM platforms during the creative design phase. The research offers practical solutions for improving workflow integration, reducing data loss, and enhancing collaborative efficiency. The findings are expected to impact both professional practice and education, offering pathways to more efficient design transitions and equipping students with skills to navigate digital design challenges in future AEC projects.

**Supplementary Information:**

The online version contains supplementary material available at 10.1038/s41598-024-78640-8.

## Introduction

In complex construction projects, data loss and ineffective information exchange among stakeholders can significantly undermine performance and design quality. Within the architectural, engineering, and construction (AEC) industries, the growing reliance on Building Information Modelling (BIM) has made interoperability between 3D digital design tools such as Rhinoceros and BIM platforms like Revit a critical factor in preserving design integrity throughout project development. However, challenges in workflow integration between these tools persist, often leading to disruptions in design fidelity and inefficient data exchange. This study addresses this gap by investigating how the core design concept can be preserved during the transition between these digital design platforms, focusing specifically on ensuring compatibility while maintaining the original design intent.

This research seeks to answer a key question: How can design integrity be preserved during the transition from 3D design tools (Rhinoceros) to BIM platforms (Revit) while ensuring compatibility across different software systems? The study analyses interoperability workflows, examining how design originality can be maintained during the conversion of Rhinoceros’ masses and NURBS’ surfaces into BIM-native elements within Revit.

Previous studies have examined BIM in various contexts, but the issue of interoperability, particularly between 3D design tools and BIM platforms, remains underexplored. For instance, Perović et al.^1^ discuss BIM in archaeological settings, such as at Pompeii, highlighting BIM’s expanding role in non-traditional applications. However, these studies overlook the preservation of design integrity during transitions between platforms. Similarly, Mandić et al.^2^, in their study on BIM for railway infrastructure, focus on technical solutions but do not address the creative design phase involving 3D digital design tools. Our research extends this discussion by focusing on the transition from conceptual design to technical documentation, a significant challenge in digital design workflows.

A systematic review by Ismail et al.^3^ reveals that BIM literature often emphasises infrastructure and interoperability standards but offers limited insights into the workflow integration of architectural design tools. Likewise, Shari et al.^4^ identify interoperability challenges as a major barrier to BIM adoption in Malaysia, yet their focus remains on policy and collaboration, rather than technical integration. In response to these limitations, our study explores how 3D models from Rhinoceros can be transitioned seamlessly into Revit, addressing the technical design process which has been inadequately explored in existing research.

Previous studies, such as Šupej et al.^5^, have highlighted the importance of workflow coordination in complex projects, but they do not address the early creative design phase involving architectural design tools. Our research fills this gap by providing practical solutions for seamless transitions from conceptual to technical stages, ensuring that essential design elements are preserved.

Furthermore, Perdana et al.^6^ explored the dimensions of interoperability in BIM, particularly focusing on data exchange standards and software compatibility. While these studies provide a strong foundation for infrastructure BIM, they do not tackle the specific challenges faced by architects when using 3D digital tools in early design stages. Our research addresses this gap by offering empirical solutions for overcoming these challenges, with a particular focus on preserving design originality throughout the process.

By focusing on both the theoretical framework and practical implications of design tool integration, this research contributes to academic discourse while providing actionable strategies that reduce data loss, minimise human error, and enhance collaborative workflows. The findings are expected to impact both practice and education. For practitioners, they offer a pathway to more efficient workflow management, enabling architects to transition between 3D design tools and BIM platforms without sacrificing the original design intent. In education, the research provides a foundation for developing teaching methodologies that equip students with the necessary skills to navigate digital design challenges. Ultimately, the insights from this study have the potential to improve the quality of architectural projects, leading to the development of more high-fidelity AEC models.

The study employs an inductive bottom-up approach, reflecting the complexity of the digital design process to conduct hypothesis-testing deductive approach of controlled experiments. Due to the multifaceted nature of interoperability issues, controlled-parameter experiments are impractical. Instead, empirical data were collected from 35 student projects over a three-year period at The American University in Cairo, during teaching the “Digital Representation Tools for Architects” course (ARCH 273/1521). The methodology involved detailed observations, classifying the challenges encountered during transitions to BIM-native elements in Revit, and grouping them into patterns based on iterative design loops between Rhinoceros and Revit. Surveys and statistical analyses were conducted on the participants’ (students’) responses to validate the findings from the analysis and observations of their digital workflow experiences. This approach ensured a robust evaluation of the data by incorporating participant feedback, thereby enhancing the reliability and accuracy of the study’s results. These findings were synthesised into evidence-based best practices, offering practitioners strategies to retain control over design concepts during the transition to BIM, thereby ensuring minimal disruption to design integrity in the production of construction documentation.

The primary objectives of this research are to: (1) understand how the original design concept developed in Rhinoceros can be preserved when transitioning to Revit; (2) explore strategies for enhancing interoperability between Rhinoceros and Revit without compromising design integrity; (3) identify and resolve challenges arising from data exchanges between different 3D design tools in architectural projects; and (4) provide practical solutions that architects and engineers can apply to communicate and exchange data with minimal alterations to the original design. These objectives aim to contribute to both professional practice and education, offering insights that improve design precision and data exchange efficiency in AEC projects.

## Literature review

Building Information Modelling significantly enhances design, planning, and construction by integrating multidimensional information models and advanced analytical tools, which foster seamless communication, collaboration, and standardized data exchange among stakeholders, as demonstrated by Costin and Eastman^7^, Perumal^8^, Becerik-Gerber et al.^9^, and Ozorhon and Karahan^10^. Unlike traditional CAD 2D systems, BIM ensures precise information transfer across project phases, minimizing time and cost inefficiencies often caused by insufficient data during design and construction, as reaffirmed by these studies.

A growing debate has emerged within the architectural community regarding the limitations of Autodesk Revit and BIM software. Several leading architecture firms have expressed dissatisfaction with Autodesk’s lack of innovation, citing that Revit, a key BIM tool, has not evolved to meet the increasing complexities of digital design. In an open letter, firms criticised Autodesk for high costs and insufficient development in interoperability and efficiency, which they claim hampers architectural creativity and productivity. The letter highlights how Revit’s inefficiencies cause delays, increase project costs, and restrict design freedom, impacting the delivery of high-quality projects.

In response, Autodesk acknowledged the concerns raised by architects but defended its development approach, asserting that Revit remains crucial in supporting BIM adoption globally. The company promised future improvements, including a focus on enhancing collaborative tools and interoperability between Revit and other design software. Despite these assurances, the architectural community remains sceptical, calling for Autodesk to prioritise user feedback and the evolving needs of the profession^11–14^.

The debate underscores a broader challenge within the industry: the need for more progressive digital tools that align with the creative and technical demands of contemporary architectural practice while maintaining flexibility in cross-platform workflows.

### The concept of interoperability across engineering domains

Interoperability, essential for the smooth exchange of data between diverse software platforms, is not limited to the architecture, engineering, and construction (AEC) industries. In fields such as mechanical engineering, aerospace, and civil infrastructure, the challenge of integrating complex 3D modelling tools with simulation software has been widely explored. For example, studies like Ozturk^15^ delve into Industry Foundation Classes (IFC) and BIM data schemas, examining their limitations and strengths in enabling seamless data exchanges across disciplines. The lessons learned from these fields can inform the architectural domain, particularly in understanding how standardisation improves interoperability and reduces data loss during cross-platform transitions. Incorporating these interdisciplinary approaches can broaden the perspective on data exchange challenges in architecture.

### Building information modelling (BIM) and interoperability in architecture

Building Information Modelling (BIM) has revolutionised project lifecycle management by enabling integrated data exchange across the design, planning, and construction phases. Studies by Costin and Eastman^7^ and Becerik-Gerber et al.^9^ highlight BIM’s ability to enhance collaboration among stakeholders, significantly improving project efficiency. However, the effective use of BIM hinges on the seamless transfer of data between different digital design platforms, which remains a significant challenge, particularly for architects transitioning from 3D digital design tools like Rhinoceros to BIM platforms like Revit.

The literature consistently identifies interoperability as a major obstacle. Ozturk^15^ notes that achieving interoperability between exchange formats is crucial for defining data interchangeability and maintaining design integrity throughout the project lifecycle. Unlike traditional 2D CAD systems, BIM supports the accurate transfer of geometric and informational data, but as Shen et al.^16^ and Vanlande et al.^17^ explain, it struggles with compatibility when integrating complex modelling software like Rhinoceros. Despite the growth of BIM in managing construction data, the field still lacks a consistent method for preserving creative design elements during this integration.

### Interoperability challenges in digital architectural design

Architects face significant obstacles when attempting to transition their designs from 3D models in Rhinoceros into BIM-native elements in Revit. Molinos^18^ presents five workflows for converting Rhinoceros to Revit, addressing technical transitions but not adequately focusing on maintaining design originality. Solutions include direct geometry imports, family imports, and plugin-based workflows, such as Rhynamo, that facilitate complex parametric modelling integration. However, as discussed by Eastman et al.^19^, data exchange between these platforms often results in loss of information or distortion of the original design. Moreover, Tommasi and Achille^20^ argue that while Rhinoceros excels in modelling complex geometries, Revit struggles to interpret these models without compromising their design integrity, necessitating workflow optimisations to retain the original concept.

**Fig. 1:**
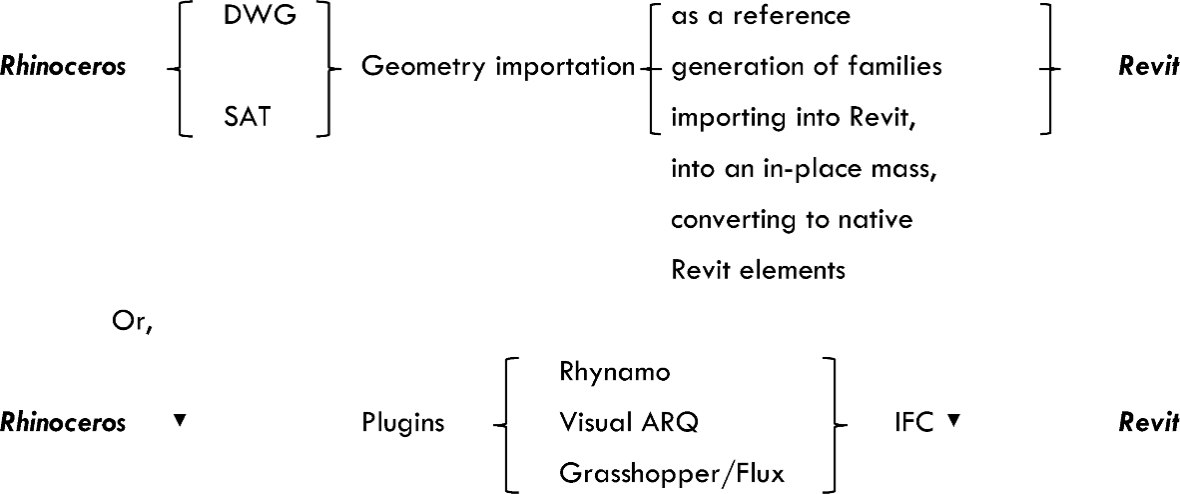
Flowchart illustrating the workflow processes for converting from Rhinoceros to Revit, based on Molinos’^18^ flowchart.

A flowchart based on Molinos^18^ illustrates the workflow processes for converting from Rhinoceros to Revit (see Fig. 1). This study aims to employ workflow techniques inspired by Molinos’ flowcharts to convert NURBS from Rhinoceros to Revit. The experimental case study tests these methods to determine the optimal approach for data exchange between the two platforms, with the long-term goal of creating comprehensive information modelling of design elements, from algorithm to technical documentation, ensuring accurate design details before construction.

Achieving interoperability between exchange formats is pivotal in defining data interchangeability, encompassing shared meaning and data exchange^21,15,16,22,17^.

### Standards for data exchange and workflow integration

Data interoperability is essential throughout a building project’s lifecycle, from design to structured drawings, as noted by Mourshed^23^. This approach is particularly valued in the AEC industry to address tool incompatibilities, as outlined by Trimble^24^.

Data exchange standards play a pivotal role in improving interoperability between 3D design tools and BIM. Ozturk^15^ discusses how standards like IFC and XML-based formats are integral to improving software compatibility across platforms. Although these standards offer a foundation for interoperability, they often fall short in maintaining the creative flexibility provided by architectural design tools. Studies like Leach^25^ and Picon^26^ delve into how parametric and algorithmic design tools have reshaped architectural workflows, yet their integration with BIM remains problematic. By focusing on developing new methods of data exchange, these studies suggest that the use of open-source plugins and proprietary interchange formats can enhance data accuracy and workflow efficiency.

Architects encounter challenges when transitioning 3D models into technical Building Information Modelling (BIM) for precise project execution and design innovation. The Industry Foundation Classes (IFC) format serves as a key solution for resolving interoperability issues in BIM projects^19^.

Consequently, the following data exchange formats are utilised:

- Direct and Private Connections between Specific BIM Tools: Direct links establish integrated connections between two applications, typically accessible through the user interfaces of one or both apps.
- Proprietary File Interchange Formats Primarily Concerned with Form Geometry: Proprietary file interchange formats are developed by engineering firms or commercial organisations to connect with their applications.
- Generic Product Data Model Interchange Formats: The IFC construction model generates product model formats containing object characteristics, materials, relationships between objects, and shape geometry.
- XML-Based Interchange Formats: XML, an extensible markup language, extends HTML, the core language of the web.

While Rhinoceros excels in precise modelling of complex surfaces and geometries, Revit stands out for modelling with information. However, it is still necessary to standardise data placement and form within a synchronous database for exchange between both modelling software types. Molinos^18^ outlines five workflows for Rhinoceros to Revit conversion:

- Direct Import: For importing geometry solely as a reference in views.
- Import-in-Mass: When imported geometry is to be presented in views such as plan views, sections, and elevations.
- Family Importation: Utilised when engineering complex families in Revit is required.
- Rhynamo: A free and open-source plugin connecting Rhinoceros to Dynamo and Revit, facilitating design of complex parametric architecture.
- Plain Data Flux: Involves managing simple information to obtain original Revit elements in large quantities, combining elements such as texts, points, and curves with Revit elements such as families or adaptive components.

### Interoperability in large-scale projects: case studies

Several case studies shed light on the practical challenges of BIM implementation in large-scale projects, particularly regarding interoperability. For instance, Becerik-Gerber et al.^9^ explore how major infrastructure projects integrate algorithmic modelling with BIM, revealing the difficulties in managing data consistency across platforms. Similarly, Perović et al.^1^, in their study of archaeological BIM projects, demonstrate how non-traditional applications of BIM encounter interoperability challenges when integrating parametric design models. These studies, while focusing on infrastructure projects, offer valuable insights that can be applied to architectural workflows, particularly in the use of complex geometries and algorithmic models.

### Gaps in the current literature

Despite the significant body of work surrounding BIM and interoperability, much of the existing research focuses on infrastructure and technical solutions, often overlooking the creative design phase in architectural projects. Studies like Ismail et al.^3^ and Shari et al.^4^ emphasise interoperability standards but do not sufficiently address the specific needs of architects when transitioning from conceptual design tools to BIM environments. The integration of 3D digital design tools like Rhinoceros with BIM remains underexplored, particularly regarding the preservation of design integrity during workflow transitions. And also, the reliance on standards like IFC has improved data exchange, but as noted in studies by Molinos^18^ and Eastman et al.^19^, more work is needed to ensure that design integrity is maintained during transitions between platforms.

In conclusion, we find that most of the research talked about two problems for architects: the loss of information as a result of communication across various systems with other departments, which is solved by BIM systems, and the challenge of converting from 3D models to technical Building Information Modelling (BIM), which was also solved by many formats to facilitate navigation between design tools and each other.

However, the researches didn’t address the challenge facing architects in maintaining the initial design of the concept during the process of converting design elements such as blocks and 3D surfaces designed in Rhinoceros to Building Information Modelling (BIM) families, including “Wall-By-Face”, “Roof-By-Face”, “Curtain System”, “Grid System” and other related families. This process enables the designer to include technical data and engineering specifications, which facilitates the production of construction drawings from the initial concept without the need for major modifications. Ensuring seamless interoperability is critical when the BIM platform rejects models generated by parametric algorithmic programs such as Rhino. This problem is the main focus of this research. By addressing these gaps and exploring cross-industry interoperability strategies, this research seeks to develop a comprehensive framework for integrating 3D design tools with BIM platforms like Revit, offering solutions to ensure the preservation of design originality in complex architectural projects.

## Research methods and methodology

This study adopts an inductive bottom-up approach, which is well-suited for addressing the exploratory nature of the research question: How can design integrity be preserved during the transition from Rhinoceros to Revit? The research centres on collecting empirical data from architectural student projects, identifying patterns, and deriving insights to propose solutions for improving workflow interoperability between these platforms.

### Sample and context

The study involved 35 student projects over a three-year period at The American University in Cairo during the ARCH 273/1521 “Digital Representation Tools for Architects” course. Students developed design concepts in Rhinoceros and transitioned these models into Revit to prepare for assigning technical specifications necessary for producing construction documentation. This academic environment provided a controlled yet relevant context, ensuring that the findings reflect real-world interoperability challenges faced in transitioning between 3D digital design tools and BIM platforms.

### Data collection

Observations were conducted throughout the design process, tracking students as they navigated the transition from Rhinoceros to Revit. Data were collected on workflow issues such as difficulties in converting NURBS surfaces into Revit-native elements like walls, roofs, and curtain systems. Additional data included error messages, workflow inefficiencies, and design alterations that occurred during the transition.

In addition to observations, students were surveyed to capture their subjective experiences. The questionnaires focused on the challenges encountered, the effectiveness of the tools, and their perceptions of the workflow. This data supplemented the observational findings, providing a comprehensive picture of the interoperability issues.

### Data analysis

The collected data were analysed using thematic coding to identify key challenges and patterns. The analysis was conducted in two phases:

1. *Pre-interoperability assessment*: Identified initial challenges in Rhinoceros, focusing on how the design was prepared for the transition to Revit.
2. *Post-interoperability assessment*: This phase assesses the effectiveness of converting designs from Rhinoceros to Revit, with a focus on evaluating how well the core design concept is preserved during the transition. Particular attention is given to the accuracy of BIM element conversions, such as walls, roofs, and curtain systems, and how these affect overall workflow efficiency. The evaluation considers whether the integrity of the original design is maintained and examines the impact of any deviations on both the design process and the production of technical documentation.

The coding process identified recurring patterns, which were synthesised into evidence-based best practices aimed at ensuring smooth transitions between Rhinoceros and Revit while preserving the original design intent.

### Role of questionnaires in validation

Questionnaires played a crucial role in ensuring the validation of the results. The surveys provided insights into the students’ personal experiences, adding a layer of subjective evaluation that complemented the objective data from observations. This use of student feedback allowed for triangulation—the combination of multiple data sources (observations, student feedback, and statistical analysis)—which enhanced the reliability and robustness of the findings. The triangulation process strengthened the validity of the study by cross-checking observed patterns with the self-reported data from participants, ensuring that the identified solutions aligned with both practical outcomes and user experiences.

Statistical analysis of student responses was employed to further validate the research outcomes, ensuring that the findings were firmly anchored in user experience. This approach provided quantitative support for the qualitative observations, reinforcing the reliability of the conclusions by directly reflecting the participants’ practical challenges and insights during the workflow transition process.

### Validation and reliability

To further ensure validity and scientific rigor, the study employed several techniques:

- *Triangulation*: Data from observations were cross-validated with student surveys, allowing for a comprehensive view of the workflow challenges. This multi-source approach helped ensure that findings were consistent and grounded in real experiences.
- *Peer debriefing*: A group of architectural professionals reviewed the findings, providing an external perspective and ensuring that the solutions proposed could be applied to professional practice.
- *Iterative analysis*: The data were revisited and analysed across different phases of the study to ensure that the conclusions drawn were reliable and repeatable.

**Fig. 2:**
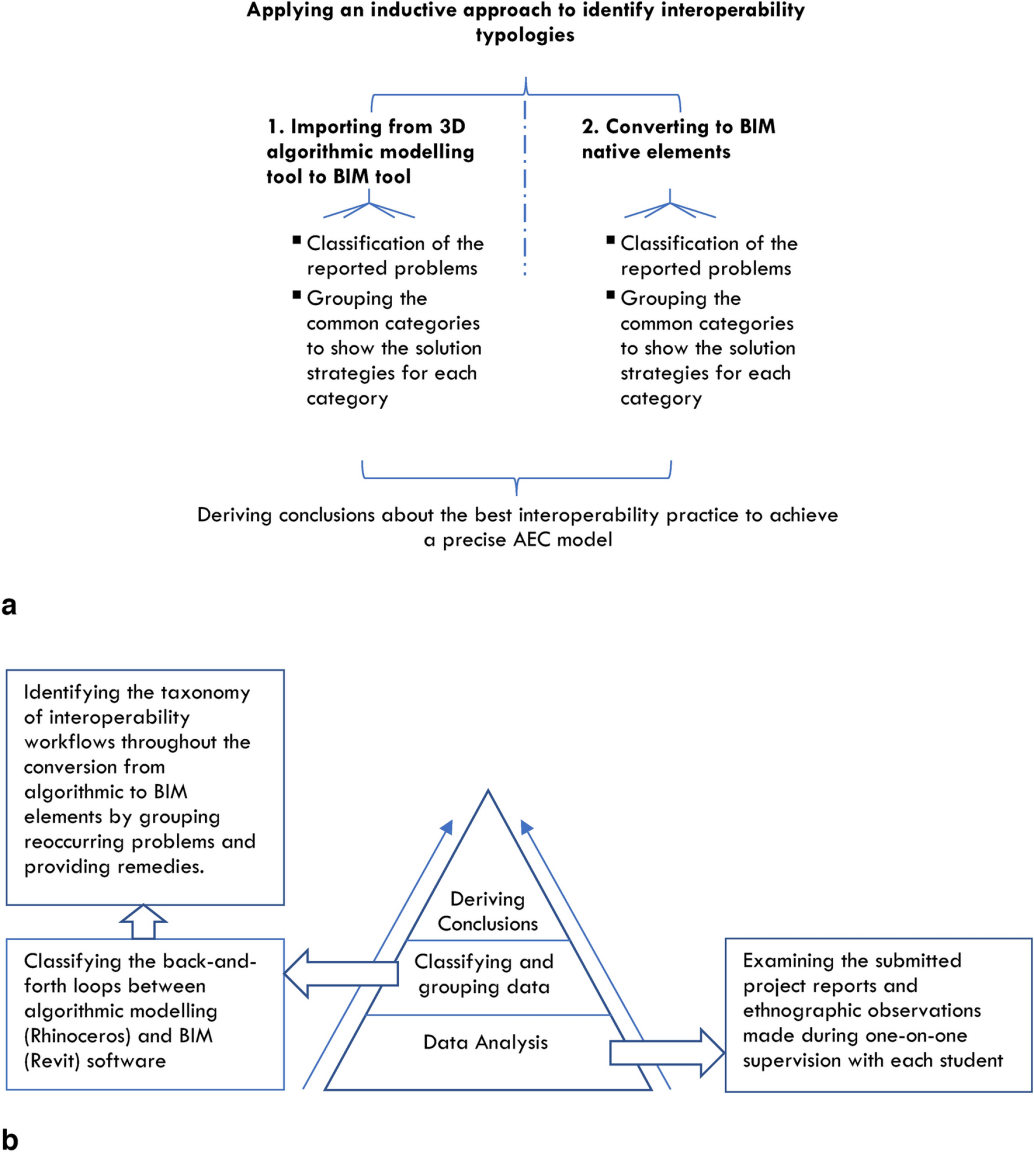
**a** Framework for research work applying a bottom-up inductive methodology. Source: authors. **b** Research framework for investigating the research problem, adopting an inductive methodology. Source: authors.

In conclusion, this methodology combines observations, thematic coding, and triangulation through student surveys and peer debriefing, ensuring a rigorous approach to analysing interoperability challenges between Rhinoceros and Revit. By validating the findings with student feedback and cross-referencing through triangulation, the study offers reliable and practical insights for improving workflow efficiency and preserving design integrity across architectural digital tools. Figures 2.a and 2.b present a framework illustrating the research process, employing a bottom-up inductive methodology to address the research problem.

### Levels of inductive methodology

The bottom-up inductive methodology used in this study comprises several key levels aimed at systematically evaluating the challenges and solutions in workflow interoperability between 3D digital design tools and BIM platforms.

- Level 1: Data Acquisition Before and After Interoperability Implementation:
  This level focuses on collecting data both prior to and following the introduction of interoperability between Rhinoceros and Revit. A comparative analysis is then conducted to evaluate the extent of changes, adjustments, or revisions in the design and composition of three-dimensional models. The analysis assesses the transition of the original design concept into native BIM elements, identifying the impact of interoperability on maintaining design integrity. This phase captures the pre- and post-interoperability stages, highlighting improvements or setbacks in the conversion process.

Example:

**Table:** 

Phase 1: Before applying conversion to Revit –Rhinoceros modelling only	Phase 2: After learning Revit	Observations
		With the Final Project, the student discovered that working effectively with Rhino and Revit allowed her to quickly implement and optimise her work. Furthermore, as the project progressed, she realised that while some tasks may appear to be the easiest, they may actually be the most rigorous and demanding; nonetheless, there is no doubt that problem-solving gets you through everything

- Level 2: Classification of Reported Problems, Error Messages, and Solution Strategies:
  At this stage, all problems, error messages, and solution strategies encountered during the workflow transition are documented and classified. This systematic categorisation enables the identification of recurring issues related to the integration of Rhinoceros and Revit, providing a clear map of the obstacles that affect workflow efficiency and design accuracy.

Example:

**Table:** 

Reported problem or error message after importing or converting Rhinoceros elements in Revit	Solution strategy	Categorisation
The ‘curtain wall’ command: ▪ In Revit, complicated walls are incompatible with the curtain wall command, which only works on simple walls The reported error message is: *Wall type “Curtain Wall” is incompatible with picked face. Only basic wall types are accepted* < refer to appendix 5.2 L.Y. >	Ease the shape, then convert to curtain wall by flexing the curve and reducing the amount of editing points in Rhinoceros or remodelling the shape in Revit	Conversion of solid mass or surface to curtain system. Surface precision when converting to curtain wall

- Level 3: Categorisation of Problems and Solutions:
  In this level, the previously classified problems are further grouped into distinct categories based on their nature and frequency. This comprehensive categorisation ensures that the entire range of problems and corresponding solutions is accounted for within the study sample. It allows for a structured approach to defining interoperability challenges, ensuring that each item is thoroughly investigated and addressed.

Example:

**Table:** 

		Reported problem or error message after importing or converting Rhinoceros elements in Revit	Solution strategy
Surfaces Category	Curvilinear Surfaces	Mesh geometry alone is incapable of computing mass floors, volumes, or surface area The reported error message is: *Mass contains only mesh geometry, which can’t be used to compute Mass Floors, volume, or surface area	Remodel in Revit or in Rhinoceros, choosing a different modelling technique and commands (approach)
		The U and V values for panels issue sometimes do not operate when importing and converting a surface (i.e. a Mobius strip) from Rhinoceros to Revit	Reduce the complexity of the surface by re-lofting, editing the points of the curves
		The U and V values for panels issue when importing and converting a surface (i.e. a curtain wall curvilinear surface) from Rhinoceros to Revit The reported error message is: *This family is empty and will be deleted. Do you want to continue?’	Re-loft the surface in Rhinoceros to reduce its complexity, then re-import it

This multi-level framework facilitates the development of interoperability typologies within algorithmic and BIM models, offering a robust method for identifying challenges and devising targeted solution strategies. The framework provides a clear pathway for improving workflow integration and enhancing the accuracy of design conversions between 3D digital tools and BIM environments.

### Analytical approach

The analysis focused on identifying recurring challenges in the interoperability between Rhinoceros and Revit, such as geometry misalignments and data loss, which impact workflow efficiency. It also examined the accuracy of converting designs into BIM-native elements and classified iterative design loops to ensure the core design concept was preserved during transitions between the two platforms.

- *Identification of Recurring Patterns in Rhinoceros-Revit Interoperability*:
  The first phase identified workflow patterns between Rhinoceros and Revit, aiming to uncover recurring issues in their interoperability. Key challenges such as geometry misalignments, data loss, and inaccurate conversions of complex design elements were identified. This analysis provided insights into critical touchpoints where workflow inefficiencies and data inconsistencies commonly occurred, highlighting areas for improvement to ensure smoother integration across platforms.
- *Analysis of Conversion to BIM-Native Elements*
  This phase focused on assessing the accuracy and design evolution during the Rhinoceros-to-Revit conversion. The study evaluated whether the design intent was preserved and the transition aligned with architectural requirements, particularly focusing on the integrity of BIM-native elements such as walls, roofs, and curtain systems.
- *Classification of Iterative Design Loops Between Rhinoceros and Revit*
  The final phase classified the back-and-forth adjustments required to translate architectural forms into BIM-native elements. This process involved refining the 3D model in Rhinoceros and testing its compatibility with Revit, ensuring the core design concept was maintained throughout. The classification identified areas needing revision to achieve a smooth and accurate conversion between the two software environments.

## Teaching methodology

The teaching methodology is structured around two pillars:

1. Empowering students to explore open-ended design tasks during early-stage concept development.
2. Developing technical proficiency in using digital tools like Rhinoceros and Revit, preparing students to navigate the challenges of digital design workflows.

An experimental study conducted at The American University in Cairo evaluated student workflows in the course “Digital Representation Tools for Architects” (ARCH 273/1521). The study focused on identifying digital workflows, examining design accuracy, and resolving issues in the Rhinoceros-Revit interoperability process. The course is divided into five phases covering spatial compositions, visualisation, technical BIM modelling, algorithm integration, and project finalisation, with practical applications linked to each phase.

### Transition to the technical phase

One of the key challenges students face is maintaining their design concepts when transitioning to the technical phase. The course integrates Rhinoceros, Revit, and Photoshop to simulate spatial ideation, representation, and technical documentation, with hands-on training in exporting and integrating files between platforms. This enables students to utilise the strengths of each tool at different project stages.

### Teaching objectives

The objectives of the teaching method are:

- Phase 1: Mastery of 3D composition using Rhinoceros, understanding abstract forms, and differentiating between meshes and NURBS.
- Phase 2: Proficiency in rendering and visualisation of spatial forms.
- Phase 3: Expertise in BIM workflows using Revit and understanding interoperability with Rhinoceros.

### Evaluation methods

To evaluate progress, students participate in:

- Project evaluations of Rhinoceros-to-Revit conversion workflows.
- Reports and questionnaires assessing challenges in interoperability.
- In-class feedback sessions focusing on real-time solutions to conversion issues.

**Fig. 3:**
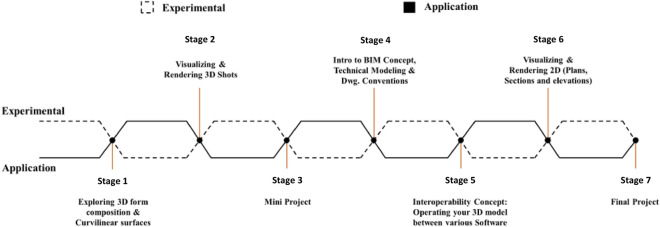
Stages in teaching ARCH 273/1521 Digital Representation Tools for Architects course at The American University in Cairo AUC. Source: authors.

As illustrated in Fig. 3, the course approach delineates the fundamental tenets of this teaching methodology.

**Fig. 4:**
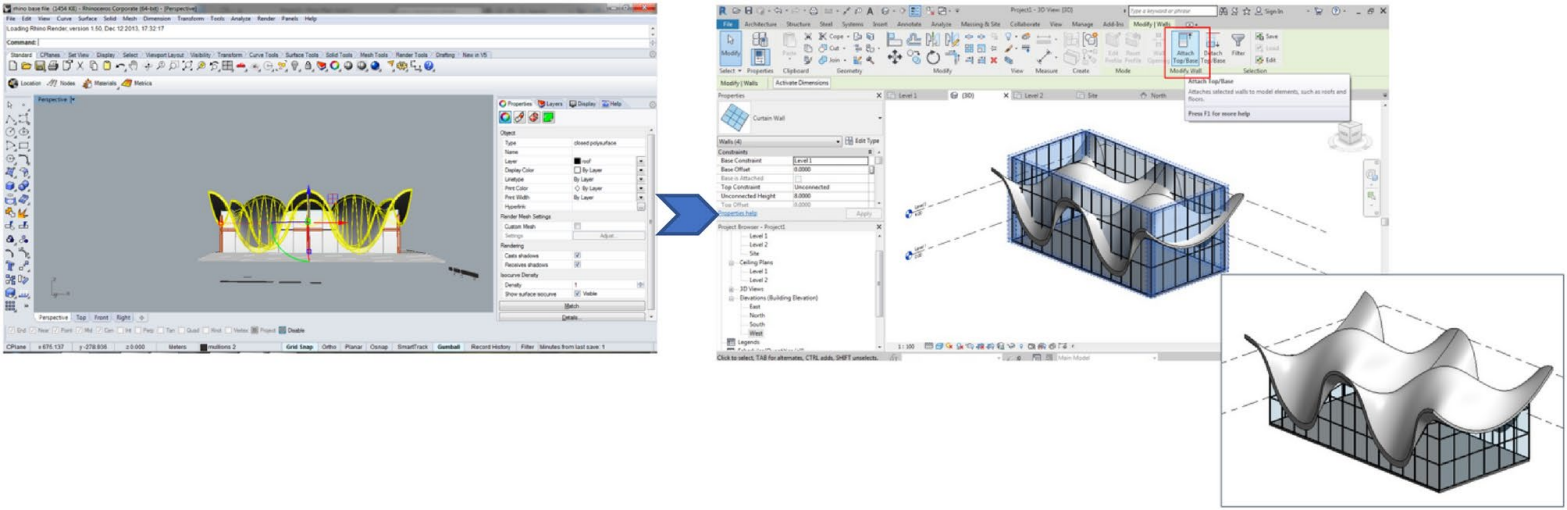
The project example of the Bosjes Chapel, designed by Coetzee Steyn and completed in 2016, sourced from ARCH 273/1521 coursework archive (Spring, 2020).

A prime example illustrating this process is the Bosjes chapel, designed by Coetzee Steyn and completed in 2016, featured in Fig. 4 and used in the teaching process.

**Fig. 5:**
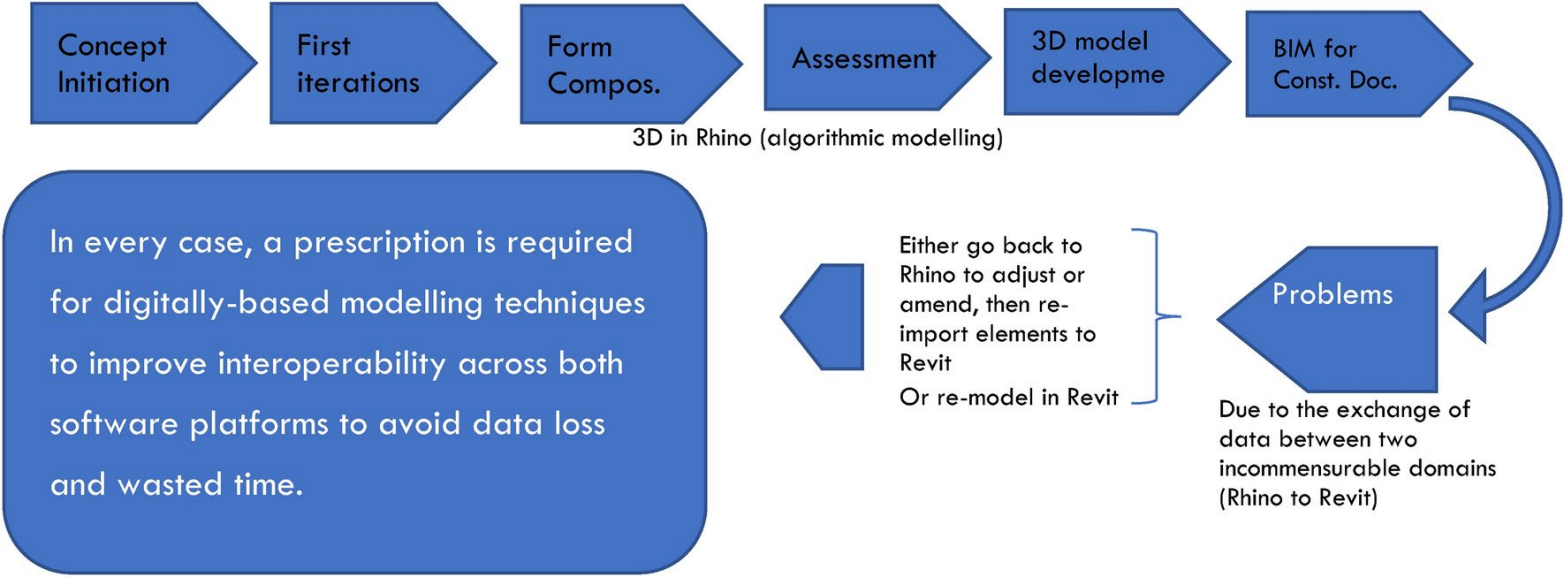
The interoperability testing process in a digital architectural design studio, ensuring that the technical goal of creating an architectural, engineering, and construction model is met. Source: authors.

The existing research predominantly focuses on construction and facilities management, with limited simulation of architectural design studios or basic ideation stages. Figure 5 provides an insight into the processes employed within a digital architectural design studio to test interoperability while conceptualising and refining project ideas, with the ultimate aim of creating comprehensive architectural, engineering, and construction models.

## Case study

The case study project provides students with an open-ended task, encouraging them to apply their Rhinoceros skills and explore various spatial composition techniques. Appendix 1 details the project briefing, outlining evaluation parameters, methodologies, and required final outputs. The case study is structured into two key phases:

### 3D design modelling without BIM implementation

**Fig. 6:**
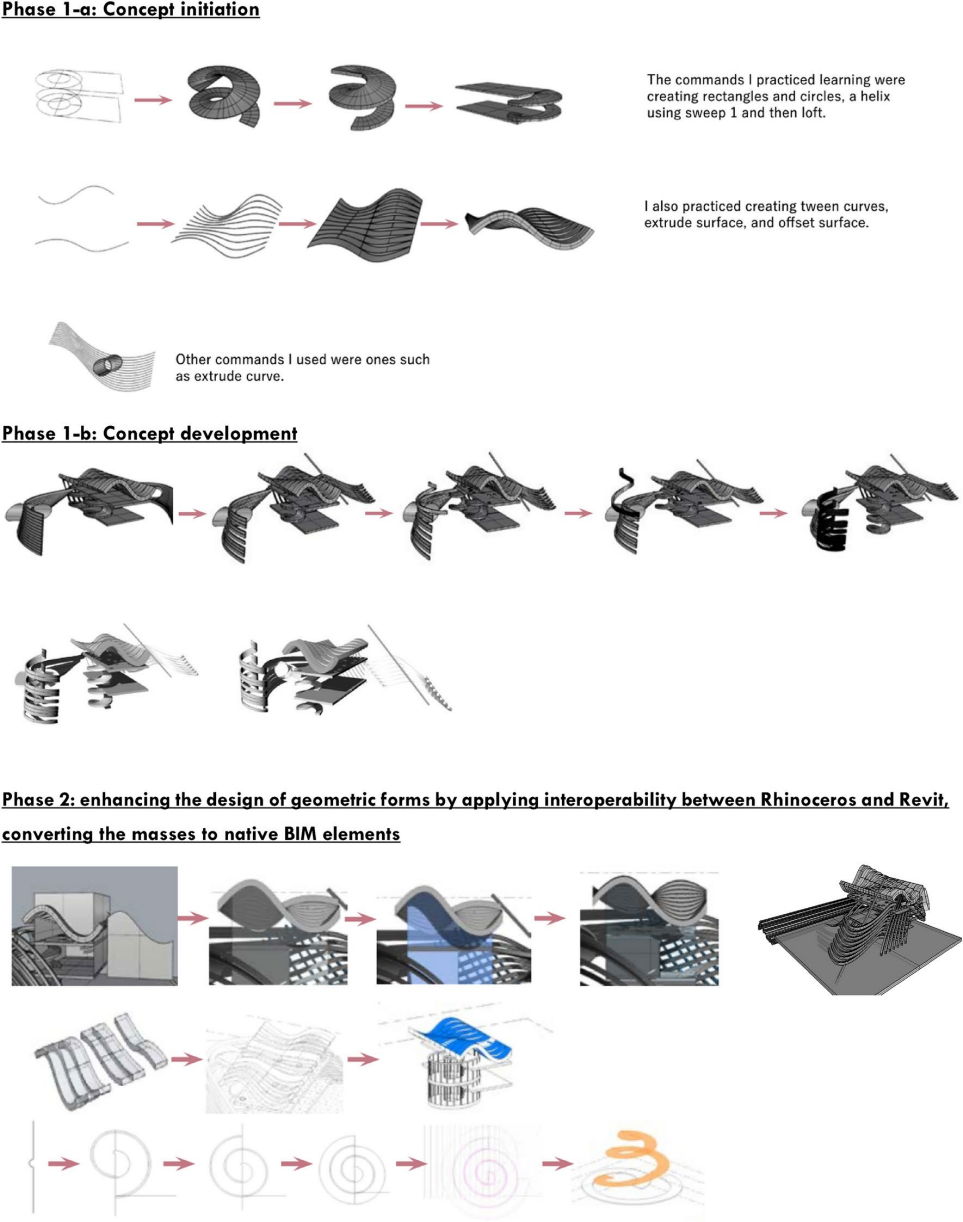
The complete process, from conceptualisation to interoperability and conversions. ARCH 273/1521 coursework archive (Spring 2020), The American University in Cairo.

In the first phase, students focus on 3D modelling without the constraints of BIM processes, allowing for unconstrained design experimentation. They are free to explore a wide range of geometric forms, including curved and rectilinear shapes, without the need to conform to technical BIM requirements. After the design phase, students begin converting their conceptual models into BIM-native components. This journey from conceptual design to interoperability is illustrated in Fig. 6, and Table 1 presents the outcomes of six sample designs from this phase, showcasing their transitions from concept to digital model.

**Table 1:** The results of six data samples developed using an algorithmic modelling tool and subsequently converted to native BIM elements, split into two sections: one in Rhino and the other after converting to Revit elements, sourced from ARCH 273/1521 coursework archive (Spring 2020–2023), The American University in Cairo.

Student Code	Phase 1: Before learning Revit –Rhino modelling only	Phase 2: After learning Revit	Observations
S1			The student successfully utilised Rhino and Revit for her Final Project, thus enabling quick implementation and optimization. Despite the challenges, she realised that problem-solving is crucial for overcoming even the most challenging tasks, highlighting the importance of effective communication and collaboration in any project
S2			The student successfully managed large files from Rhino to Revit, achieving a satisfactory end result despite the time-consuming and tiring process. The final product resembled the original Rhino model, despite some modifications and challenges
S3			Interoperability is a complex process filled with challenges. However, understanding the techniques and strategies used to solve these problems through exploration, tutorials, and critical thinking can ensure a smoother journey. Learning through trial and error is crucial to becoming skilled in using any software, as it involves learning all the techniques and methods through failure and struggle
S4			The student’s Revit model, despite taking time to construct, showcased the real-life appearance of her design, emphasising the importance of considering architectural characteristics for a functional and aesthetically pleasing model
S5			The student gained experience in interoperability through using Rhino and Revit software to convert design concepts into a 3D model in Revit, identifying them as Wall, Curtain wall, Levels, and Roof, and creating a construction drawing

### Conversion of masses and NURBS to native BIM elements

In the second phase, students engage with the complexities of converting Rhinoceros models into BIM elements such as slabs, walls, staircases, curtain walls, and grid systems. After receiving training in Revit BIM applications, they explore the challenges and solutions involved in this conversion process. While working with native BIM elements, students continue to maintain creativity within their 3D models by refining geometric shapes. Additionally, they experiment with back-and-forth workflows between Rhinoceros and Revit, enabling a dynamic process of design refinement. Table 2 highlights five examples of Rhinoceros models converted into BIM elements, illustrating the progression from algorithmic design to technical modelling.

**Table 2:** Two fundamental methods (workflows) for importing Rhinoceros masses into Revit, as per ARCH 273/1521 coursework archive (Spring 2020), The American University in Cairo.

	Workflow 1:	Direct Import	Revit masses creation:	The imported geometry from Rhinoceros is a mere reference for developing new masses in Revit
Workflows	Workflow 2:	Import-in-Mass	During the importation of masses:	• Choose units and precision based on the imported model. Divide the entire mass in Rhinoceros into individual masses (separating entities), then import each one separately • Shapes with a large number of control points and vertices cannot be imported from Rhinoceros because Revit cannot identify them, so the designer must re-model the mass in Rhinoceros using the fewest control points possible without disrupting the nature of the resulting mass • Meshes and NURBS can be used in Rhinoceros modelling before importation
			Converting a mass into native Revit elements:	This is determined by the alignment and position of the elements in the Rhinoceros mass, for example: • A floor in the imported Rhinoceros model must be a closed extrusion in order to be converted to floor-by-face in Revit • A wall in the imported Rhinoceros model must be vertical or inclined in order to be converted to wall-by-face in Revit, but it will not be changed if it has been produced in a horizontal plane • A ceiling in the imported Rhinoceros model must be horizontal or inclined in order to be converted to floor-by-mass in Revit, but it will not be changed if it has been produced in a vertical or inclined plane
			Irregular curvature masses:	Can be converted into native Revit elements using curtain systems

Throughout this phase, instructors guide students in refining their form analysis and updating their designs to ensure geometric precision within BIM constraints. Adjustments are often made in Rhinoceros, ensuring that the creativity of the original design is not compromised. The students also experiment with establishing effective bidirectional workflows between Rhinoceros and Revit, facilitating innovative solutions that enhance the evaluation of their final projects.

## Analysis

The primary aim of this project was to establish digital workflows that effectively manage the complex data transfer challenges between Rhinoceros and Revit. Additionally, it sought to enhance design processes by incorporating shop drawings, ensuring the accuracy of Architectural, Engineering, and Construction (AEC) projects while preserving creativity and conceptual design models. The study systematically categorised feedback loops from students’ reports and observations made during advisory sessions to evaluate the effectiveness of these workflows.

### Identification of processes resulting from *Rhinoceros*-revit interoperability

Involving 35 students, this phase of the study focused on uncovering the techniques students used to convert Rhinoceros masses and surfaces into Revit components. Ethnographic observations and project reports were thoroughly analysed to document interoperability challenges, difficulties encountered, and the problem-solving strategies applied. By studying these interactions, the project identified feedback loops between the two software platforms and established a taxonomy of interoperability procedures to streamline the conversion from algorithmic design to BIM-native elements.

The analysis revealed recurring challenges in the process of converting conceptual designs into accurate BIM models for AEC purposes. Issues such as geometry misalignments, data loss, and inconsistent transformations were frequent. Tables 2 and 3 provide detailed insights into these challenges and the specific procedures adopted to address them during the conversion from Rhinoceros to Revit. Appendix 2 further outlines the specific commands and tools used to convert 3D algorithmic elements into Revit-native components, offering practical guidance for navigating the complex interoperability process. These findings contribute valuable solutions for overcoming workflow inefficiencies and ensuring seamless design transitions between digital design platforms.

**Table 3:** Challenges and difficulties discovered during the Rhinoceros and Revit interoperability process for converting a conceptual design into BIM for one project, as per ARCH 273/1521 coursework archive (Spring 2022), The American University in Cairo.

Student Name Project Name	Problem	Solution	Classification
K.M (Marsprecious) Appendix 5.3	When the student inserted a mass from Rhino to identify it as curtain walls, she had to create a curtain system. Revit warned her that the panels in the curtain system might be malformed	The student determined that she had to decrease the spacing between the curtain wall grids to obtain a smoother surface, which adjusted the curtain grids on the dome.	Curtain wall
	The student built mullions that fit the same structure and shape. She tried changing the width on both sides in the dimensions panel, but it seemed as though it would only change the dimension on one side of the mullions	The student thought of changing it on one side only, then duplicating it on the other side to obtain the correct mullion size.	Curtain wall
	As the student was trying to identify the horizontal walls as mullions, they affected the vertical mullions due to the connection	Identify the mass as Roof by Face.	Roof
	The last step in the interoperability process of extracting from Rhino to Revit was the surfaces between the U and V panels. The student was receiving this message.	Re-loft the surface on Rhino to reduce the complexity of the surface, and re-import it into Revit.	Surface
	While creating the curtain walls, the student requested Revit to create a Curtain System. However, the spacing between the curtain grids was incorrect, leading to random glass panels.	Decrease the spacing between the curtain wall grids to obtain a smoother surface.	Curtain wall

### Optimization levels for importing *Rhinoceros* 3D model to revit

**Fig. 7:**
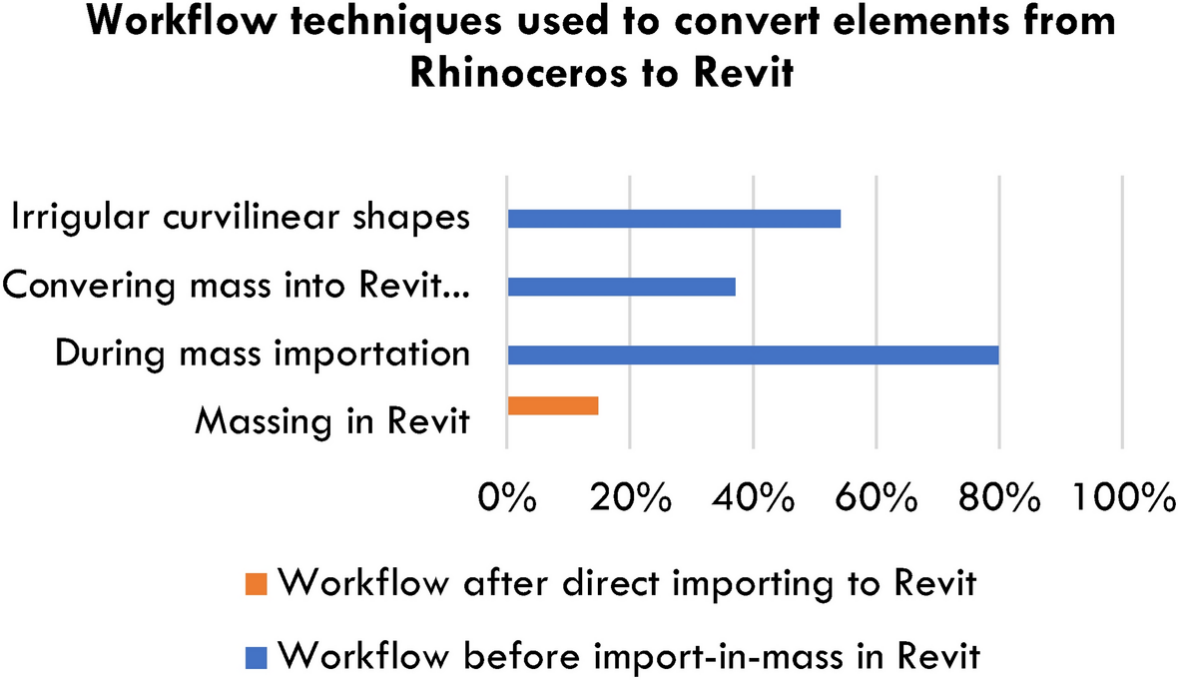
Patterns that appear repeatedly in interoperability workflows. Source: ARCH 273/1521 coursework archive (Spring 2020), The American University in Cairo.

In Revit, designers must verify the geometry of shapes for accurate curves and surface importation from Rhinoceros. During this process, the students encountered import issues while converting masses and surfaces into BIM parts to enhance design process efficiency and accuracy. The results from thirty-five student experiments are summarised in Fig. 7:

- 14.3% (5 students) directly imported without conversion.
- 85.7% (30 students) utilised the ‘import in Mass’ method.
- 79.9% (28 students) faced issues during mass importing.
- 37.1% (13 students) converted masses into native Revit elements.
- 54.2% (19 students) modelled irregular shapes in Rhinoceros, later transformed into Revit curtain systems. Some students alternated between these workflows.

Figure 7 shows recurring patterns in interoperability workflows sourced from ARCH 273/1521 coursework archive (Spring 2020), The American University in Cairo.

**Fig. 8:**
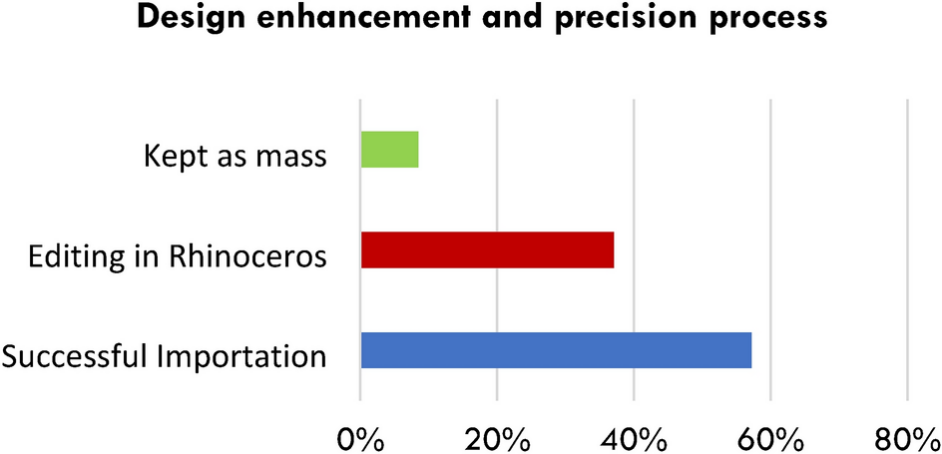
Enhancement of design and precision in converting to BIM native elements. Source: ARCH 273/1521 coursework archive (Spring 2020), The American University in Cairo.

Similarly, Fig. 8 presents outcomes from 35 students:

- 57.2% (21 students) successfully imported 3D models from Rhinoceros to Revit, then adapted them to native BIM settings.
- 37.1% (13 students) modified Rhinoceros models to match native Revit elements.
- 8.5% (3 students) used geometric masses as a reference without evaluating the overall conversion method.

Figure 8 shows design enhancement and precision in BIM element conversion.

### Characterization of back-and-forth loops during interoperability process between *Rhinoceros* and revit

Students were not initially exposed to BIM processes while modelling in Rhinoceros. The conversion attempts employed linear methods, evolving into complex strategies such as transforming surfaces into curtain or grid systems to achieve BIM compatibility. This cyclical process aligns with Tommasi’s (2017) view of BIM interoperability as a closed loop between design and transformation until the engineering objectives are met.

**Fig. 9:**
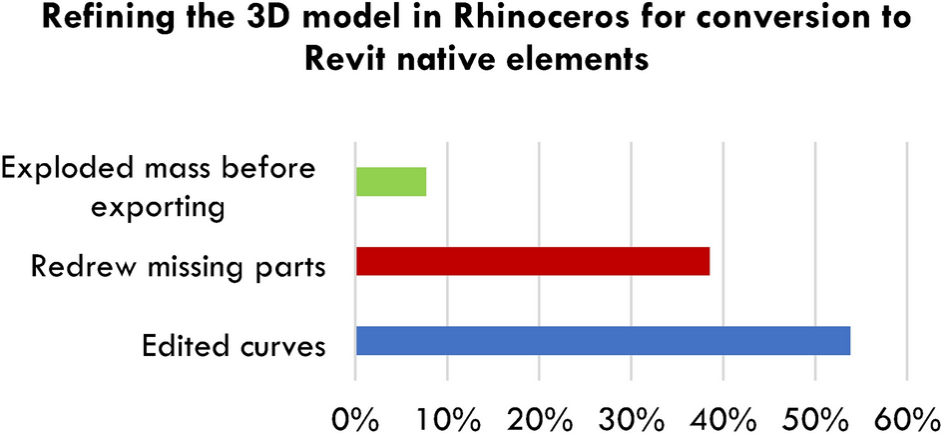
Modification categories for preparing the original design model for conversion to a BIM model. ARCH 273/1521 coursework archive (Spring 2020), The American University in Cairo.

Students were directed to maintain design integrity during BIM conversion, categorised into three transformation methods, as shown in Fig. 9:

1. Reducing control points in Rhinoceros curves to simplify conversion.
2. Rectifying open surfaces by recreating missing areas.
3. Exploding masses in Rhinoceros to control individual components before import.

Figure 9 presents the modification categories for preparing designs for BIM conversion, sourced from ARCH 273/1521 coursework archive (Spring 2020), The American University in Cairo.

The study suggests Rhinoceros modelling strategies to streamline conversion to native Revit elements, emphasising:

1. Curve precision by minimising control points.
2. Consistent surface orientation for accurate modelling.
3. Optimising surface types before exportation.
4. Ensuring edge and vertex alignment for seamless surface connections.

## Results and discussion

This section investigates the students’ utilisation of interoperability techniques to transform algorithmic surfaces and shapes into BIM elements for architectural engineering models and documentation. It also evaluates the efficacy of the teaching process by means of a questionnaire covering all the stages in the project.

### Applying an inductive approach for interoperability classification

Utilising an inductive approach, this study classifies interoperability processes into distinct groups of challenges and solutions. Table 4 illustrates the multi-level classification of obstacles observed ethnographically by instructors or reported by students in their research papers. The table, generated from a preliminary classification phase (Appendix 3), identifies scenarios involving digital design processes and interoperability within case studies.

**Table 4:** Classification of Rhinoceros surface treatment and the challenges involved in conversion to Revit architectural elements. Source: Authors.

		Reported Challenges	Solution Strategies
Technical Issues	Visibility and Scaling	Visibility and Scaling issues < refer to appendix 5.1 – N.A. >	Adjust in Revit Settings
		When exporting views on an A1-sized sheet in Revit, the scale of the building is excessively large < refer to appendix 5.4 – R.Z. >	Before exporting, scale the project in Rhinoceros
		After importing it into Revit, it is not possible to observe the mass composition < refer to appendix 5.7 – M.A. >	Rescale the model in Rhinoceros before importing it into Revit
		After importing it into Revit, it is not possible to observe the mass composition < refer to appendix 5.8 – M.S. >	Before importing the model to Revit, rescale it in Rhinoceros
		It is not possible to see the mass composition after importing it into Revit < refer to appendix 5.11 – H.A. >	Rescale the model in Rhinoceros before importing to Revit
	Importing	It is not possible to import the mass composition from Rhino into Revit all at once The reported error message is: *None of the created elements are visible in Floor Plan: Level 1 View. You may want to check the active view, its Parameters, and Visibility settings, as well as any Plan Regions and their settings < refer to appendix 5.1 N.A. >	Divide the configuration into groups of elements in Rhino before importing each one and transforming them individually into Revit elements
		It is not possible to import the mass composition from Rhino into Revit all at once < refer to appendix 5.9 – M.E. >	Divide the configuration into groups of elements in Rhino before importing each one and transforming them individually into Revit elements
		It is not possible to import the mass composition from Rhino into Revit all at once < refer to appendix 5.10 – R.E.S. >	Divide the configuration into groups of elements in Rhino before importing each one and transforming them individually into Revit elements
Surfaces Issues	Curvilinear Surfaces	Mesh geometry alone is incapable of computing mass floors, volumes, or surface area The reported error message is: *Mass contains only mesh geometry, which can’t be used to compute Mass Floors, volume, or surface area < refer to appendix 5.1 N.A. >	Remodel in Revit or in Rhinoceros, choosing a different modelling technique and commands (approach)
		Surface conversion is complicated < refer to appendix 5.1 N.A. >	Change the shape, making it less curvy or less complex, then reduce the amount of editing points to be accepted as ‘roof-by-face’ in Revit
		Curvilinear solid mass is complicated. < refer to appendix 5.1 N.A. >	Recreate solid curved surfaces by using the ‘pick line’ tool in roof or floor in Revit
		The U and V values for panels issue when importing and converting a surface (i.e. a Mobius strip) from Rhinoceros to Revit sometimes do not operate. < refer to appendix 5.3 K.M. >	Reduce the complexity of the surface by re-lofting, editing the points of the curves
		The U and V values for panels issue when importing and converting a surface (i.e. a curtain wall curvilinear surface) from Rhinoceros to Revit. The reported error message is: *This family is empty and will be deleted. Do you want to continue?’ < refer to appendix 5.3 K.M. >	Re-loft the surface in Rhinoceros to reduce its complexity, then re-import it
		A circular construction cannot be converted into native Revit BIM elements. < refer to appendix 5.4 – R.Z. >	Create floors at the widest diameter level, then draw floors with the “pick-line” tool
		The mass composition from Rhinoceros cannot be imported into Revit all at once. The curvilinear surface, resembling a roof, which was created in Rhinoceros, could not be transformed into a wall or a roof-by-face in Revit. < refer to appendix 5.6 – H.R. >	Import the roof separately and convert it into a curtain system
		The appearance of curvilinear wall components is unclear in Revit. < refer to appendix 5.6 – H.R. >	Import the wall separately and convert it back to wall-by-face
		Surface conversion is difficult. < refer to appendix 5.8 – M.S. >	Change the design, making it less curved or intricate, then reduce the number of editing points required for it to be accepted as ‘roof-by-face’ in Revit
	SUB-D	The Sub-D model could not be imported in Revit, nor converted to its BIM families. < refer to appendix 5.4 – R.Z. >	Rebuild the model in Rhinoceros from the ground up, utilizing new curves extracted from the previous sub-d version and new surfaces
		The rebuild command could not be used to reduce the number of control points on a SUB-D mesh surface. < refer to appendix 5.5 – A.A. >	Use the NURBS tool to convert objects, followed by the explode command to divide the mesh into different sections
		Could not import the Sub-D model. < refer to appendix 5.12 – O.A. >	Use the (to NURBS) command that converted the model from a SUB-D object to a NURBS object, then explode the model into small parts
	Sphere	Modelling ellipsoid-like curvilinear surfaces is problematic in Revit. < refer to appendix 5.4 – R.Z. >	Patch curves with Rhinoceros, then export each shape to Revit Export each half of a sphere separately into Revit
		Sphere-like curvilinear surfaces: the typical wall command extends beyond outside walls of the model (beyond the surface). < refer to appendix 5.4 – R.Z. >	Using the ‘edit profile’ command, adjust each wall, then inspect the component in the model from various angles to rectify any incorrect alignments
		Sphere wireframe and inner frame imported from Rhinoceros to Revit: there was no issue with the sphere’s inner and outer wireframes because they both worked with wall-by-face, but there was a problem with the size of the Revit file, which exceeded two gigabytes and made the application run very slowly. < refer to appendix 5.5 – A.A. >	In Revit, use Architecture > components > model in place > wall > insert > import CAD to import it from Rhino to Revit. This significantly decreases the size of the file
	Pipe	It is not possible to convert the pipes created in Rhinoceros into native Revit BIM elements. < refer to appendix 5.6 – H.R. >	Import them as components and identify the mass as a wall
		It is not possible to convert the pipes < refer to appendix 5.10 – R.E.S. >	Import them as components and identify the mass as a Column
	Mobius Strip	Revit rejects Mobius surface conversion; Revit was not responding. < refer to appendix 5.3 K.M. >	Divide the surface into groups of surfaces (before importing to Revit), then use ‘wall-by-face’
		Horizontal shells for the Mobius curvilinear surface cannot be imported to Revit as one bulk surface. < refer to appendix 5.3 K.M. >	Segment into sets rather than exporting everything at once, then transform the surfaces in Revit to wall-by-face
	Mesh	In Revit, the mesh geometry is unreadable. < refer to appendix 5.7 – M.A. >	Remodel in Revit or Rhinoceros, using a new modelling technique and set of commands (i.e., another modelling approach)
		It is impossible to select some components as roof-by-face in Revit due to the complexity of the forms after transforming the objects from mesh to NURBS. < refer to appendix 5.5 – A.A. >	Import the objects using Architecture > components > model in place > roof > insert > CAD import
		Mesh geometry was unreadable in Revit. < refer to appendix 5.9 – M.E. >	Remodel in Revit or in Rhinoceros, choosing a different modelling technique and commands (approach)
Wall Issues	Curtain Wall	The ‘curtain wall’ command: in Revit, complicated walls are incompatible with the curtain wall command, which only works on simple walls The reported error message is: *Wall type “Curtain Wall” is incompatible with picked face. Only basic wall types are accepted < refer to appendix 5.2 – L.Y. >	Ease the shape, then convert to curtain wall. This can be done by flexing the curve and reducing the amount of editing points in Rhinoceros or remodelling the shape in Revit
		Malformed shapes after curtain wall conversion The reported error message is: *Some panels in this curtain system are slightly malformed. The problem is most likely ignorable. This problem usually occurs when a panel or parts of a panel have been divided so that they are very narrow. To better see the malformed panels, temporarily hide the mullion category and set display pf lines to “thin lines” (using the command on the view menu) < refer to appendix 5.3 – K.M. >	It is possible to convert to smoother-surface curtain walls by tightening the space between mullions
		The curtain wall grid is inaccurate, resulting in a malformed shape < refer to appendix 5.3 – K.M. >	Reduce the grid panel size to obtain a smoother surface
		Difficulty in converting a double-curvilinear surface to a curtain wall in Revit using the curtain system. The spacing between the curtain wall grid is incorrect in Revit, leading to random glass panels < refer to appendix 5.3 – K.M. >	Adjust the spacing between the curtain wall grids to obtain a smoother surface
		The curtain wall grid is inaccurate, resulting in a malformed shape < refer to appendix 5.8 – M.S. >	Reduce the grid panel size to obtain a smoother surface
	Basic Wall	In Revit, the walls could not be connected to the roof mass < refer to appendix 5.7 – M.A. >	In Rhinoceros, redraw and trim the wall before importing it into Revit
		The very thin wall could not be totally converted < refer to appendix 5.8 – M.S. >	Convert some parts as wall-by-face and others as curtain system, thus enhancing the design idea
Selection Issues	Layers	It is challenging to select specific curves while adjusting the placement of the surfaces in Rhinoceros < refer to appendix 5.4 – R.Z. >	In Rhinoceros, separate each element in a layer, then hide the unnecessary elements while editing
	Curves	Some lines must be refined when creating Revit elements such as flooring and railings, in order to use the pick line tool to generate accurate items < refer to appendix 5.5 – A.A. >	Use AutoCAD to refine these lines. To refine the precision points, use the PEDIT Command, then re-import them into Revit
Others	Openings	A door/window cannot be assigned to a double curved surface in Revit < refer to appendix 5.4 – R.Z. >	Construct a wall-by-face in a different material and with an opening in it

Entries in Table 4 with similar attributes or addressing the same elements differently, reported as challenges by students during project interoperability, are grouped into topic headings based on their resemblances. The study employs an inductive method to categorise interoperability processes into specific challenge and solution groups.

**Fig. 10:**
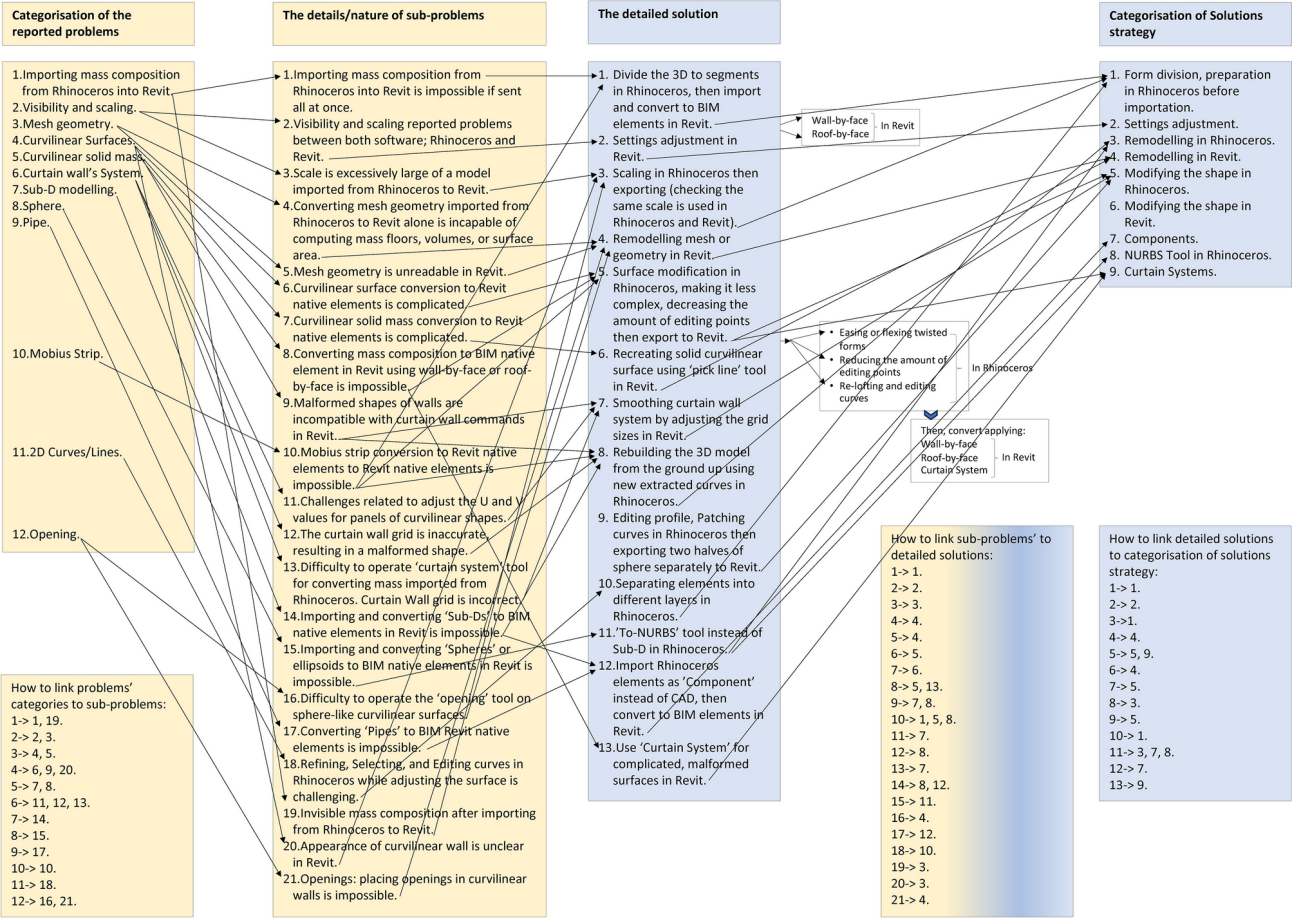
Complete flowchart of the interoperability workflow of back-and-forth linking between two different types of modelling software: Rhinoceros to Revit. Source: authors.

**Fig. 11:**
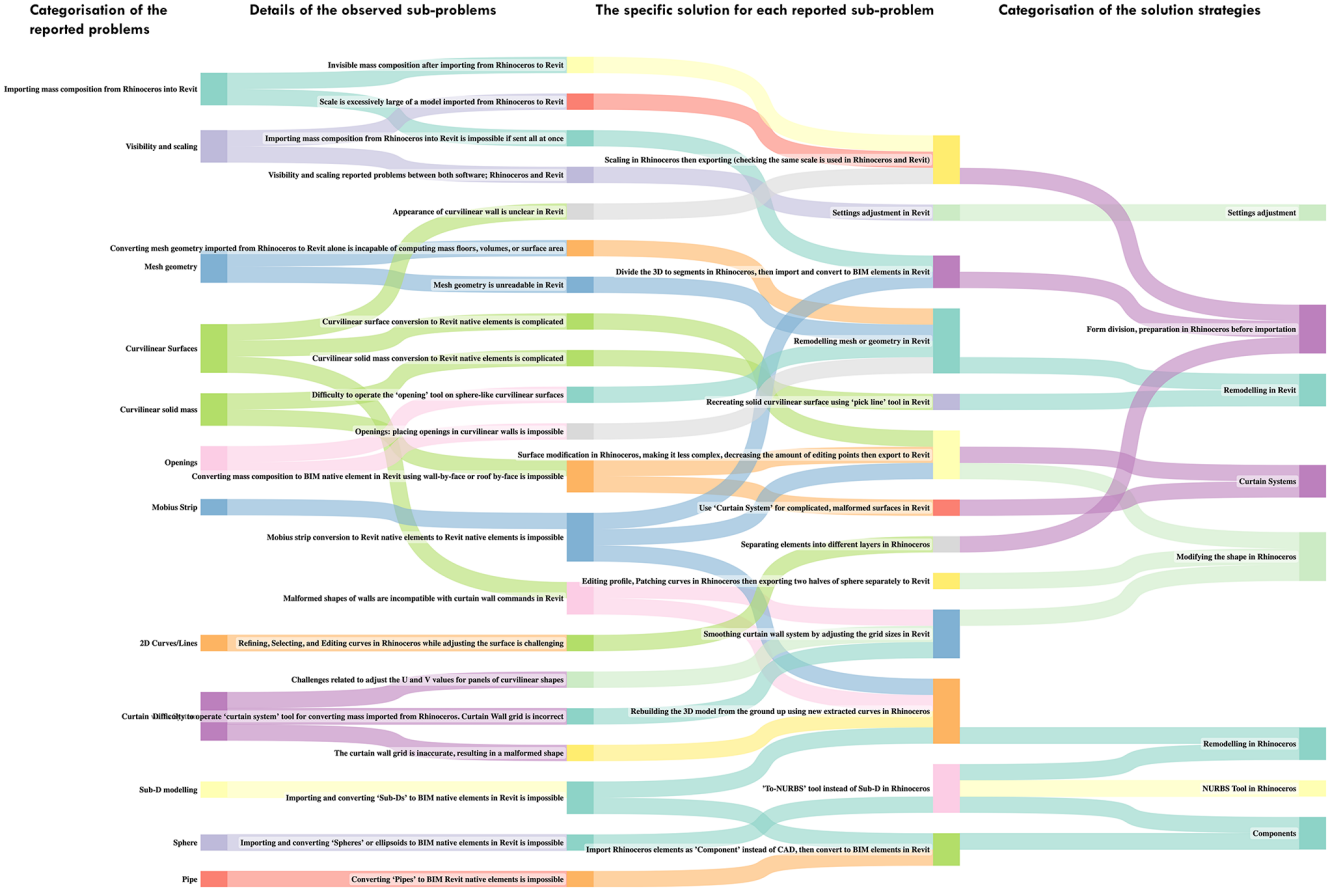
Flowchart of the interoperability procedure for converting elements between Rhinoceros and Revit modelling software. Source: authors.

The diagrams in Figs. 10 and 11 are derived from Table 4, highlighting interoperability challenges, problem classes, and corresponding strategies for resolution.

### Statistical analysis of student survey

The study assessed the effectiveness of the interoperability process in preserving original designs. Forty-five students from the Spring 2023 semester participated by completing a questionnaire which focused on whether the original design concept remained intact or underwent alterations during the conversion from Rhinoceros to Revit. In addition, they were asked to evaluate whether these changes positively or negatively affected the creativity of the initial idea. Furthermore, the study investigated how BIM/Revit elements were adjusted to comply with technical drawing specifications. The results are presented in Table 5, using a 1 to 5 scale for assessment.

**Table 5:** Reports on survey after students had completed the requirements for their digital projects, including interoperability and conversion from Rhinoceros to Revit:

No	Question	Result	Mean	Standard Deviation
1	Please evaluate the following statement regarding the conversion of the core project idea from Phase 2 to Phase 3 after the interoperability process has been implemented: "In your opinion, the interoperability process successfully converts the core project idea from Phase 2 to Phase 3." Please rate your agreement with the statement using the following scale: Strongly Agree Agree Neutral Disagree Strongly Disagree	60% of the students (27 out of 45) selected (agree), 22.2% (10 students) selected 3 (neutral), 17.8% (8 students) selected 5 (strongly agree)	9	11.05
2	Has a component of the project you created in Rhinoceros been completely altered?	51% of the students said that some components of their Rhinoceros-designed project had been entirely changed, whereas 49% said no	–	–
3	Explaining the previous response, what were the grounds for altering the original shape?	4 students answered: Changing the basic idea and design because the design was too simplistic; they therefore introduced some complexity by adding more details to the model	2	1.41
		21 students answered: Changing some parts of the three-dimensional composition components: Some of the components were created with the Sub-D tool in Rhinoceros and they were unable to export them from Rhinoceros to import them into Revit Some of the components, such as the pipes or spheres, were not transformed in Revit as wall, floor, roof, or curtain system, so they returned to Rhinoceros to rectify the shape problems Curvilinear shapes were redrawn to improve accuracy due to technical issues (thickness or the amount of control points) Only the scale was changed	5.25	2.5
4	Did you redo this item or items in Rhinoceros?	41% of the students said they remodelled the items in Rhinoceros, whereas 59% said they did not	–	–
5	Did you redo this item or items in Revit?	40% of students elected to redesign the objects in Revit, whereas 60% chose not to redraw the items	–	–
6	Explain why you wanted to redraw one or more elements in the Revit application. What are the reasons for abandoning Rhinoceros in favour of modelling this element in Revit?	10.7% disliked drawing elements in Revit; Rhino is more adaptable The Revit was 39.3% more accurate with straight lines and simple curves 50%: The Revit software made it easy to create elements such as Shaft on Floors, Structural Elements, components, and materials 21.4%: Many floors did not convert from mass to Revit elements because they were inaccurate in Rhinoceros and shafts could not be opened in them, so it was simpler to redraw them accurately in Revit 42.9%: It was difficult to redraw the structural parts (Wall, Roof, Columns) in Revit with precise measurements 28.6%: Finding all furniture and landscape elements in Revit was very simple 7.1% of students chose Revit materials because they are easier to assign and appear more natural	9.3	5.69
7	If you used any of the following in your Rhinoceros phase, please explain how you solved any problems and converted it/them into Revit elements. (Sphere, Pipes, and Sub-D)	In Rhinoceros, 56% of students used the ‘Pipe’ command, 29% used the ‘Sub-D’ command, and 15% used the ‘Sphere’ command		

Tables 6 and 7 discuss issues and solutions encountered when converting Rhinoceros elements using the Pipe, Sub-d, and Sphere commands into native Revit elements, and calculate the mean and standard deviation for each solution identified in a student survey.

**Table 6:** Report on issues encountered and solutions provided when converting Rhinoceros elements formed by the Pipe, Sub-d, and Sphere commands into native Revit elements. Source: Authors.

	The Problem	The Solution	No
Rhinoceros to Revit Conversion issues students faced and solutions they devised			
Sub-D	1-Could not export it from Rhino with extension (ACIS.SAT)	1-Convert it to (NURBS), then explode it before exporting it from Rhino	7
	2-Could not convert the exploded (NURBS) to (wall, floor, roof) when imported in mass because of the large number of parts	2-Import it again as a component	1
	3-Could not export it from Rhino with extension (ACIS.SAT)	3-Extract the outer curves and redesign the surface using (Loft)	2
Pipe	1-In Rhino the ground was made up of several pipes: a shaft could not be opened in Revit	1-Delete and redraw the floor in Revit	1
	2-Could not convert the pipes to (wall, floor, roof) when importing in mass	2-Import it again as a component	5
	3-Could not convert the group of pipes to (wall, floor, roof) when importing in mass	3-Explode them into separate items, then select each pipe and import it into Revit	4
	4-Could not convert the pipes to (wall, floor, roof) when importing in mass	4-Convert the Pipes to (Curtain System)	7
	5-Could not convert the pipes to (wall, floor, roof, and curtain system) when importing in mass	5-The scale was the problem After rescaling, convert the pipes to (Curtain System)	1
	6-Could not convert the pipes to (wall, floor, roof, and curtain system) when importing in mass	6-Split them into smaller sections, then reinsert in Revit	1
Sphere	1-Could not convert the Sphere to (wall, floor, roof) when importing in mass	1-Split it into quarters and mirror it in Revit after importing	2
	2-Could not convert the Sphere to (wall, floor, roof) when importing in mass	2-Convert the Sphere to (Curtain System)	2
	3-Could not convert the Sphere to (wall, floor, roof, and curtain system) when importing in mass	3-Import it again as a component	1

**Table 7:** Calculation of the mean and standard deviation for each solution identified in the student survey on converting elements built in Rhinoceros using the commands ‘pipe’, ‘sub-d’, or ‘sphere’ to Revit.

	Solution 1	Solution 2	Solution 3	Solution 4	Solution 5	Solution 6	Mean	Standard Deviation
Sub-D	7	1	2				3.33	3.22
Pipes	1	5	4	7	1	1	3.17	2.56
Sphere	1	2	1				1.33	0.58

### Strategies for interoperability and conversion issues in *Rhinoceros* 3D models to revit

This section investigates the challenges faced during the process of converting various elements generated in Rhinoceros using commands such as Pipe, Sub-d, and Sphere into their corresponding elements within the Revit environment. The conversion from Rhinoceros to Revit involves translating complex geometries and structures, often resulting in compatibility issues due to differences in software capabilities and native element types. We discuss the specific hurdles encountered during this conversion process and the strategies employed to overcome them. These solutions are essential for ensuring the accurate representation and integration of Rhinoceros designs into the Revit platform, facilitating seamless interoperability between the two software environments.

The interoperability process transitioned the project concepts from the conceptual to the BIM phases, with significant modifications originating in Rhinoceros. These alterations were organised into two categories: conceptual form transformations to adhere to fundamental principles, and adjustments due to technical issues arising during modelling. Major problems were addressed by various means, including remodelling parts and resolving form issues in Sub-D, Pipes, and Sphere.

Interoperability procedures between Rhinoceros and Revit were classified into technical, vision and scale, mesh geometry, curved surfaces, curtain wall systems, sub-D modelling, and domain and piping commands. The solutions encompassed form division, Rhinoceros preparation prior to adjusting importation settings, remodelling masses in Rhinoceros, modifying shapes in Rhinoceros or Revit, importing Rhinoceros masses into Revit as components, utilising NURBS Tool in Rhinoceros, and implementing curtain systems. Figures 10 and 11 illustrate these procedures based on records from thirty-five projects.

The study analysed the challenges encountered during migration from Rhinoceros to Revit, dividing them into primary and sub-divided issues. Solutions were identified for each sub-divided challenge, the most common being dividing elements into layers in Rhino (25%), and remodelling elements in Rhinoceros (15%). However, certain elements such as Sub-D, pipe, and sphere are difficult to convert unless segmented into basic components processable by Revit. Importing these items as components and defining their BIM category during importation is the preferred solution.

## Conclusions

In the realm of complex construction projects, effective data exchange between stakeholders is vital to project success and quality design outcomes. Interoperability, a cornerstone of digital design, plays a pivotal role in facilitating seamless communication and information management for the various engineering modelling programs utilised across disciplines.

Building Information Modelling (BIM) has emerged as a game-changer in digital architectural design and construction engineering, revolutionising project lifecycle management through integrated information exchange. BIM streamlines design, planning, and construction processes by offering multidimensional information models and robust analytical tools. Its implementation fosters enhanced communication, collaboration, and data exchange standards among stakeholders. The criticality of BIM lies in its ability to ensure accurate information transfer across project phases, mitigating time and cost losses incurred due to inadequate information during design and construction.

Interoperability serves as the linchpin for integrated project delivery within the BIM model, necessitating seamless integration of software and hardware systems. Despite its significance, the construction industry grapples with substantial challenges pertaining to systems interoperability. These challenges include difficulties in accessing accurate data, lack of interoperability between systems operating under differing standards, and constraints in program plans and designs optimised for a limited parameter set. It is imperative to address these challenges in order to enhance the efficiency and effectiveness of construction projects. Achieving interoperability between exchange formats is crucial to defining data interchangeability, encompassing shared meaning and data exchange.

Efficient data exchange and interoperability are critical for successful architectural design and construction engineering projects. Leveraging Building Information Modelling (BIM) technology and ensuring seamless interoperability across diverse software platforms is essential for enhancing communication, collaboration, and data exchange among stakeholders. It is imperative to address challenges in interoperability in order to streamline project workflows, minimise errors, and maximise project efficiency. The ongoing discussion on digital tools and methodologies in architectural practice is vital for driving innovation and improving project outcomes in the construction industry.

The integration of algorithmic modelling tools (Rhinoceros) with Building Information Modelling (BIM) tools (Revit) offers diverse workflows for efficient and intelligent design in the construction industry. Interoperability plays a pivotal role in transitioning between the BIM and construction phases, mitigating human error and reducing the costs associated with design modifications or production. Beyond drawing production, the BIM process interconnects the design stages, technical detail preparation, modelling, simulation, construction, maintenance, and time, financial, and facility management. It serves as a virtual platform, facilitating graphical and non-graphical data production, visualisation, processing, analysis, exchange, sharing, and maintenance in the AEC industry.

Various BIM application software packages, such as Autodesk’s Revit and Graphisoft’s ArchiCAD, are available. While some, like Graphisoft’s ArchiCAD, offer open language allowing for seamless information transfer between programs, others, such as Autodesk’s Revit, require addressing specific challenges to convert 3D algorithmic model elements into BIM elements. This paper contributes to the research field by providing experimental evidence for classifying and identifying different approaches, offering solutions to designer challenges during interoperability between Rhinoceros and Revit programs.

## Limitations

The sample size of 35 student projects may limit the generalisability of the results to broader architectural practices. However, the study’s strength lies in its in-depth qualitative analysis, offering a detailed understanding of the specific challenges associated with workflow interoperability. Future research could expand the dataset to include real-world projects to improve the external validity of the findings.

## Supplementary Information

Supplementary Information 1.

Supplementary Information 2.

## Data Availability

The authors confirm that the data supporting the findings of this study are available within the article and its supplementary materials.
