# Supplementary material for: Preserving conceptual design integrity: strategies for enhancing interoperability in architectural digital design workflows
Source: Sci Rep. 2024 Dec 23;14:30595. doi: 10.1038/s41598-024-78640-8 (PMC11666589; doi:10.1038/s41598-024-78640-8)
Supplement: Supplementary file 1 — Supplementary Information 1. [file 41598_2024_78640_MOESM1_ESM.pdf]

Final Project: Rhino-Revit Interoperability Paper

ARCH 273/1521 - Digital Rep Tools for Architects (Spring 2022)

Dr. Mariam Abuhadid - Section 85

Kariman R. Mustafa

900211922

May 26th, 2022

Introduction: Recalling layers & features of the project before applying the interoperability process in Revit

Before applying the interoperability process in Revit, I wanted to recall the project's features to understand what I will be aiming to do in Revit to fulfil the requirements. For example, I knew that the outer shells for the Mobius curves would be determined as regular walls, and the inner surface determined as a curtain wall. To complete the same flow, I will also be identifying the dome as a curtain wall while making its panels identified as mullions in Revit.

Moreover, I made sure to refamiliarize myself with the various layers I created on Rhino, such that it is easier for me to reach out for curves/surfaces when necessary. One thing that I kept in mind as I recalled the project was the scale of my building relative to a human being. I started by creating the silhouette/dimensions of a human (0.6 x 0.6 x 2) and comparing it to the building. Then, using the dimensions in Rhino, I measured the building's width, which was approximately 170 meters; therefore, I tried to create a reference line of one hundred meters and rescaled the building, accordingly, as illustrated in Figure 1.

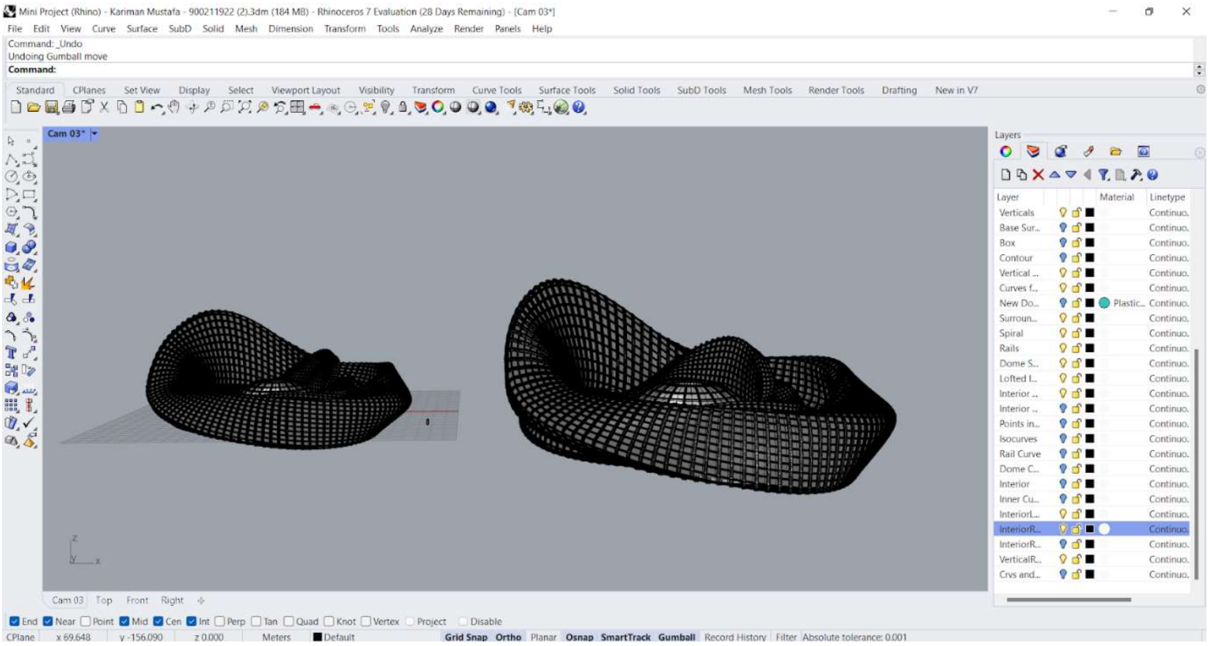

Figure 1. Rescaling the model

Applying the interoperability process

I changed the units to Meters in the Revit File to obtain the correct measurements, as per usual (Figure 2).

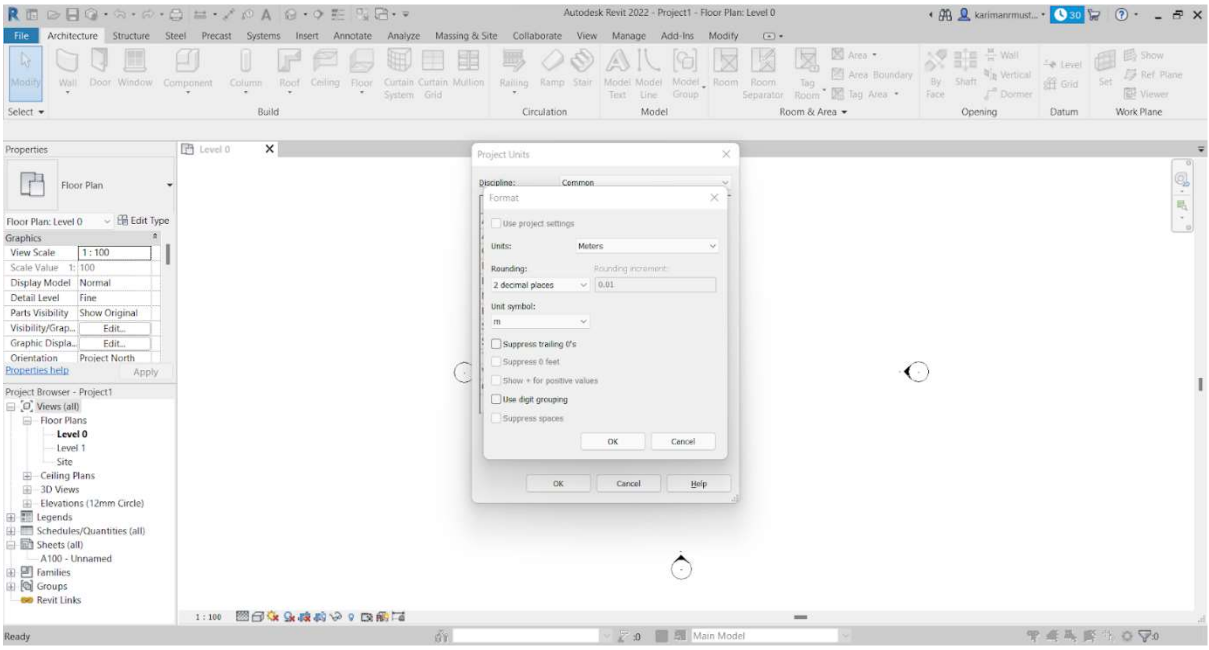

Figure 2. Changing units to meters on Revit File.

I. Dome

I started by exporting the walls of the dome. I removed the Plastic material I had on Rhino and exported the selected surfaces. Using the previously taught exporting method, I exported the selected surfaces as ACIS.sat. I inserted the mass to Revit from Massing & Site, clicked on In-Place Mass, and inserted the surfaces. Since the surfaces should be identified as curtain walls, I had to create a curtain system. I selected the surfaces and pressed “Create System.” However, Revit has warned me that the panels in the curtain system might be malformed (Figure 3).

Dome: Challenge 1 - Curtain System

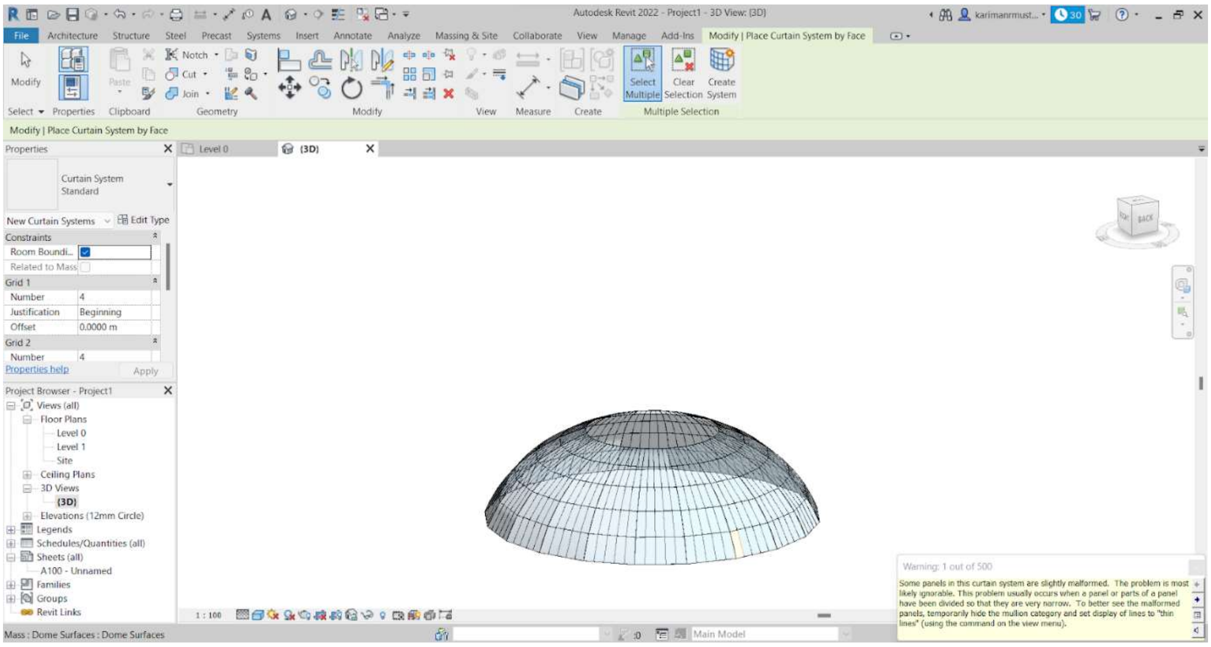

Figure 3. Revit Curtain System Warning

I was not exactly sure how to fix this; therefore, I searched for how I could fix this on the internet. Fortunately, I found a similar video on YouTube (Link: https://www.youtube.com/watch?v=z_iBVx1lSs8&list=LL&index=2&t=479s) that delves into the process of creating more complex curtain wall and wall systems using simple In-Place massing techniques. I determined that I had to decrease the spacing between the curtain wall grids to obtain a smoother surface, as shown in Figure 4.

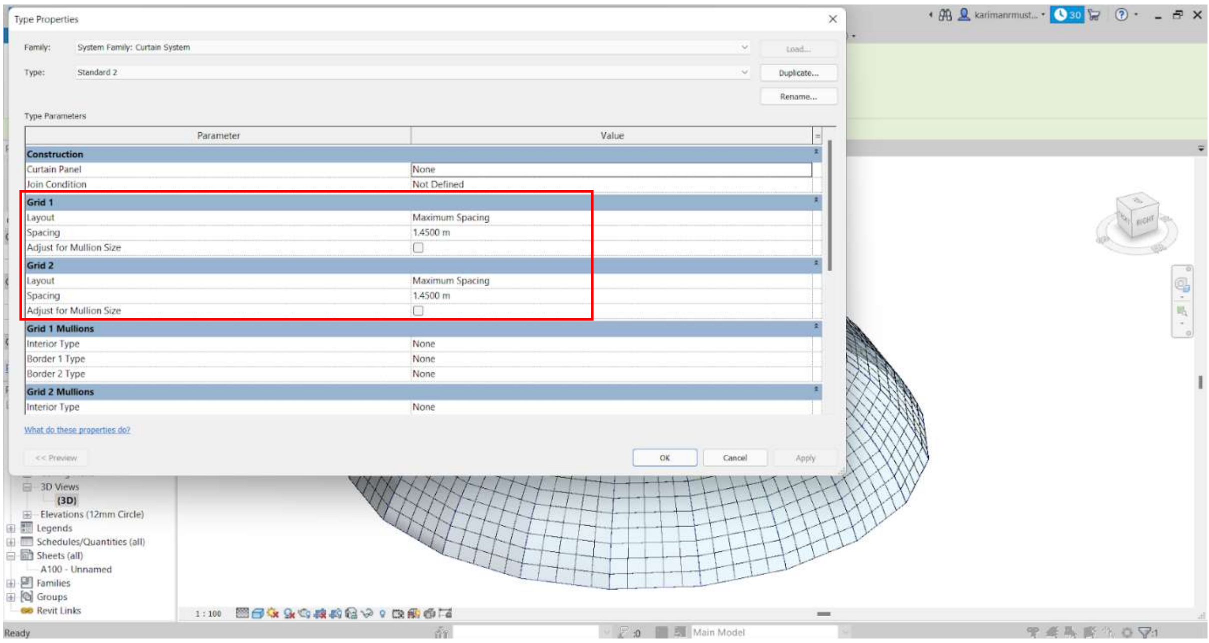

Figure 4. Changing grid spacing

Thankfully, this adjusted the curtain grids on the dome. Then, I selected the Vertical Rectangular mullions and exported them to Revit, as shown in Figure 5.

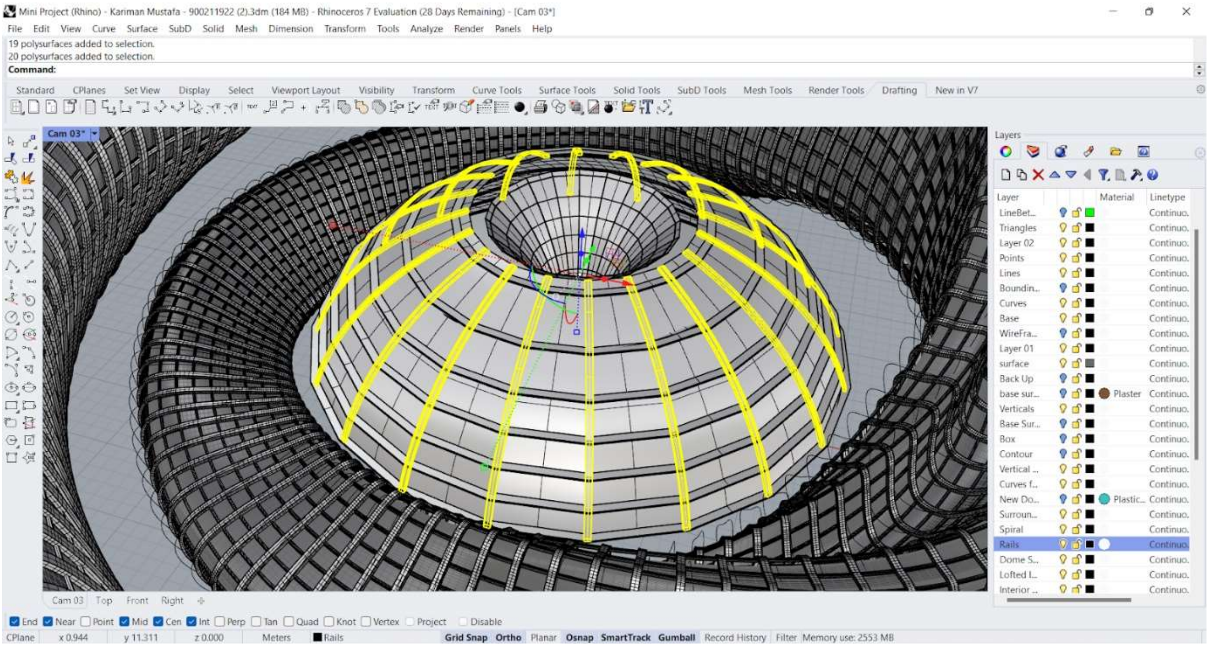

Figure 5. Exporting dome mullions

Using the exported Mass, I built mullions that fit the same structure and shape, then used the rectangular instead of the circular mullions and chose the mullions to be created along the vertical grid line; I applied the same steps as advised in this link: https://knowledge.autodesk.com/support/revit/learn-explore/caas/CloudHelp/cloudhelp/2019/ENU/Revit-Model/files/GUID-234F69C3-516A-4866-91D7-81B762170D4E-htm.html#:~:text=Click%20Architecture%20tab%20Build%20panel,across%20the%20entire%20grid%20line

I adjusted the size of the mullion to be the same as that of the exported Mass. I tried changing the width on both sides in the dimensions panel, but it seemed as though it would only change the dimension of the mullions from one side, as shown below in Figure 6.

Challenge 2: Dome - Mullions

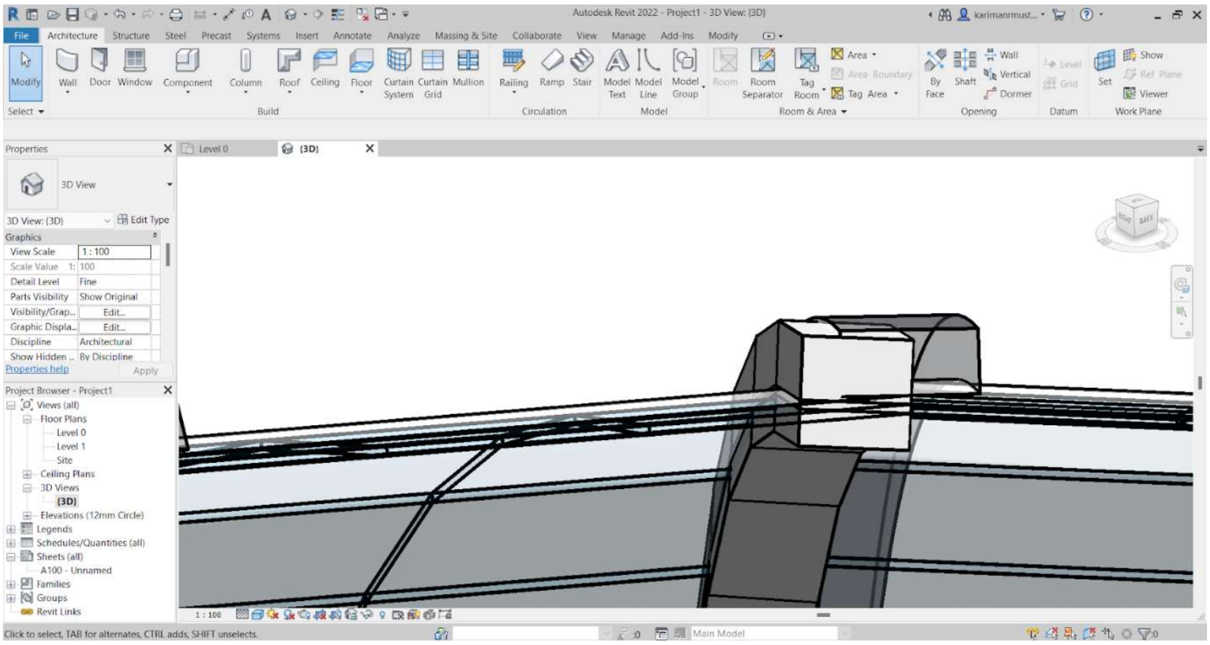

Figure 6. Issues related to mullion size & orientation.

However, I thought of only changing it from one side and then duplicating it to the other side to obtain the correct mullion size, as shown below in Figure 7.

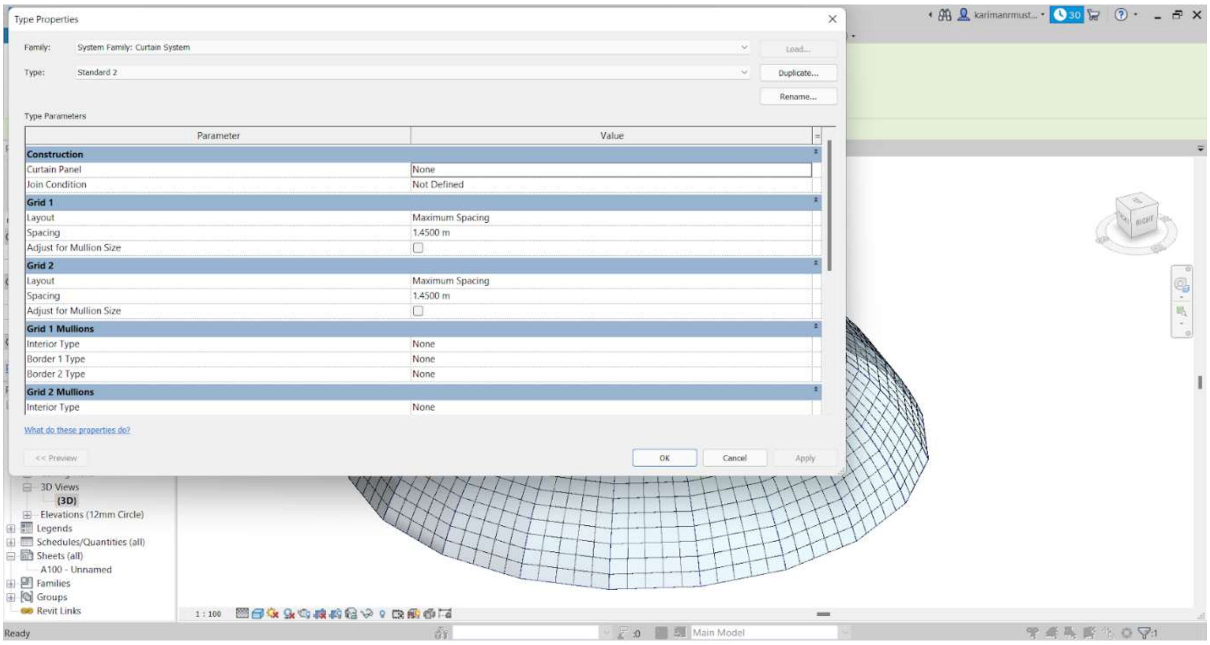

Figure 7. Rectangular mullion dimensions adjustment

Then, I exported the horizontal walls, as illustrated in Figure 8.

Challenge 2: Dome - Horizontal Walls

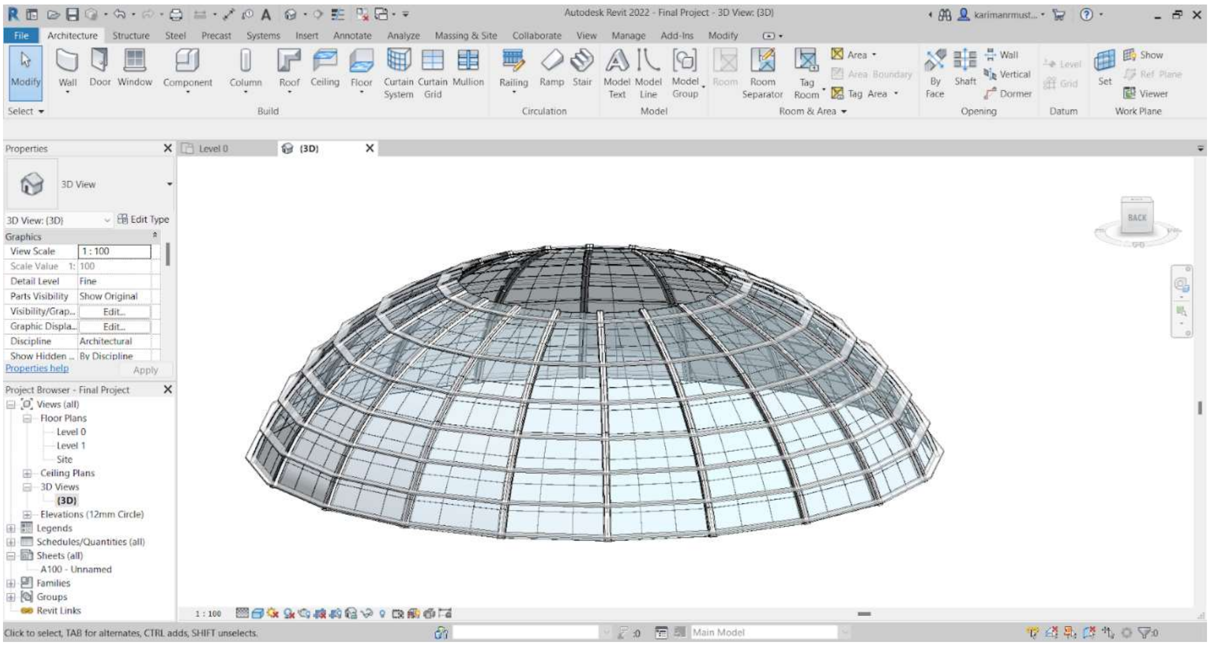

Figure 8. Exported horizontal dome walls from Rhino.

As I was trying to identify the horizontal walls as mullions, they affected the vertical mullions due to the connection; therefore, to remove this connection, I clicked on the command “Break at Join.” While this worked, I had to figure out a way to move the mullions so they aligned with the mass; since the grid lines of the curtain wall did not align with the horizontal walls, I tried to unpin the mullions to move them; however, the unpin option was not available, as shown in the views panel below in Figure 9.

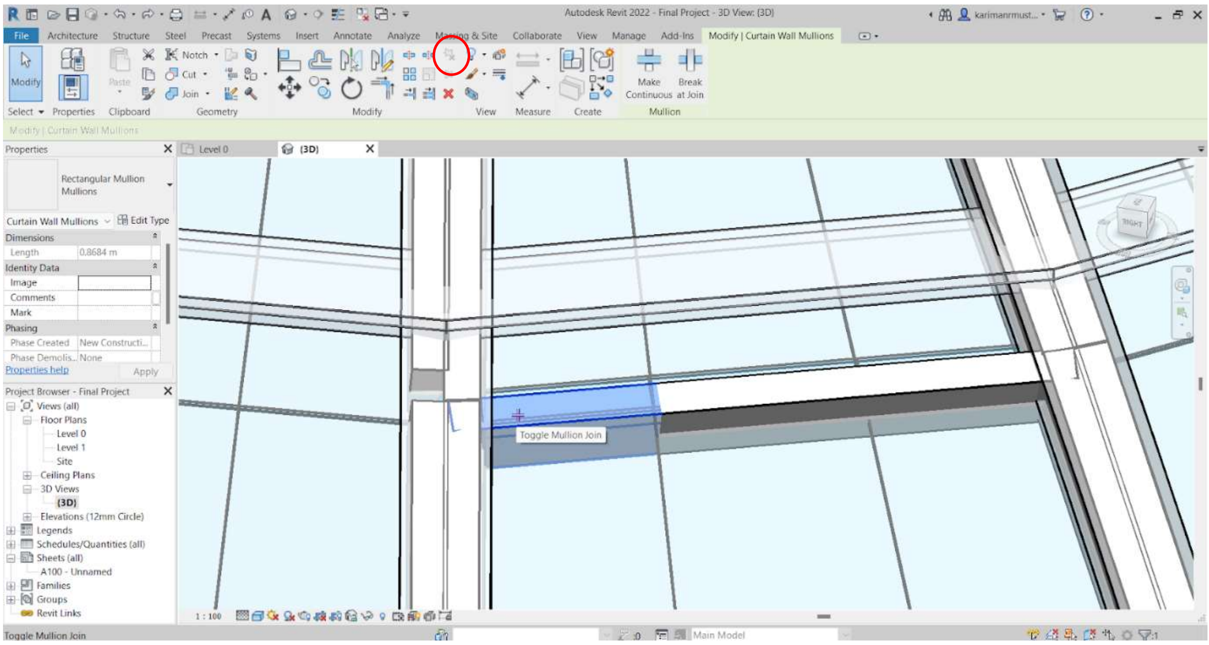

Figure 9. Inability to create horizontal mullions.

Therefore, I thought it would be best to identify the horizontals as Wall by Face. Once I did that, the walls overlapped, resulting in inaccuracies, as indicated by Revit in Figure 10.

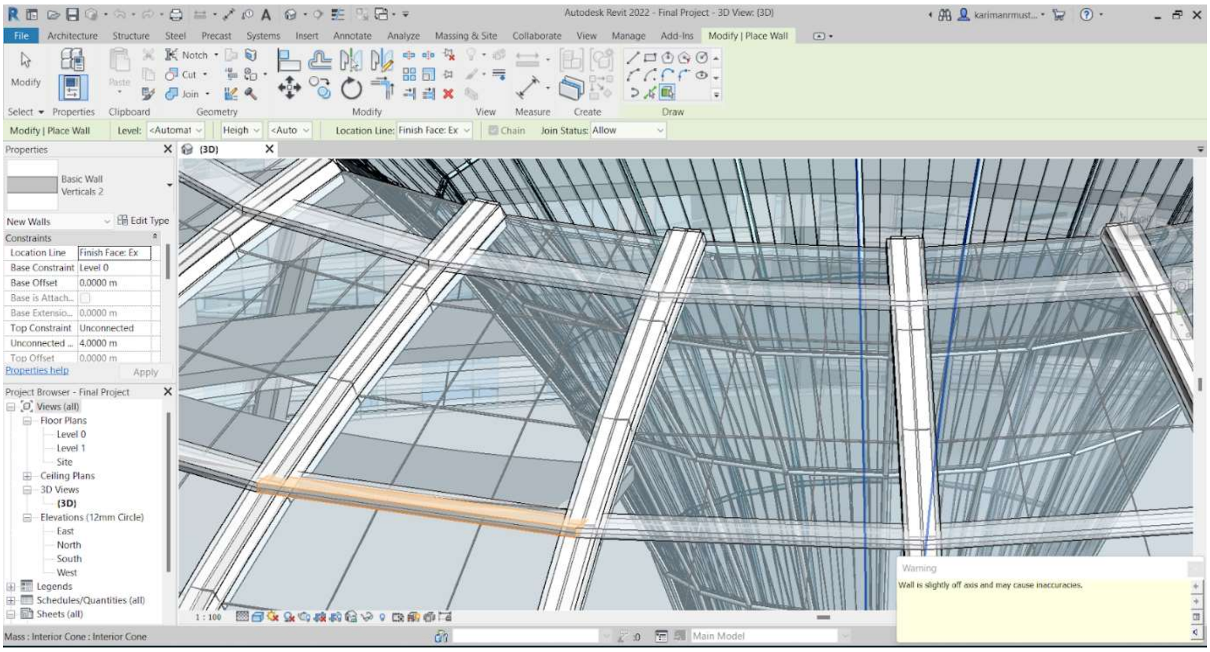

Figure 10. Overlapping walls after using Wall by Face command on Revit

However, at the same instance, a message appeared on Revit stating that I could use the Cut Geometry command to cut away the overlapping walls. Unfortunately, after doing as advised by Revit, the Cut Geometry command was not accurate, resulting in cuts in the actual wall, as shown in Figure 11.

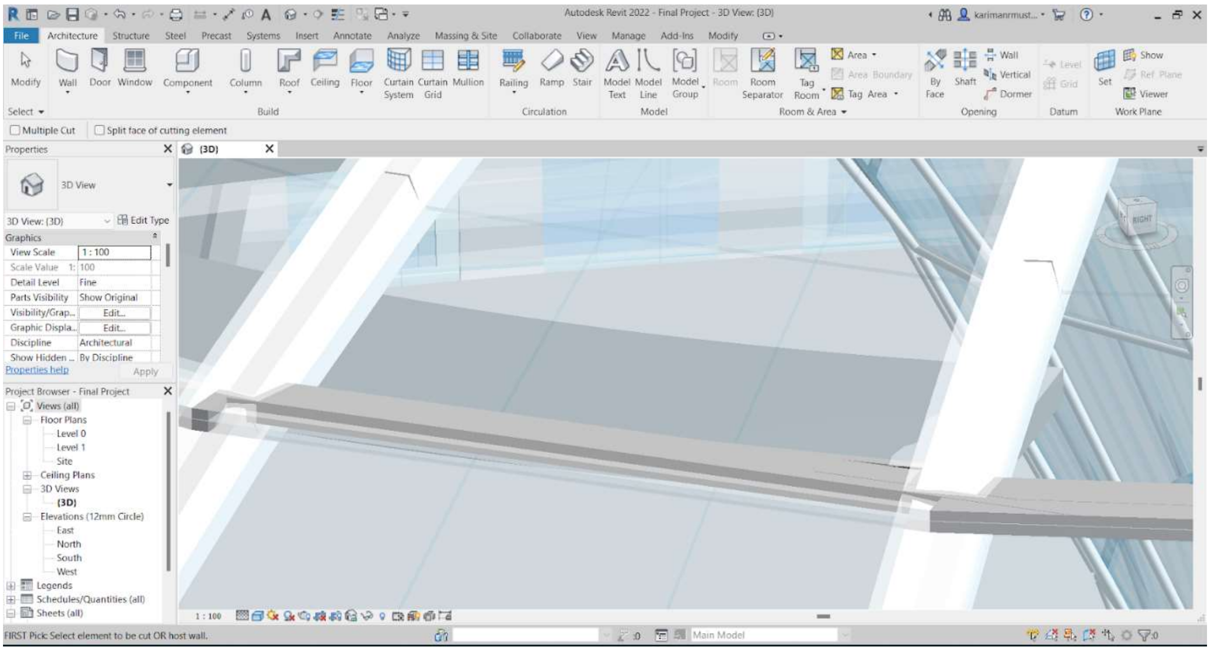

Figure 11. Results after using the Cut Geometry command on Revit

Therefore, I tried contacting one of the TAs to figure out a solution to the walls; we concluded that the best way to identify the mass would be Roof by Face. Personally, this was much easier and straightforward by which the mass could be identified in Revit (see Figure 12).

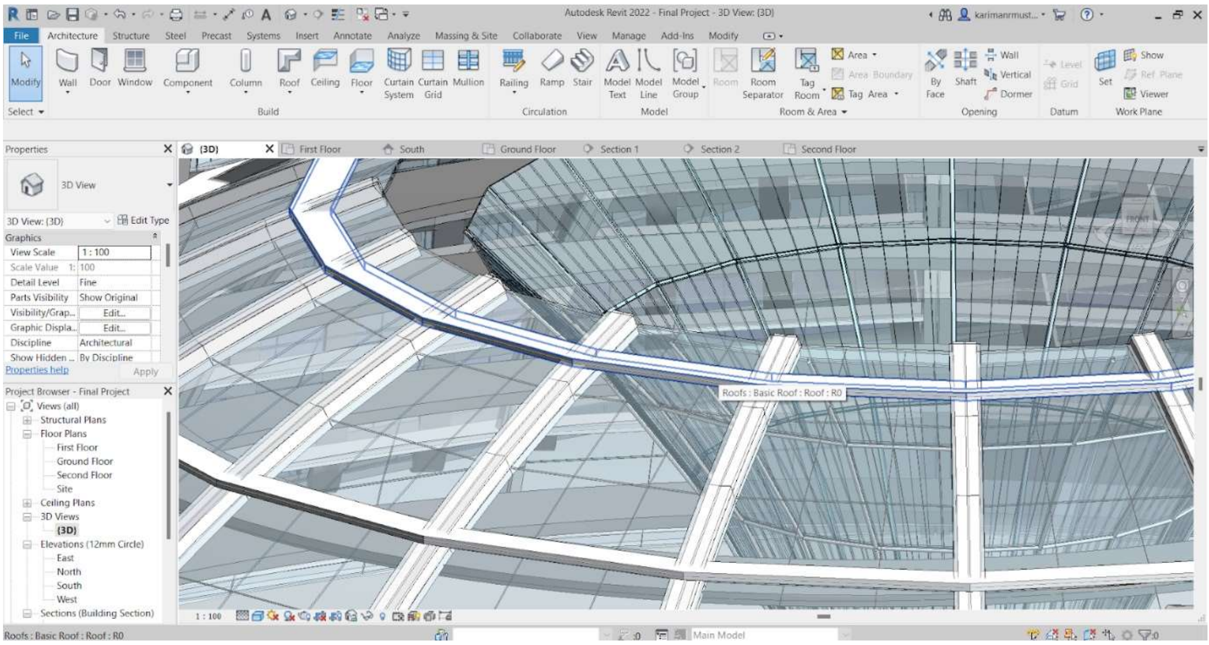

Figure 12. Identification of mass as Roof by Face

The last element regarding the dome was the inverted cone. I selected the inverted cone surfaces with their mullions and exported them to Revit (Figure 13).

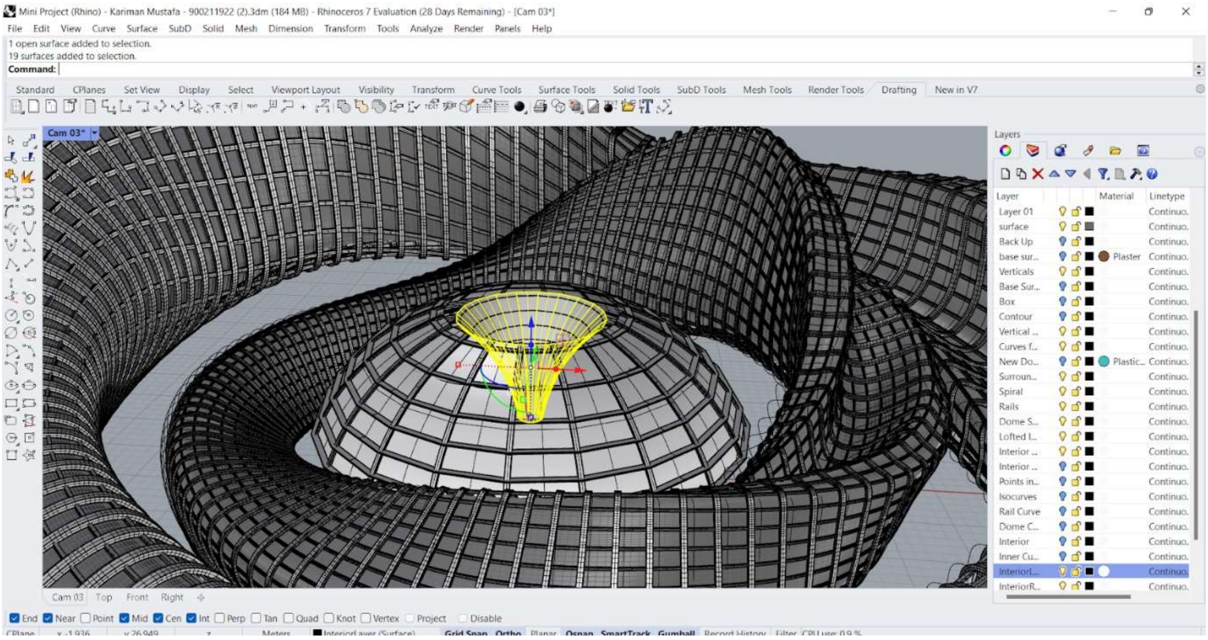

Figure 13. Exported Inverted Cone

The cone surface was then changed to curtain walls; the circular mullions on Revit were created using a 25mm Radius circular Mullion Size (Figure 14).

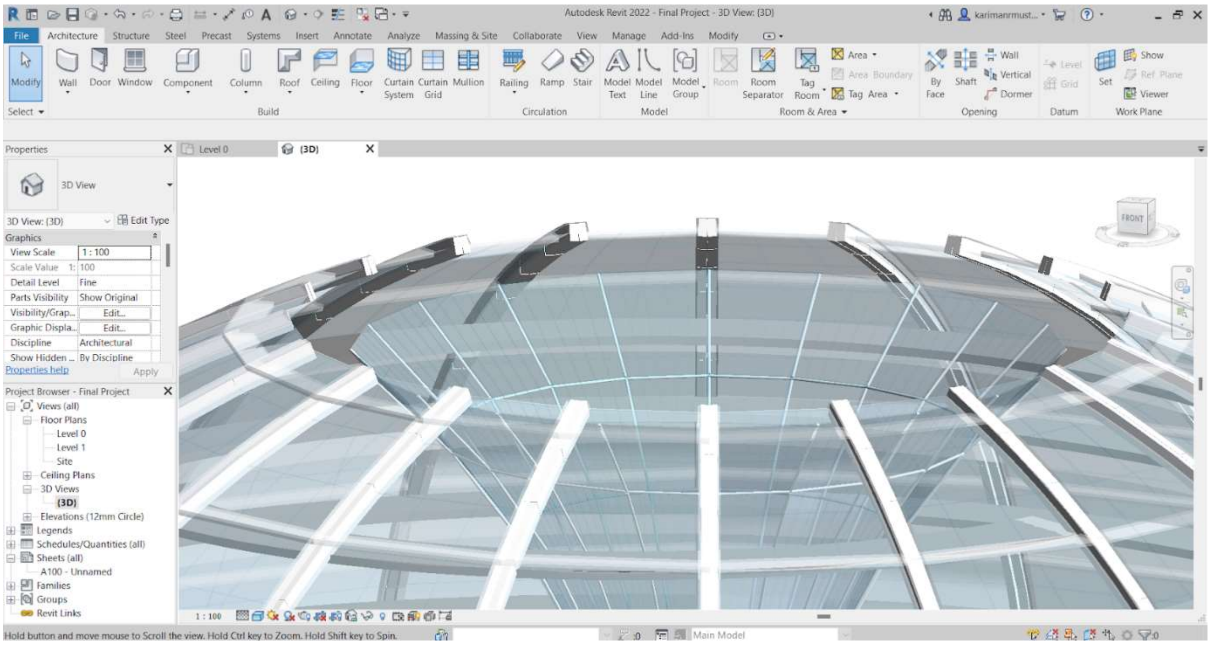

Figure 14. Inverted cone identified as curtain wall

II. Mobius Curves: U and V Panels

After placing the dome in Revit, I tried to export the horizontal curves/shells for the smaller Mobius Curve. However, Revit was not responding to all the curves at once, as shown in Figure 15.

Challenge 5: Not Responding to Horizontal Panels

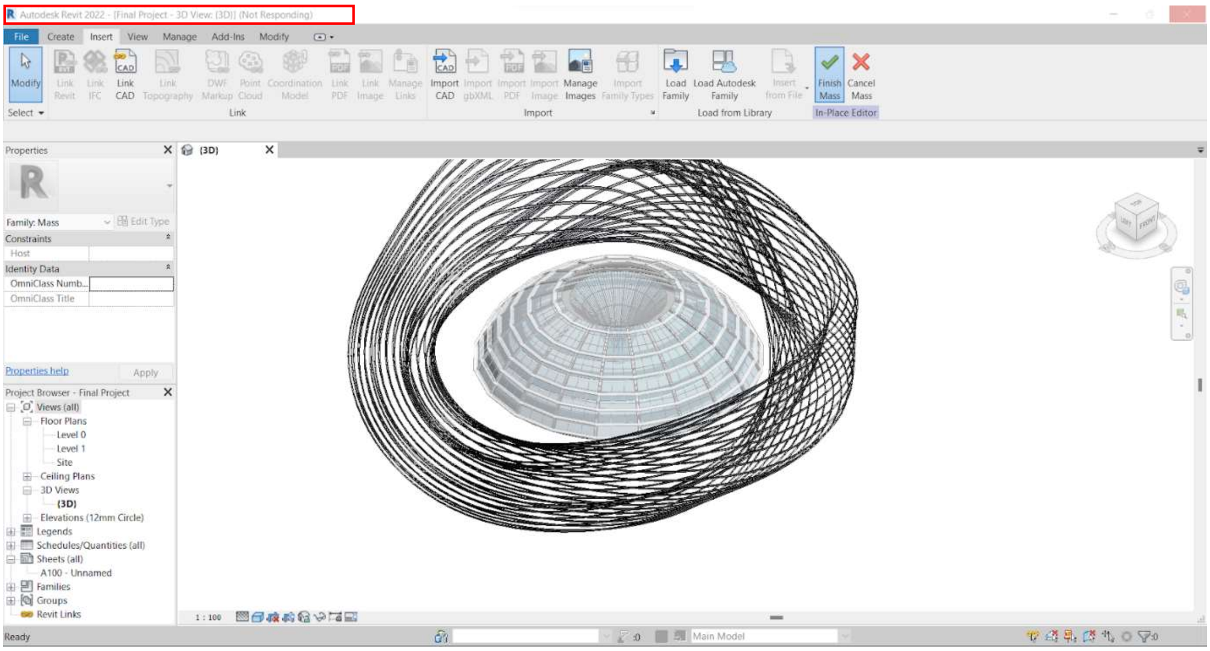

Figure 15. Revit not responding to exported mass

I decided to export the curves by dividing them into sets instead of exporting them all at once. Once I was done, I would change the color of the previous exported set to make it easier to follow up (Figure 16). The walls were created using Wall by Face (Figure 17).

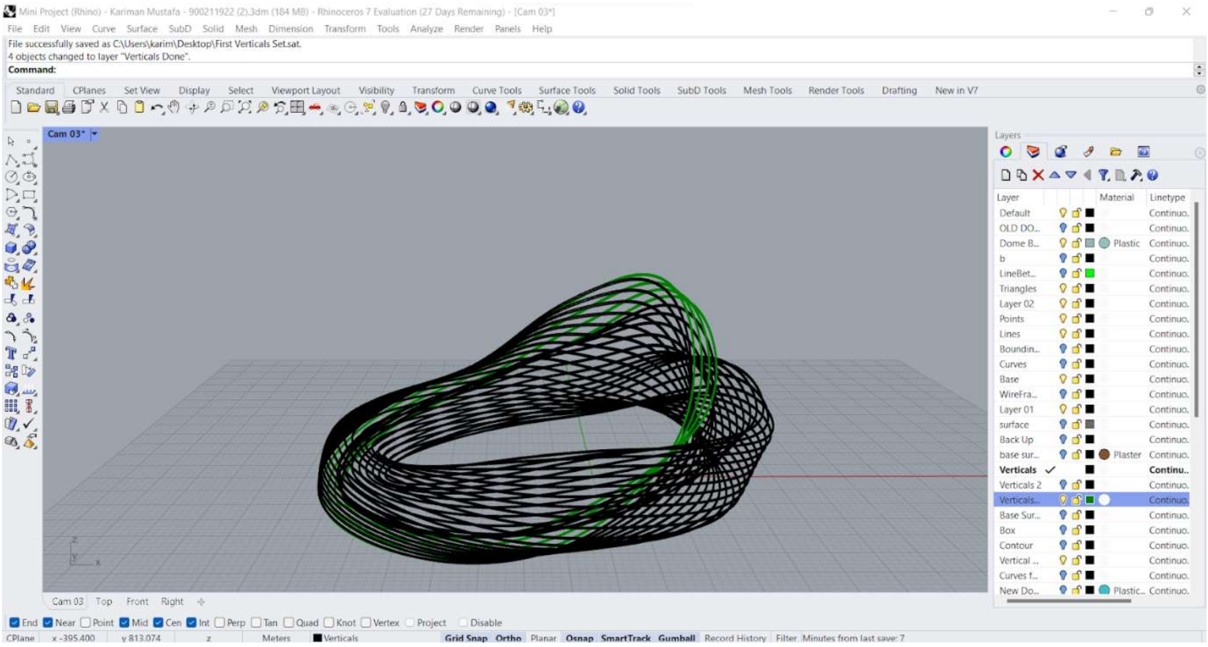

Figure 16. Set exported to Revit

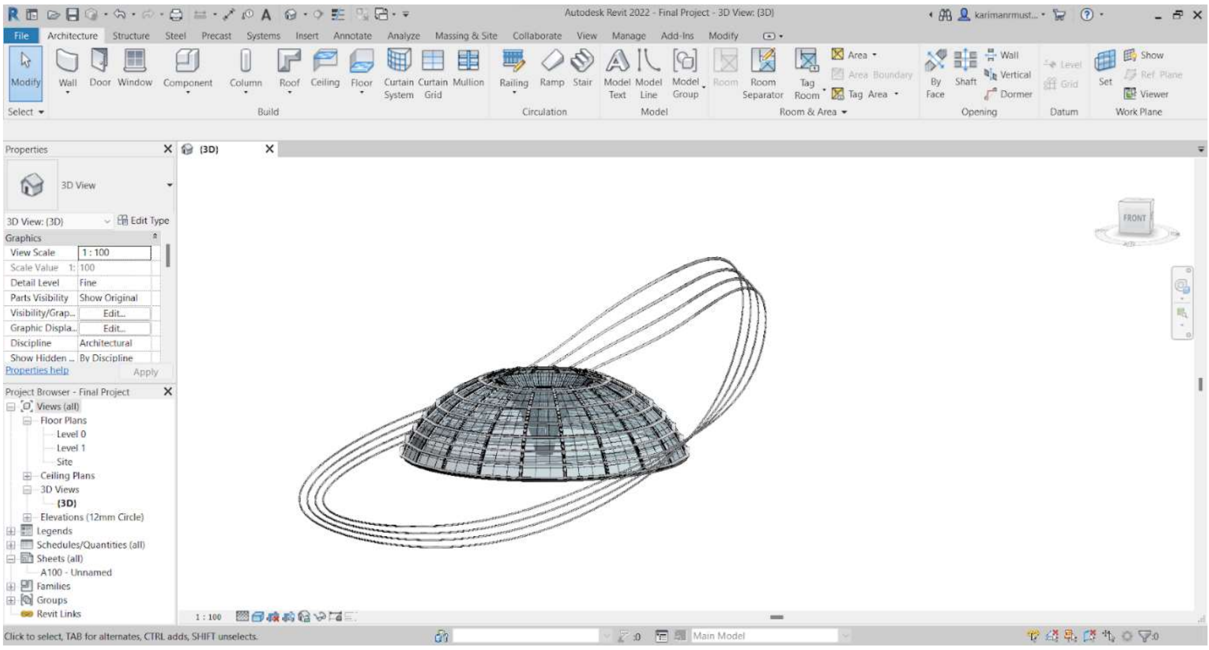

Figure 17. Applying Wall by Face

After completely exporting and giving the walls of the inner/smaller Mobius geometry an exterior finish, I moved to the outer curve, following the same steps (Figures 18 & 19).

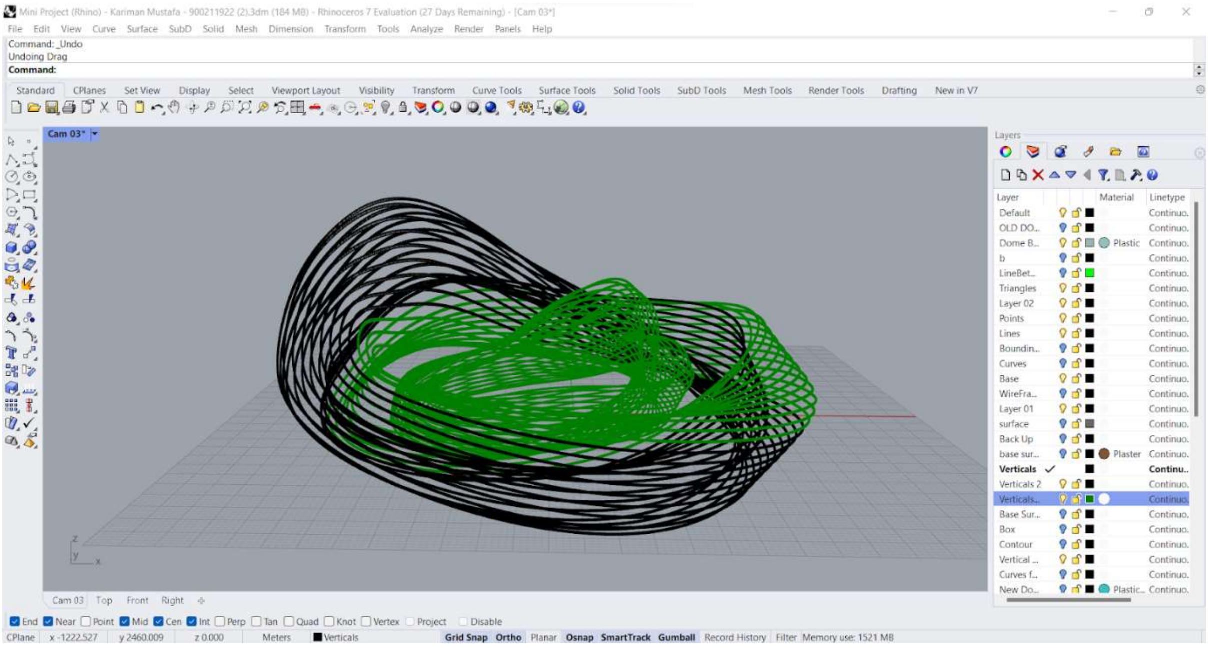

Figure 18. Exporting the larger geometry using a similar approach

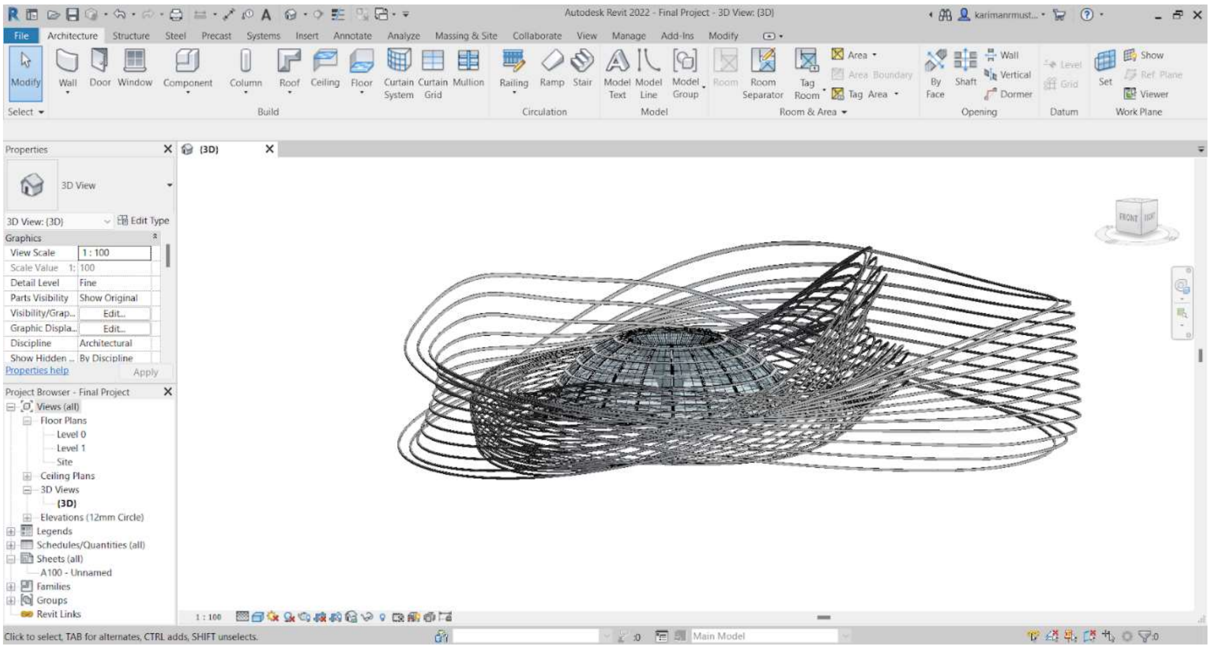

Figure 19. Wall by Face for Horizontal Curved Walls (0.15 m thickness)

I applied the same approach to the vertical panels/shells using the sets approach (Figure 20) and exported them into Revit, giving them a thickness of 0.3 meters and a default wall material.

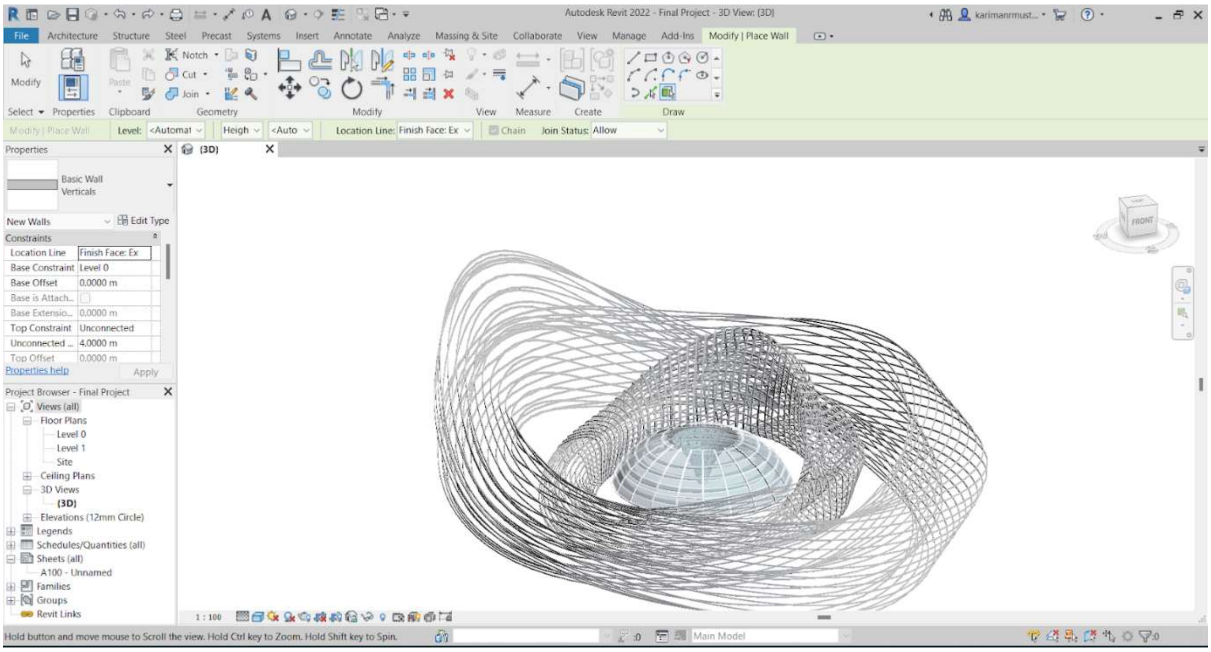

Figure 20. Midway process of providing thickness to walls.

Once all the sets were added, the results were as shown in Figure 21.

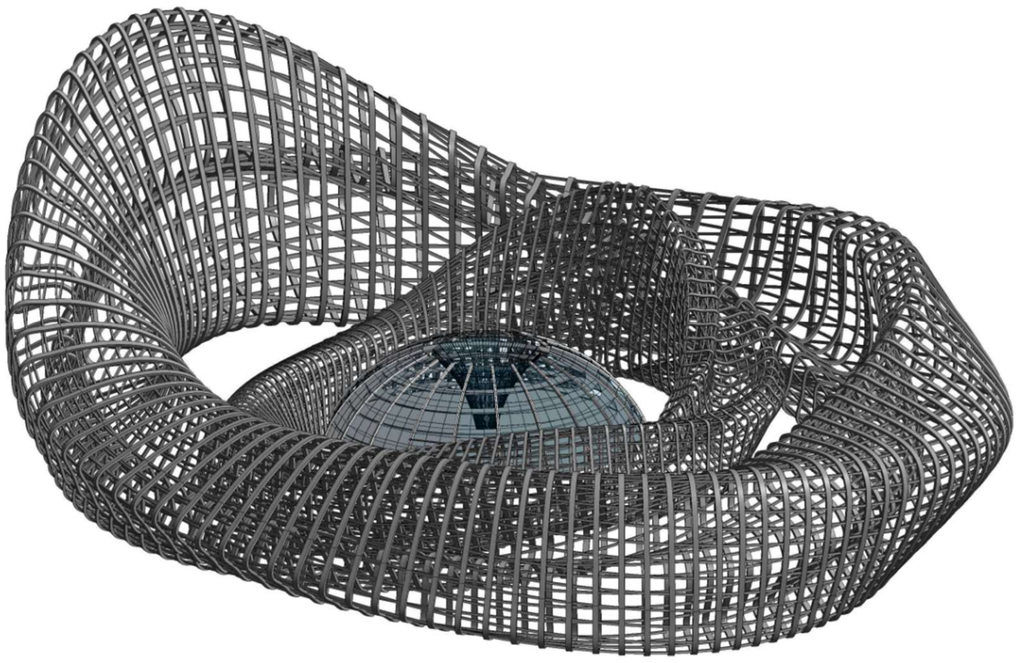

Figure 21. Model in Revit after inserting walls.

III. Mobius Inner Surface

The last step in the interoperability process of extracting from Rhino to Revit was the surfaces between the U and V panels. I tried to select the curves and export them to Revit; however, I would receive this message (Figure 22).

Challenge 6 - Surface: Empty Family

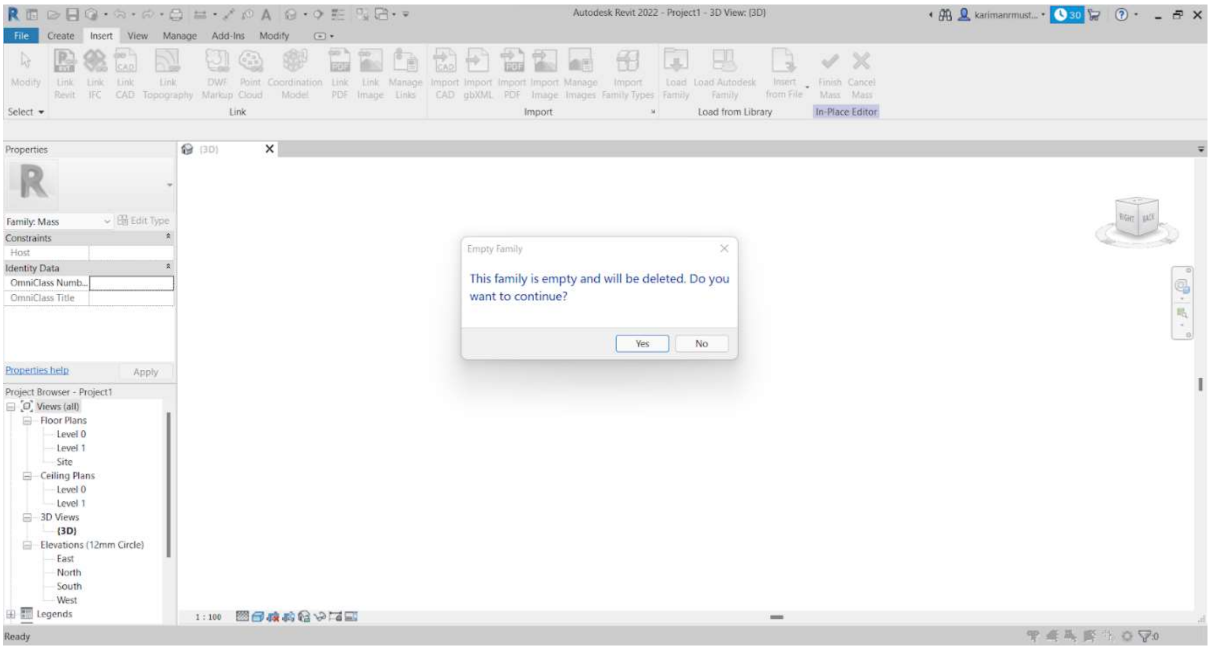

Figure 22. Message from Revit when exporting one of the surfaces.

I was not exactly sure what this meant; I tried searching it up on google, but I would not find anything relating to Revit. I pressed OK to continue, but nothing was exported to the Revit file. I then tried another approach, where I would transform the meshes of the surface into a Simpler Mesh on a Rhino; however, Rhino could not extract the surface as an ACIS.sat (Figure 23).

Challenge 7 - Surface: Mesh

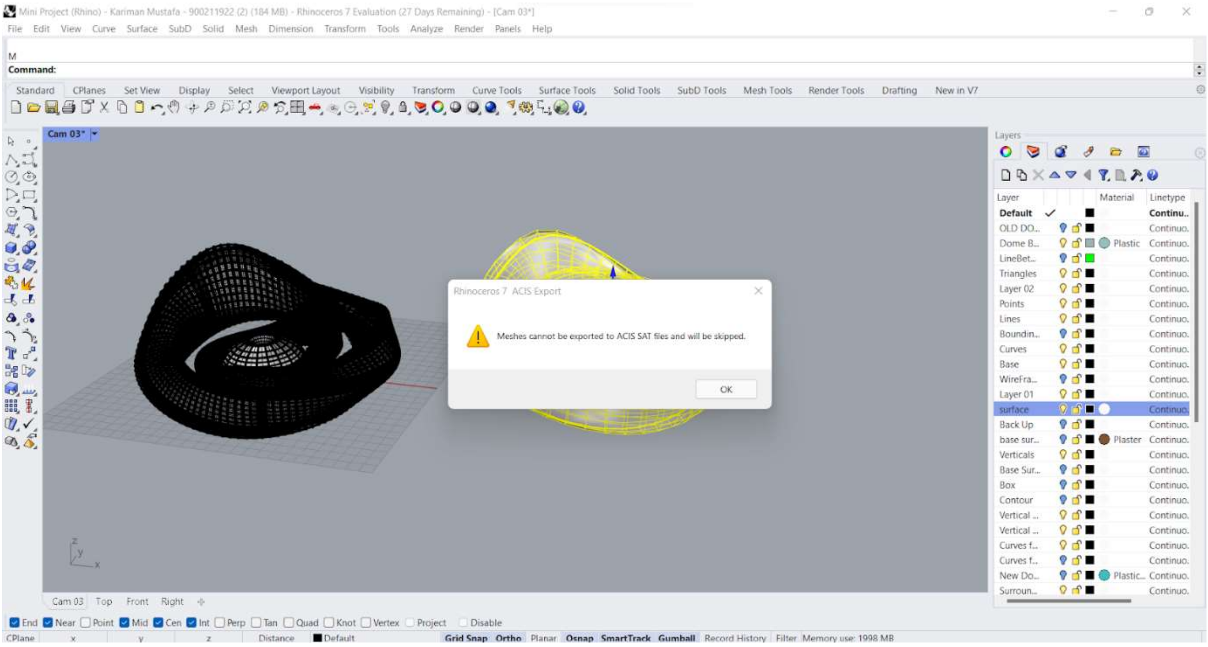

Figure 23. Message from Rhino when exporting the new surface

Then, I thought that the issue could be related to the complexity of the shape; consequently, I tried to cut the surface into parts so that the complexity of the surface was reduced. I assessed this out on a separate Rhino and Revit file (Figure 24).

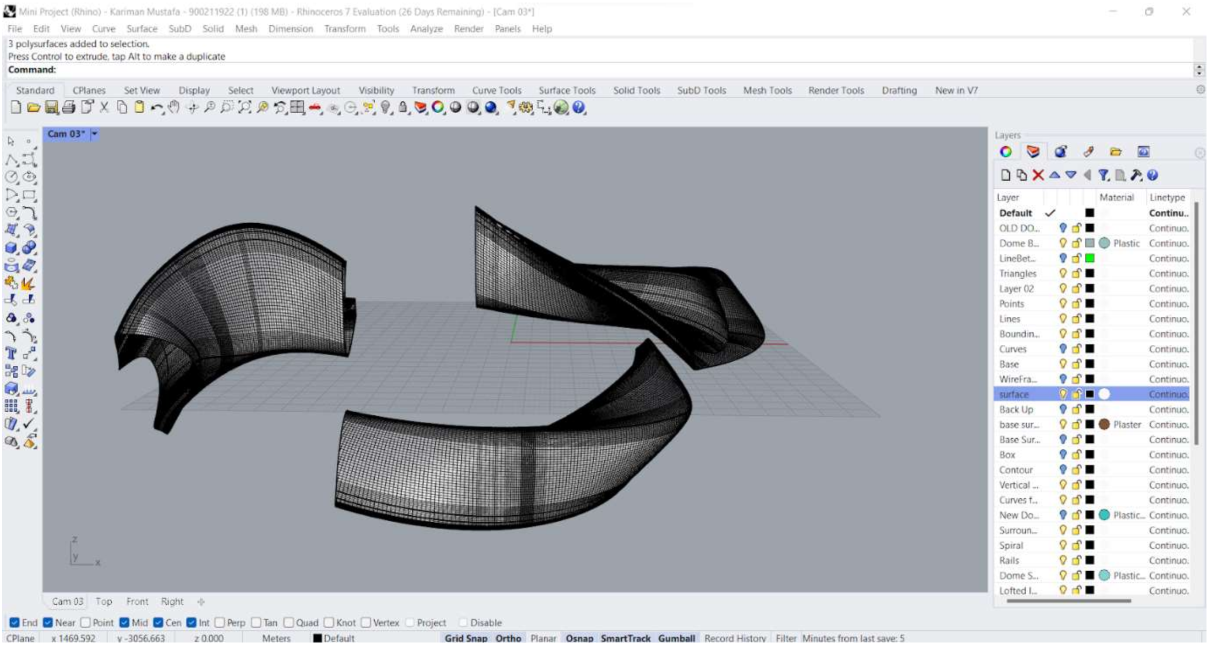

Figure 24. Separated surfaces to reduce complexity.

Afterward, I exported only one segment to the Revit file; unfortunately, I received the same message as shown previously in Figure 22. I tried searching on Revit to see if I could load the family, which is portrayed by Revit as empty, as shown in Figure 25. However, the family did not support .sat files (Figure 26).

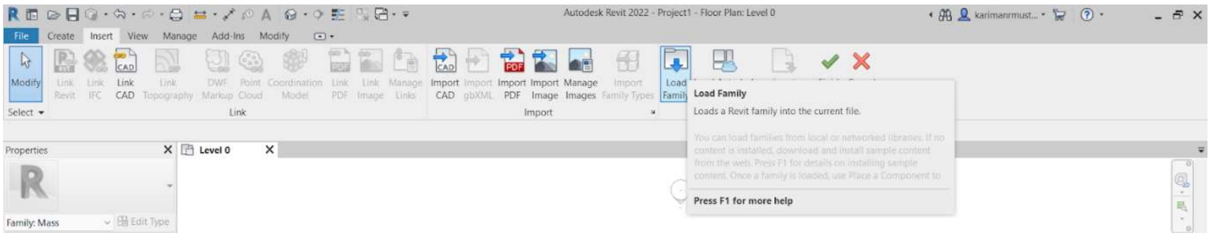

Figure 25. Load Family option to help insert curves

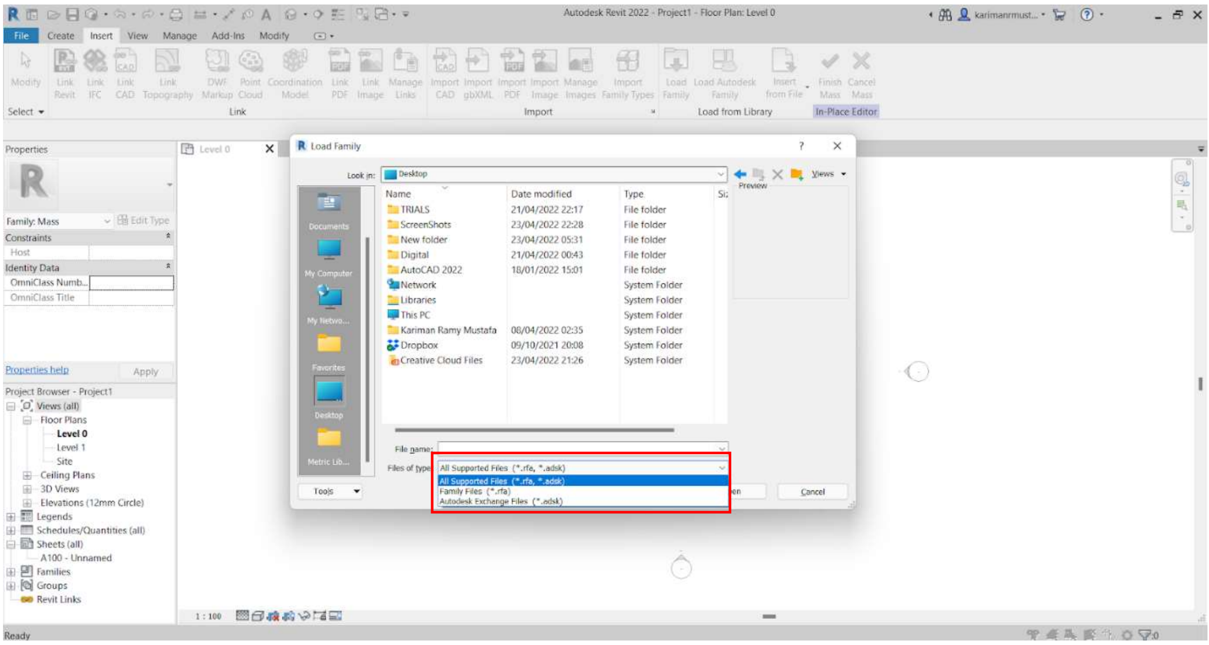

Figure 26. Unsupported .sat file

I tried to extract curves from the original surface, assuming that I could loft the surface again by extracting the wireframes or the isocurves; however, the cage edit that was previously applied to the surface as a part of the structure in Phases 1 & 2 of the Mini project has transformed the curves of the surface into open curves; thus they could not be lofted.

The last option I had that would potentially produce a remarkably similar curve was to select the horizontal curves that formed the exterior panels (on a separate layer), then re-loft the surface, hoping that the complexity of the surface was reduced. I turned off all other layers and lofted the surface, as shown in Figure 27.

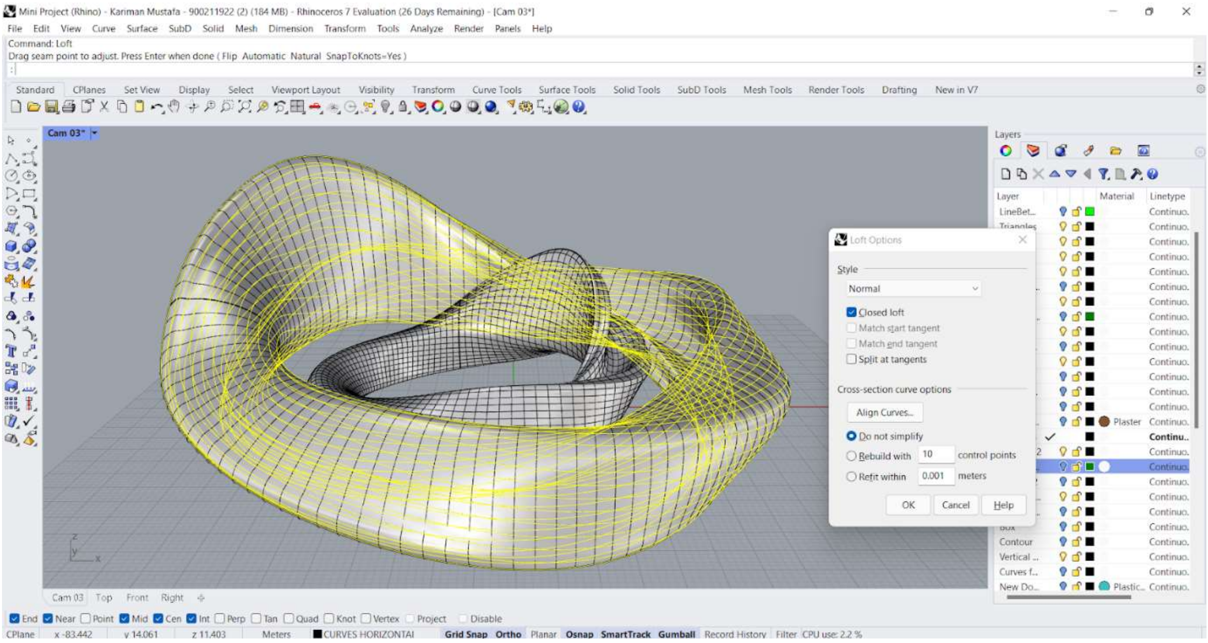

Figure 27. Lofting the Outer Mobius Geometry (Closed Loft)

After lofting the curves, I extracted the inner surface first, and thankfully, it was placed in Revit as desired. Then, to create the curtain walls, I requested Revit to create a Curtain System; however, the spacing between the curtain grids was incorrect, leading to random glass panels (Figure 28).

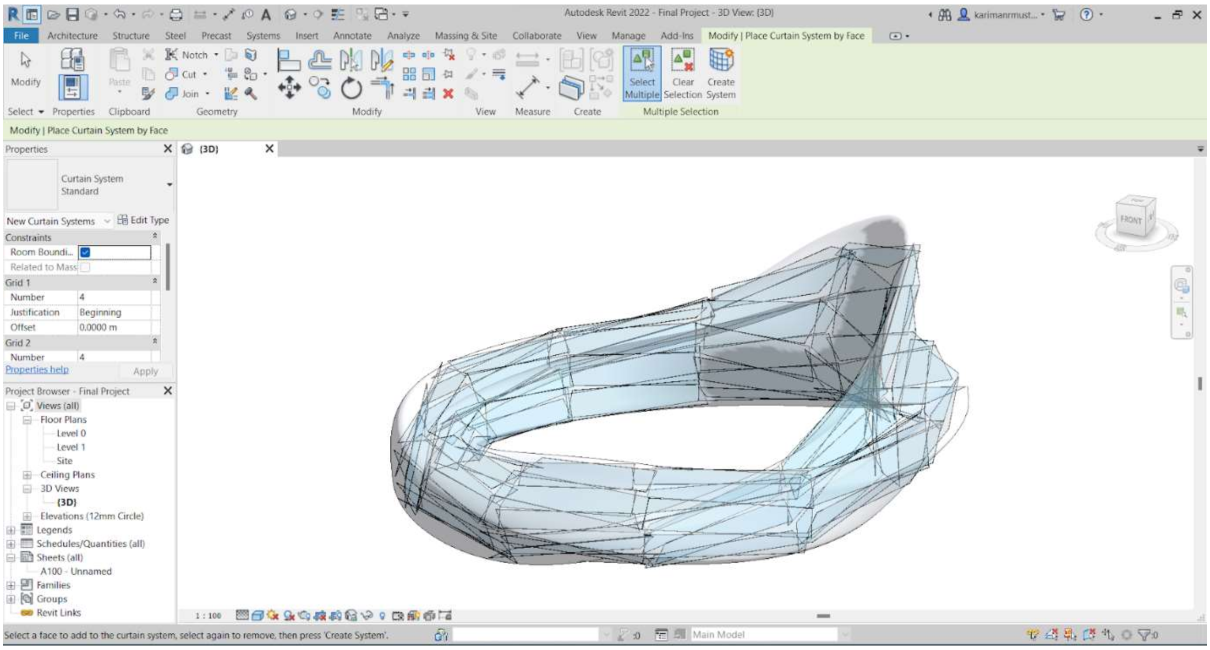

Figure 28. Random Glass Panels Created

Using the same Grid adjustment approach for the dome, I had to adjust Grids 1 & 2 by decreasing the distance to make the glass panels look smoother. Thus, obtaining the outcome in Figure 29.

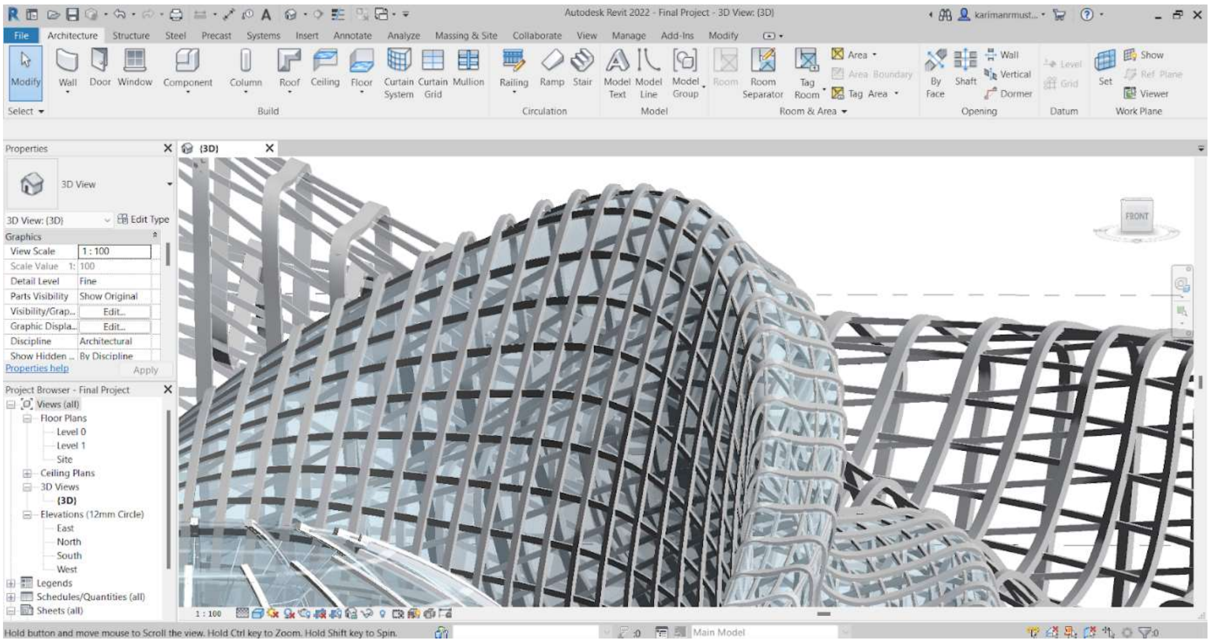

Figure 29. Changing Grid Spacing

Then, using the mass floor option, I requested Revit to create Floors inside the surface mass and then change these mass floors into architecturally defined floors (Floor by Face), as seen in Figure 30.

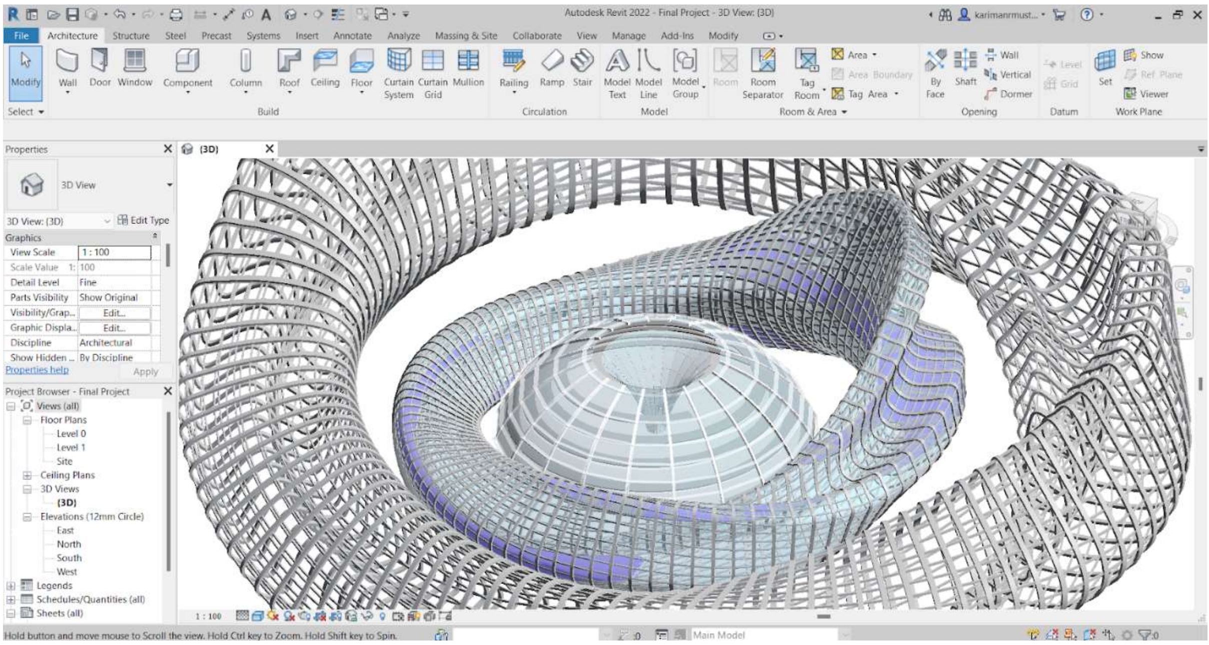

Figure 30. Adding mass floors from Revit and changing them to architectural floors

Doing the same for the Outer Mobius, the overall model exported from Rhino to Revit was fully identified as curtain walls, walls, floors, roofs, and mullions (Figure 31).

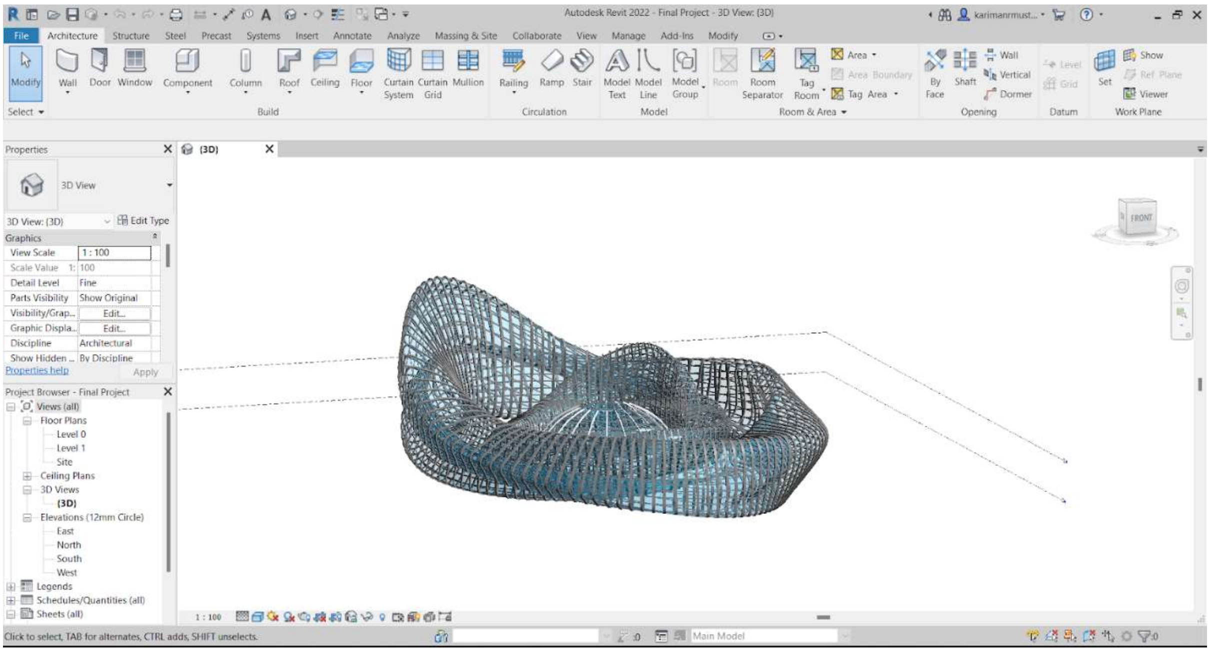

Figure 31. The final product on Revit

IV. Summary/Outline of the Interoperability process between Rhino & Revit

1. Rescaling Model
2. Exporting dome surfaces (ACIS.sat) and placing the mass, defining it as a “Curtain System.”
3. Exporting Mullions (Rectangular Mullions) and adjusting Mullion size to mass using Duplicate & Edit Type
4. Defining Horizontal Walls as Roof by Face (since Wall by Face and Mullions resulted in errors).
5. Inverted Cone as Curtain System with Circular Mullions (Radius 0.25m)
6. U and V Panels as Wall by Face
7. Interior Surfaces (Mobius Curves Relofted) as Curtain system (while adjusting Curtain Grids) to fit the shape
8. Floor Mass to add Floors on separate levels on Revit, then use the Floor by Face command.

V. Conclusion

In conclusion, interoperability requires multiple trials and errors to reach the desired results; frequently, to achieve the desired results, one must dedicate time and effort, including enough research, to gain enough experience with the interoperability process. It is certainly time-consuming; however, one could easily obtain excellent results with practice. Although we have only been through one assignment regarding interoperability, I learned over time with the Final Project that collaborating effectively with Rhino and Revit allows us to quickly implement and optimize our work. Moreover, as the project progresses, you realize that while some things may seem as though they are the easiest, they could be the most strenuous and demanding; however, it is no doubt that problem-solving gets you through anything!

VI. Lessons Learnt

1. It is a “Rhino to Revit and Back” Process

2. An elegant solution to any challenging problem is, as the phrase suggests, problem-solving
3. Make it simple for Revit to make your life easier
4. Sometimes remodelling things produces better results
5. Staying focused and consistent is the only thing that can keep you going
