# Supplementary material for: Preserving conceptual design integrity: strategies for enhancing interoperability in architectural digital design workflows
Source: Sci Rep. 2024 Dec 23;14:30595. doi: 10.1038/s41598-024-78640-8 (PMC11666589; doi:10.1038/s41598-024-78640-8)
Supplement: Supplementary file 2 — Supplementary Information 2. [file 41598_2024_78640_MOESM2_ESM.pdf]

Appendices

Appendix 1.

1.1. The project's design briefing: Phase One: 3D Form Composition

Mini project: A World Persona Pavilion

Before a designer can explore building details, he/she will need to explore shapes beyond the box. Frank Lloyd Wright always believed in creating shapes that broke from the box. Today architects such as Norman Foster, Frank Gehry, and others believe in breaking the box through the creation of non-Euclidian, non-box like shapes.

Briefing

You are invited to design a pavilion to commemorate a world character you are inspired with of your own choice. You are given free rein to choose the character's identity and domain of contribution to humanity. He/she could be a novelist, a poet, a musician, a sportsman, a scientist, or any sort of domain that you aim to shed lights on.

This pavilion is to be 3D modelled on Rhino and visualised on Lumion. You may think of rendering several shots from different angles of the created composition to deliver your concept to the viewer. Imagine the journey that a guest visiting this pavilion would pass through to get knowledge inspired by the commemorated character; *what would the guest of this persona pavilion be seeing?*

A tip to consider: The 3D composition of your Rhino model should express this character; it may inspire his/her profession, outlook, outfit, hairstyle, stages of life, ... etc. You may use your own freehand sketches, scribbles and/or photos from any resources according to your research in expressing your ideas and add them to your project board. It is quite important to show the steps of development of your 3D Rhino model on your project's board. This is called 'form generation', which shows the curves, surfaces, masses assigned to the operations and commands of rhino.

Create a poster of A1 size on PowerPoint or Photoshop. The poster design should be a background of the project, use light colours in your poster to direct the viewer's attention to the project photos not to the poster's decoration. Choose a title for the pavilion and write it in a clear bold font on top of the board. Add the Lumion shots of the form and the form generation (Rhino steps) on the poster in a meaningful creative way. The project's board size is A1, print the poster in colour, and stick it on an A1 foam board. This mini project is a milestone ahead to achieve; hence, we are approaching the completion of Stage (I) on 3D Form Composition.

Pavilion Size:

Volume: Design a space of 9 * 9 * 9 m^3

Requirements for submission:

- A printout of A1 portrait (poster) renders of the 3D pavilion composition
- A paragraph of 150 words maximum to express your chosen concept, freehand sketches (if any), images of inspiration all together to be included in the poster design.
- Upload a softcopy of your poster and the Rhino 3D model on Blackboard.

Skills refined in this assignment:

1. Using the skills and modelling commands you have learnt on Rhino.
2. Creating a Form composition with the tools you have learnt in Phase 1 of the course.

3. Creating a meaningful composition that the viewers of your project can perceive.
4. Writing a summary of your idea/rationale.
5. Using the rendering skills, you practised on Lumion.
6. Taking different camera shots on Lumion.
7. Using Photoshop or other software for Poster design and presenting your project.

Objectives:

This assignment aims at paving the way to practice and develop your modelling and creation skills in three-dimensional form compositions, which have a meaning as well as an architectural function using 3D modelling methods in Rhinoceros application. It also aims at advancing the basics of rendering using Lumion and presenting a post-production poster of your project.

Grading criteria and grades distribution:

- Concept 20%
- Rhino modelling of the 3D composition; including proficiency in using the tool, neatness, and precision 40%
- Form Generation Steps 10%
- Visualisation 20%
- Poster Design 10%

Rubric:

Rubric descriptors are included in the lecture slides with samples of projects and previous students work (a subject of discussion in the lecture)

- **D** → Unclear meaning to the concept that can be inferred through the proposed 3D composition and rendering is too weak.
- **C** → The transition of what is written about the concept and composition is unclear, with poor rendering.
- **B** → Very good expression of the concept through the three-dimensional configuration and showing good skills and deployment of the tools that are learned in Rhino application, as well as a good rendering on Lumion.
    - B^-→ Fair expression of the concept, fair rendering.
    - B → Good expression of the concept, good rendering.
    - B^+ → Very good expression of the concept, very good rendering.
- **A** → The idea is exceptional, new, and showing a wonderful expression through the three-dimensional composition with an excellent demonstration of the tools which you are trained on in Rhino application, as well as an excellent presentation and rendering of your project.
    - A^- → A powerful Idea, and very good form composition and rendering capabilities and aesthetics
    - A → A powerful idea, and excellent form composition and rendering capabilities and aesthetics
    - A^+ → A Powerful Idea and exceeding form composition and rendering capabilities and aesthetics

Appendix 2.

Final Project: In-depth detailing mini project 1

The final project is about in-depth detailing mini project 1; creating full package drawings of the Rhino model by applying the interoperability process in Revit aiming to extract the plan, section, elevation, and 3D perspective, and visualising these projections using Photoshop. By this, you will learn how to produce in-depth package drawings for a project. This project aims at strengthening your skills of a digital workflow that you will need in the subsequent design studios.

The Digital Process – Workflow

- **3D Model**: The 3d composition was already created in Rhino.
- **Interoperability**: Implement interoperability between the Rhino 3D model and Revit to extract the following drawings:
    - **Plan (1:50)**: you will get the plan created automatically in Revit after importing the Rhino 3D mass. The plan should encompass two functional spaces, which you will build in Revit inside the imported shape. Suggested functional spaces could be a specialised exhibition room and an office, or something you choose out of your experience and imagination, of 12 sqm each.
    - **Section (1:50)**: Cut through the final 3D form in Revit in a way that shows the elements of the building in detail.
    - **Elevation (1:50)**: Choose one of the sides that represents your concept.
    - **3D Perspective**: Choose an angle to save the perspective shot; it could be a bird's view or human's eye.
- **Visualisation**: Export the drawings (plan, section, elevation, and 3D perspective) to Photoshop to render implementing the techniques you have acquainted in the in-lab tasks 07 and 08.
- **Poster Design**: create a poster in Photoshop that combines the triadic projections and 3D perspective, and concept. The poster size A1, you can use two A1s as maximum posters.

Note that:

- **<u>All the previous requirements are graded</u>**.
- Be cautious to use the same <u>resolution factor</u> in Photoshop equal to the one you've used in Revit, so that the triadic projections preserve their ratios and scale (1:50).
- When you print the poster, your drawings are with correct dimensions and at scale 1:50. This is a major element in the grading criteria.
- Form Generation: The modelling steps in Rhino and Revit should be recorded, annotated and shown in the final poster(s).

<u>Submission Requirements</u>

- A paragraph of 50 words (maximum) to express the concept should be shown in the poster.
- Upload of the following files (softcopies) on <u>Google Drive</u>:
    - The Rhino and Revit 3D models.
    - The PDF drawings before and after visualisation

- The A1 poster(s) after Photoshop visualisation.
- Photoshop files to be submitted in PDF format.
- Include any freehand sketches if used in the concept development phase in the final poster(s).

Software Skills Required and objectives

1. Strengthening the three-dimensional modelling techniques, you have learnt on Rhino and Revit.
2. Mastering Interoperability feature between Rhino and Revit –allowing merging free-form modelling with BIM Platform.
3. Creating a meaningful, conceptual 3d composition that can be perceived and writing a summary of your idea (rationale).
4. Producing drawings from Revit at a certain scale and printing correct (precise) dimensions.
5. Mastering post-production (rendering techniques) on Photoshop.
6. Poster design to present your project with accurate drawings and panelling.

Distribution of Grades:

1. Rhino 3D composition 10%
2. Revit 3D composition 10%
3. Applying Interoperability 20%
4. Showing proficient use of digital tools (Rhino, Revit, Photoshop) 10%
5. Extraction of correct 2D drawings 10%
6. Visualisation of drawings on Photoshop 20%
7. Poster design 10%
8. Final Poster(s)' print (PDF) in correct dimensions 10%

Rubric Description:

A

- A novel idea: the expression through the 3d composition in Rhino and Revit is clear, transforming the message into the 3d composition is meaningful and clear.
- Showing a proficient use of the tools.
- Interoperability is achieved.
- Correct 2d drawings.
- Visualisation of drawings: fostering the concept through materials effect and shadows is outstanding.

A⁻

- A novel idea: the expression through the 3d composition in Rhino and Revit is clear, transforming the message into the 3d composition is meaningful.
- Showing a proficient use of the tools.
- Interoperability is achieved.
- Correct 2d drawings.
- Visualisation of drawings: fostering the concept through materials' effect and shadows is very good.

B+

- A novel idea: the expression through the 3d composition in Rhino and Revit is clear, transforming the message into the 3d composition is good.
- Showing a very good use of the tools.
- Interoperability is achieved.
- Correct 2d drawings.
- Visualisation of drawings: fostering the concept through materials' effect and shadows is good.

B

- A novel idea: the expression through the 3d composition in Rhino and Revit is clear, transforming the message into the 3d composition is less clear.
- Showing a very good use of the tools.
- Interoperability is achieved.
- Correct 2d drawings.
- Visualisation of drawings: fostering the concept through materials' effect and shadows is less clear.

B−

- A novel idea: the expression through the 3d composition in Rhino and Revit is clear, transforming the message into the 3d composition is unclear.
- Showing a very good use of the tools.
- Interoperability is achieved.
- Correct 2d drawings.
- Visualisation of drawings: fostering the concept through materials' effect and shadows is unclear.

C+

- A novel idea: the expression through the 3d composition in Rhino and Revit is clear, transforming the message into the 3d composition is un-clear and the idea is likely repeated.
- Showing a good use of the tools.
- Interoperability is achieved.
- Correct 2d drawings.
- Visualisation of drawings: fostering the concept through materials' effect and shadows is unclear.

C

- A good Idea: clarity in expressing the 3d composition in Rhino and Revit and delivering a message is unclear and the idea is likely repeated.
- Showing a good use of the tools.
- Interoperability is barely achieved.
- Correct 2d drawings.
- Visualisation of drawings: fostering the concept through materials' effect and shadows is unclear.

C−

- A good Idea: clarity in expressing the 3d composition in Rhino and Revit and delivering a message is unclear and the idea is likely repeated.
- Showing a good use of the tools.
- Interoperability is barely achieved.
- Correct 2d drawings.
- Visualisation of drawings: fostering the concept through materials' effect and shadows is unclear.

D+

- A vague Idea: clarity in expressing the 3d composition in Rhino and Revit is not achieved, there is no message.
- Not showing a good use of one of the tools.
- Interoperability is not achieved.
- In correct 2d drawings.
- Visualisation of drawings: presenting the concept through materials' effect and shadows is unclear.

D-

- A vague Idea: clarity in expressing the 3d composition in Rhino and Revit is not achieved, there is no message at all.
- Not showing a use of any tool.
- Interoperability is not achieved.
- In correct 2d drawings.
- Visualisation of drawings: not fostering the concept through materials' effect and shadows.

Good Luck,

273/1521 Tutors

Appendix 3. The commands used to convert a 3D algorithmic element in Rhinoceros into a native Revit element in Revit. Source: Authors.

	Rhino mass type	Revit native element conversion type	Both advantages and disadvantages
	**NURBS surface of curtain wall**	In-place-mass converted to curtain system	Works with simple surfaces
		Or: wall-by-face then modify material	No mullions come with the surface, needs to be created in Revit
		Insert as component → wall (not curtain wall)	Un editable, you cannot change the thickness or add mullions to it.
	**Mobius strip**	Importing all the surfaces at once to Revit	Problem: Revit was not responding to all the surfaces at once and Remedy: dividing the strip into sets instead of exporting them all at once and exporting each piece individually.
	**Mobius surface**	Loft with a lot of curves and a lot of control points	Error message from Revit (when exporting): The family is empty, and it will be deleted. Remedy: Working with simple surfaces with a smaller number of control points
	**Solid geometries**	Exploding the object to separate the surfaces (Ex: Revit does not understand the Box shape)	works and each face can be converted to (Wall, Floor, Roof, and Curtain system)
	**Sphere**	Boolean split or Boolean union between multiple complex geometries	Does not convert
		Split → wall-by-face	Convert but you cannot add mullions to it if is curtain wall
		Split → curtain system	Sometimes it does not convert. If it works, the challenge is to adjust the mullions alignment between the split parts.
		Insert as component → wall	Un editable, you cannot change the thickness to it only materials can be edited.
	**Sub-D → NURBS**	Convert Sub-D to NURBS then Explode it	Sometimes the project is too complicated and has a lot of surfaces after extracting.
		Insert as component → (wall, Floor, Roof, etc.)	Nonedible, you cannot change the thickness and cannot add mullions to it if you want to make it a curtain wall.
	**Mesh → NURBS**	Convert Sub-D to NURBS then Explode it	Sometimes the project is too complicated and has a lot of surfaces after extracting.
		Insert as component → (wall, Floor, Roof, etc.)	Nonedible, you cannot change the thickness and cannot add mullions to it if you want to make it a curtain wall.

	**Open Surface (Open Curve)**	Surface with pointed ends must be chamfered to two edges at least to be converted	Pointed ends cannot be converted to walls but they can be converted to curtain systems.<br>It works with a wall when it isn't with a vanishing point, or: split it to small parts.
	**Looped/Closed Surfaces (Curves with intersections); Lofted Surfaces with transformation commands such as cage edit, twist, Bend, …**	If inserted as (In-place-mass)	Cannot be converted to → (wall, Floor, Roof, etc.)<br>Remedy: Unless exploded or split to multiple faces
		Insert as component → wall, Roof, Floor, or mass (not curtain wall)	Nonedible, you cannot change the thickness or add mullions to it; only materials can be edited.
	**Looped/Closed Surfaces (Curves without intersections)**	If inserted as (In-place-mass)	It works when it isn't too complicated to be converted to → (wall, Floor, Roof, etc.)<br>If it is too complicated, it must be separated into small parts.

Appendix 4: Research Questionnaire on Interoperability

Name: ....

How do you estimate how far your core project idea has progressed from Phase 2 to Phase 3 after implementing the interoperability process?
Describe your rating on a scale of 1 to 5:
1, 2, 3, 4, 5

Has an element of the project you designed in Rhinoceros Circus been completely changed?
Yes, No

If the answer is yes, then explaining what the reasons are for changing the shape from what it was:
....
....

And did you redraw this item/s again in Rhinoceros?
Yes, No

Or did you redraw an element of the project in Revit?
Yes, No

If you want to redraw one or more elements in the Revit program, explain why? What are the reasons why I left Rhinoceros and preferred modelling this element in Revit?
....
....

Appendix 5: Classification of problems and solutions presented during the pilot study on Rhinoceros and Revit interoperability - students sample sourced from the ARCH 273/1521 coursework archive (Spring, 2022).

**Student name**<br>**Project title**	**Problem**	**Solution**	**Classification**
5.1<br>Nada Ahmed<br>(Musicverse)	All masses did not appear neither in 3D nor in the elevations.<br>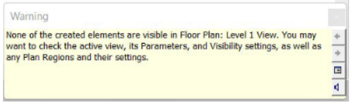	Changing visibility and graphics options from settings in order for it to appear.<br>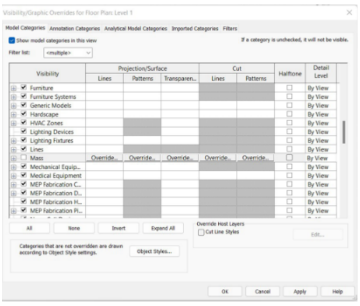	Revit Settings
	Levels were too small while importing the masses.	Rescale the building from the beginning and import it again.	Scale
	Could not import the whole project at once.	Divide each building into approximately 8 parts, ended up with 43 masses with the entrance and the bridges.	Solid
	The building A did not want to be identified as wall at once.	-Divide the building A into the body and pipes.<br>-Import the Revit file into another Revit file and explode it the easy way.<br>- Identify each part of the body by wall by face.<br>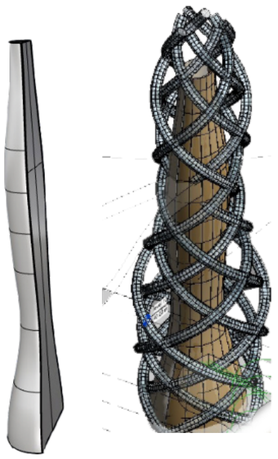	Solid

	The building B were divided into many parts that identified by walls, but it does not work.<br>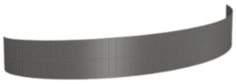<br>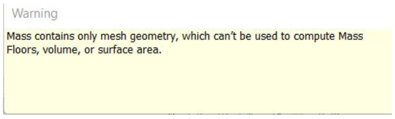<br><br>Trying to identify it as ''curtain wall'' but it also failed and this message appeared.<br>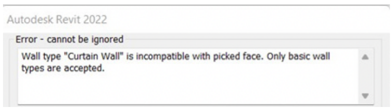 	-Trying to identify it as ''curtain system'' and it finally got identified.<br>-Exporting pipes surprisingly failed so it had to create mullions on Revit from Architecture-mullions by selecting on each line.<br>-It was time consuming but to show the building in its best way.<br>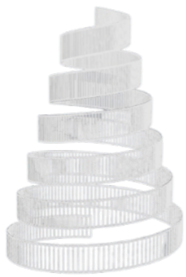	Curtain wall
	The building C after being divided into many parts were identified by wall by face.	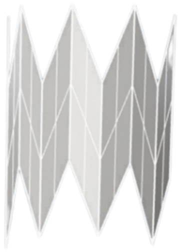	Surface
	The Bridges and entrance rejected it as a mass.	-It had to go back to Rhino<br>-extract the surface.<br>-change the bridges shape making it less curvy.<br>-decrease editing points so it can get identified on Revit by roof by face.<br>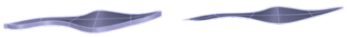	Surface
	The plaza had a curved opening in the middle, the student tried to identefy it on Rivet by wall by face, roof by face, and floor by face, but it faild.	It ended up learning a new tool to draw the design of floor on Revit including the curves (whole of the plaza)''using 'pick line' tool.<br>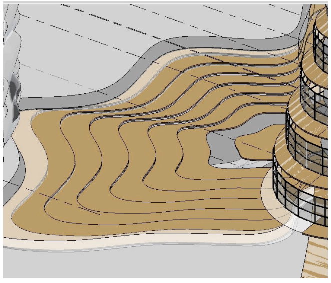	Surface

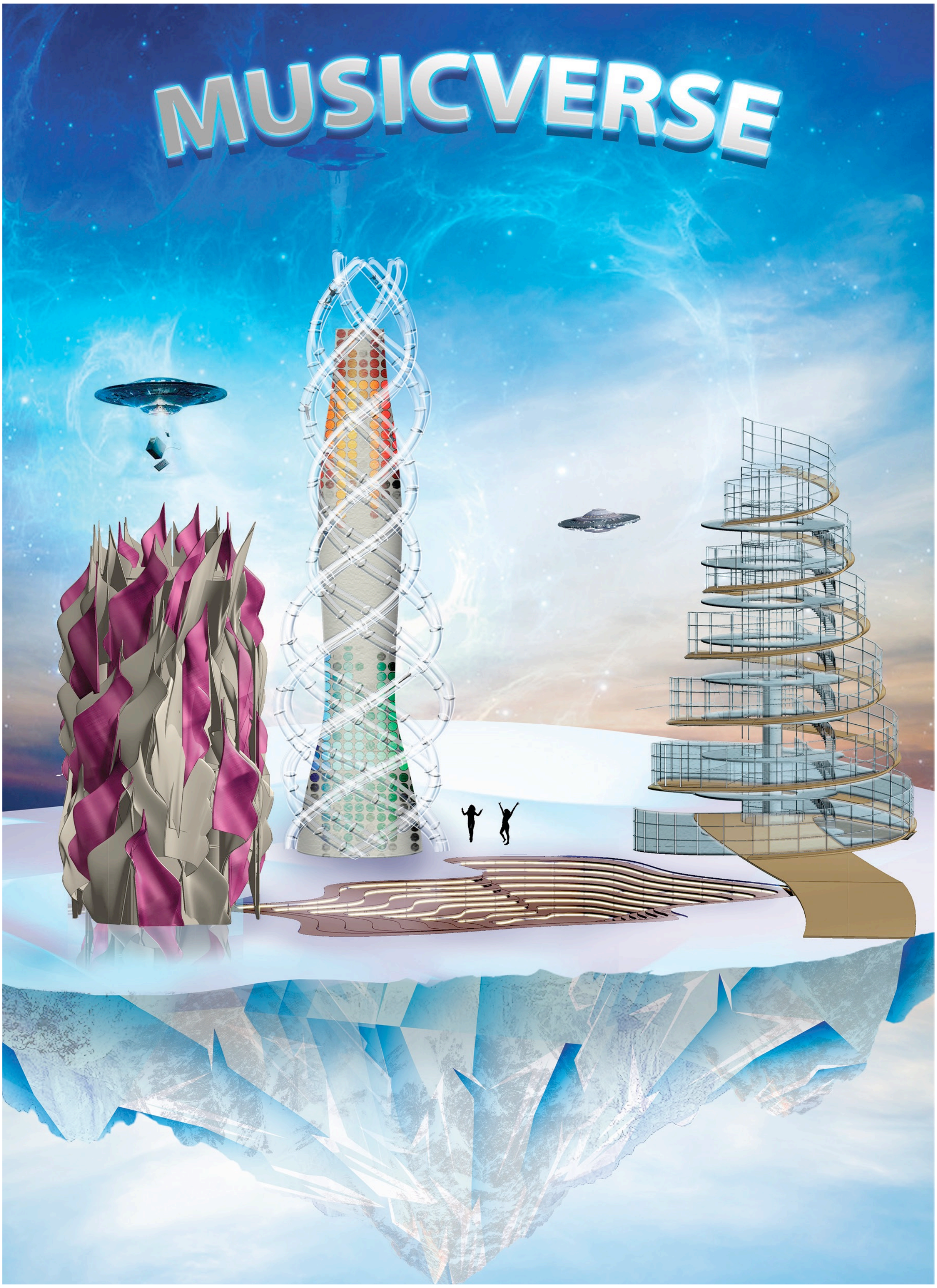
MUSICVERSE

<table>
<tr><th>Student name
Project title</th><th>Problem</th><th>Solution</th><th>Classification</th></tr>
<tr><td>5.2
Lugine Yosr
(Laputa)</td><td>The glass walls were a little too basic so the student wanted to add more details so she tried to change them to curtain walls but they would not change in either of the bases and would give her an error message.
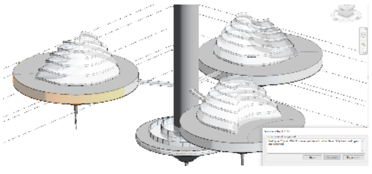
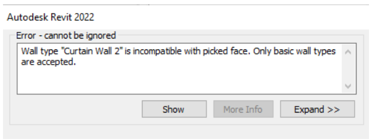
</td><td>After trying several times with the curtain wall and still getting error messages she decided to delete the glass walls instead of trying to change them to curtain walls and creating a new curtain wall and selecting the empty base area and defining it as a curtain wall. When she tried this method, it worked and she was able to make the base into a curtain wall.</td><td>Curtain wall</td></tr>
<tr><td></td><td>I wanted to add stairs that connect the 4th floor with the 5th, but when I got to the railings, I kept getting a warning message.
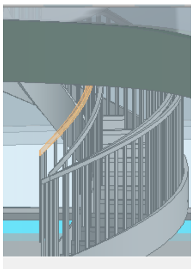
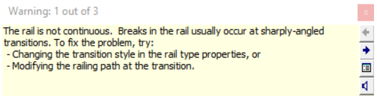
</td><td>After trying to solve the problem multiple times and still getting the same warning message. Finally, coincidently, the student found that she had accidently put two stairs over each other so they were overlapping and the railings were not connecting. Once she deleted the extra staircase the problem was solved.</td><td>Stair</td></tr>
</table>

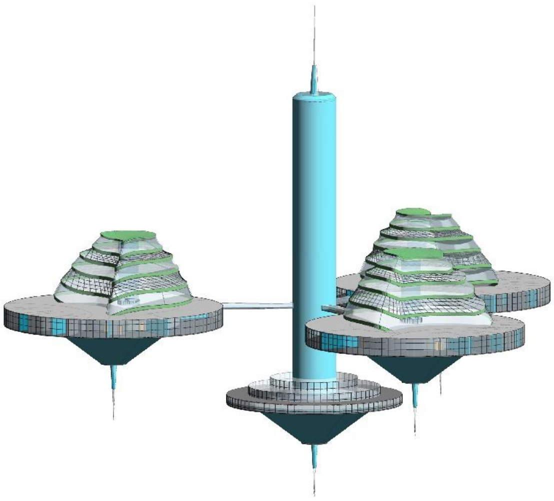

Student name<br>Project title	Problem	Solution	Classification
5.3<br>Kariman Mustafa<br>(Marsfprecious)	When the student inserted a mass from Rhino to identify it as curtain walls, she had to create a curtain system. Revit has warned her that the panels in the curtain system might be malformed.<br>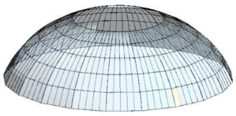<br>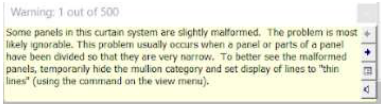 	The student determined that she had to decrease the spacing between the curtain wall grids to obtain a smoother surface, this adjusted the curtain grids on the dome.<br>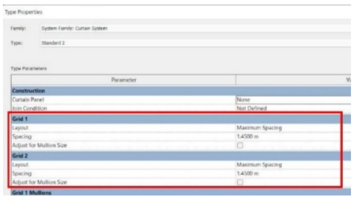	Curtain wall
	The student created mullions that fit the same structure and shape. She tried changing the width on both sides in the dimensions panel, but it seemed as though it would only change the dimension of the mullions from one side.<br>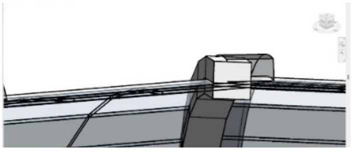	The student thought of only changing it from one side and then duplicating it to the other side to obtain the correct mullion size.<br>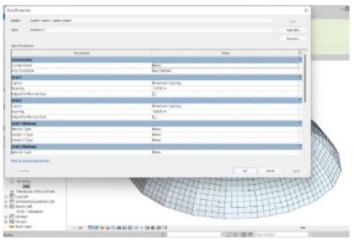	Curtain wall
	As the student was trying to identify the horizontal walls as mullions, they affected the vertical mullions due to the connection.	Identify the mass to Roof by Face.<br>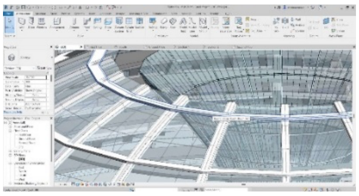	Roof
	The student tried to export the horizontal shells for the smaller Mobius surface. However, Revit was not responding to all the surfaces at once.<br>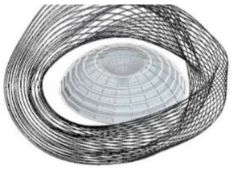	Exported the surfaces from Rhino by dividing them into sets instead of exporting them all at once, then identify them in Rivet using Wall by Face.<br>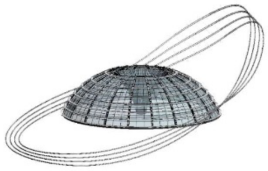	Surface

	The last step in the interoperability process of extracting from Rhino to Revit was the surfaces between the U and V panels. The student was receiving this message.<br>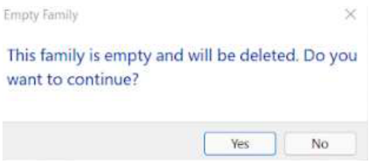 	Re-loft the surface on Rhino to reduce the complexity of the surface, and import it again inside Revit.<br>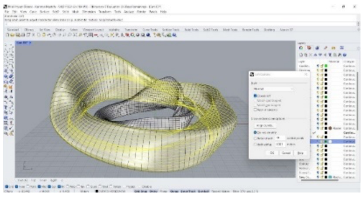	Surface
	While creating the curtain walls, the student requested Revit to create a Curtain System; however, the spacing between the curtain grids was incorrect, leading to random glass panels.<br>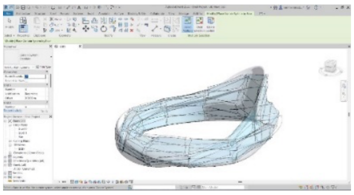	Decrease the spacing between the curtain wall grids to obtain a smoother surface.<br>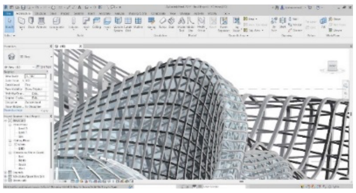	Curtain wall

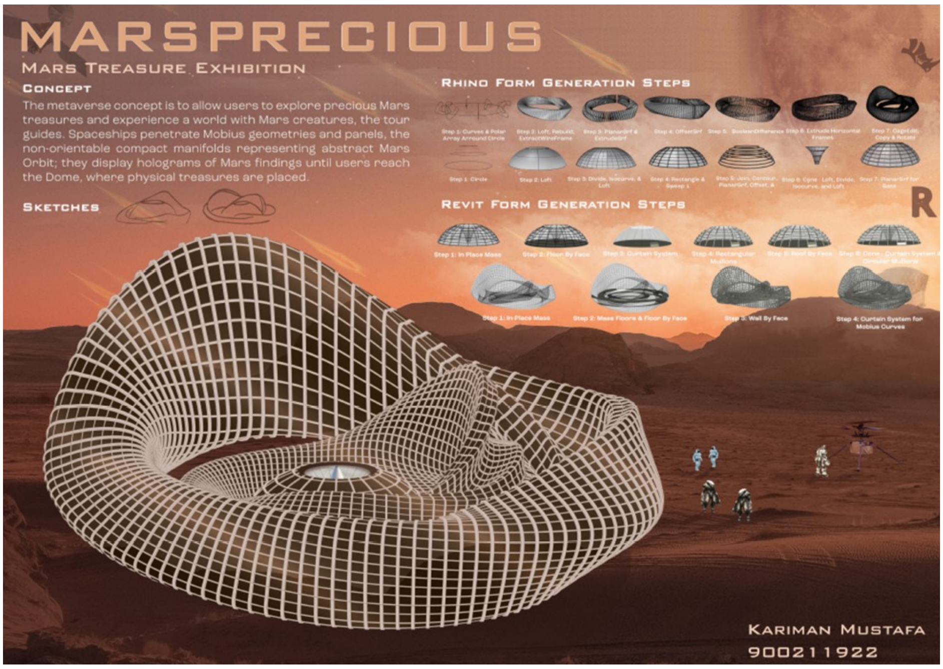

Student name<br>Project title	Problem	Solution	Classification
5.4<br>Rana Zahran<br>(Robotics Exhibition)	In the mini-project phase, the student created a model using SUB-D forms in Rhino, which created several obstacles for her in exporting the SUB-D forms from Rhino to Revit.<br>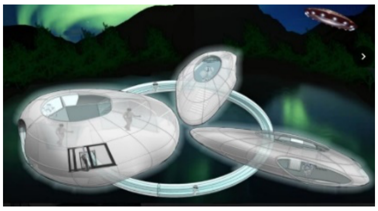	Recreate the model again on Rhino using curves and surfaces.	SUB-D
	The student drew the tunnel again using curves on Revit, and identify it using wall-by-face. As for the spheres, which were very exhausting and time-consuming.	Create them by drawing curves and patching them on Rhino, then exporting them again to Revit.<br>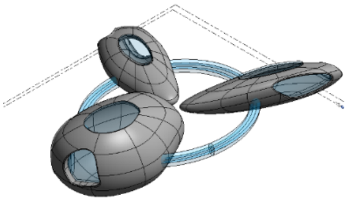	Surface
	Recreating and selecting individual curves either in Rhino or Revit while changing the elements' places was extremely hard,	The student separated each one on a layer in Rhino, and then hide the elements she did not need.	Layers
	The student realized that the scale was extremely large when she placed the views on the A1 sheet on Revit.	Scale the project down on Rhino then import everything again to Revit..	Scale
	While trying to create the interior glass walls of the display. Because the model is sphere-like, the normal wall command would get beyond the exterior walls of the model.<br>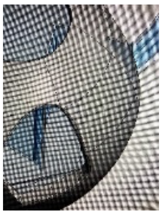 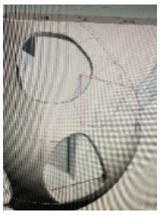	The student adjusted each wall separately using the edit profile command after selecting the wall, she had to view the model from several perspectives and adjust an incorrect alignments.	Wall

	Similar to the previous problem, because of the circular structure of the model. The student had to make the floors in the level where the diameter is the biggest. However, she still faced a problem drawing the floor by most floor commands including pick lines	The student used the edit profile on Revit after selecting the closest curves and adjust the floor to the shape of the floor she desire. 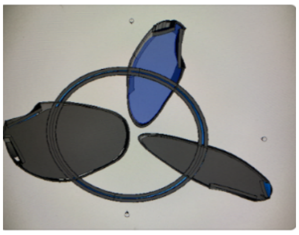	Floor
	When creating the entrance for the models, the doors were not aligned with the model. 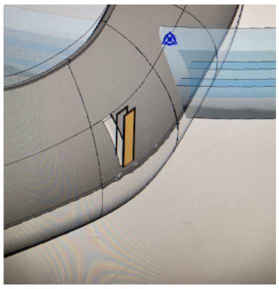	Create an alternate option by doing a wall by face with different material and an opening within it. 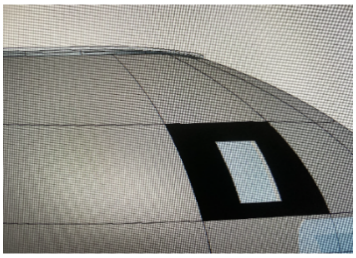	Opening

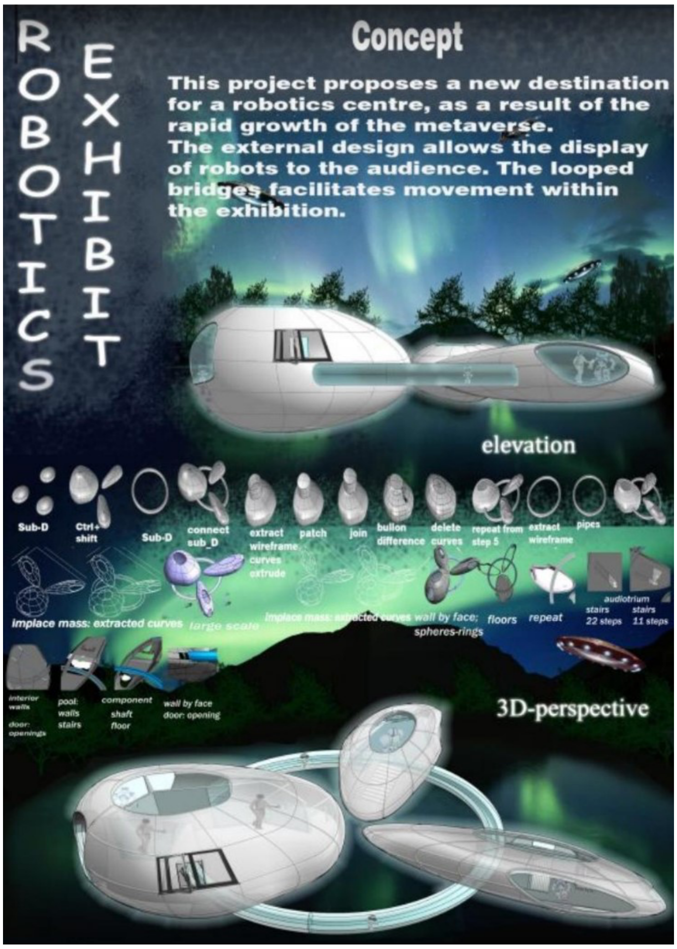

Student name<br>Project title	Problem	Solution	Classification
5.5<br>Abdelqader Alaswad<br>(NFT Hub)	Sphere wireframe and inner frame: -<br>There was no problem with both wireframes where both of them worked with wall by face but the student eventually had a problem with the size of the Revit file where it exceeded two gigabytes, which made the program very slow.	Import it by using Architecture > components> model in place> wall >insert >import CAD. This has reduced the file to a significant amount.<br>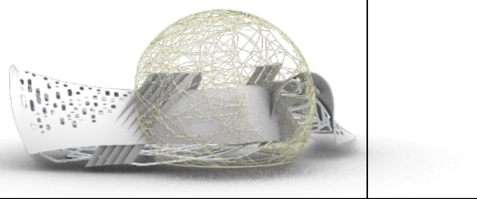	Size
	Since the mesh surface was a SUB-D surface, the student couldn't just use the rebuild command to lower the number of control points.	The student converted objects to NURBS tool and then the explode command to divide the mesh into different parts.<br>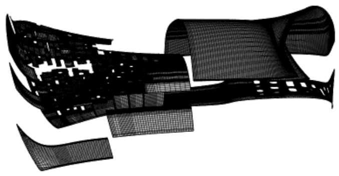	Mesh
	Even after changing the objects from mesh to NURBS, Some parts of the roof were impossible to be selected by roof by face in Revit due to the complex forms.	The student imported the objects by Architecture > components> model in place> roof> insert> import CAD.<br>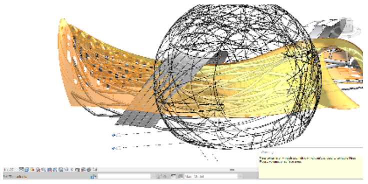	Roof
	When making Revit elements like floors and railings, some lines need to be refined to be able to use the pick line tool to create accurate elements.	The student used AutoCad to Refine these lines. To refine them he used the PEDIT Command to edit the precision points. Then he imported them back into Revit.	Curves

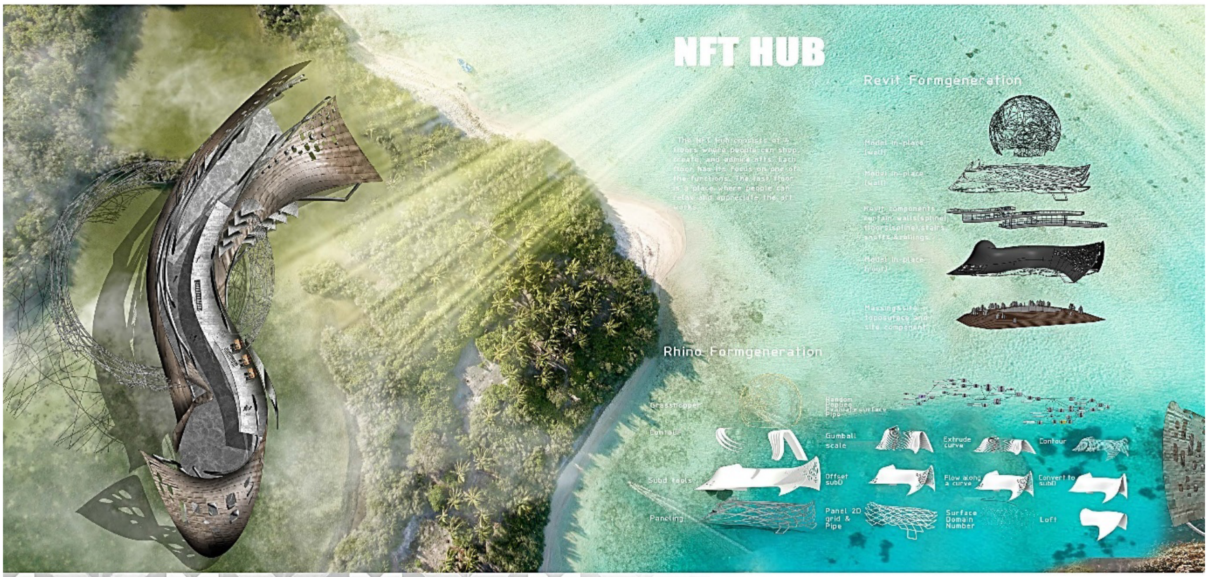
NFT HUB
Revit Formgeneration
The NFT Hub consists of 4 floors where people can shop, create, and admire nfts. Each floor has its focus on one of the functions. The last floor is a place where people can relax and appreciate the art works.
Model in-place (wall)
Model in-place (wall)
Revit components contain walls(spline), floors(spline),stairs, shafts &railings.
Model in-place (roof)
Massing&site - toposuface and site component
Rhino Formgeneration
Grasshopper
Random
Pipe
Contour
Gumball scale
Extrude curve
Contour
Subd tools
Offset subD
Flow along a curve
Convert to subD
Paneling
Panel 2D grid & Pipe
Surface Domain Number
Loft

Student name<br>Project title	Problem	Solution	Classification
5.6<br>Haya Ragaey<br>(Metaverse Portal)	The student imported the complete Rhino model into Revit as a single *.sat file. So, she attempted to create the wall by face, but she ran into an issue with the roof, which prevented her from building a wall by face.	The student opted to enter the roof as a single .sat file, which didn't solve the problem, so she made it into a curtain system instead of a wall, which seemed to work.<br>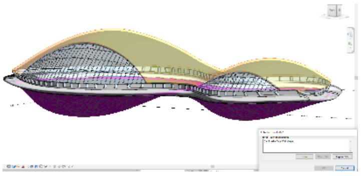	Curtain system
	The student attempted to make the pipes into glass walls, however it appeared that some parts of the pipes could pick up on the commands while others could not.<br>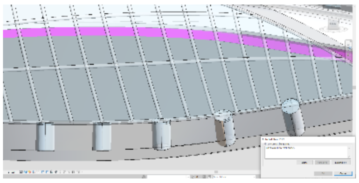	Transforming the window, pipes, base, roof, middle portion, and floor separately in new .sat files, making either a wall by face or a curtain system.	Import
	Facing into the exact issue with the window pipes once more.<br>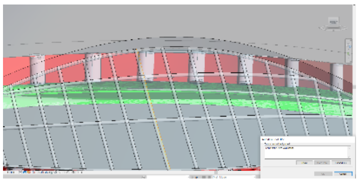	The student imported the .sat files in such a way that she could easily create a wall by face by clicking on components, placing in mass, then choosing what she want to identify the mass as in this case wall, then placing the cad file individually and repeating the process with each individual .sat file. When it came to transforming the mass into a wall.	Import
	The student discovered that a part would appear even though it was identified as a wall by face, but this part would disappear each time she closed Revit and opened it again.	Import it again and place it on Revit with a separate .sat file in order for Revit to identify it as a wall to remain after closing the Revit file.<br>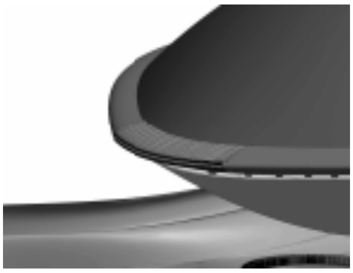	Wall

	The walls were extending from the roof as the model is curved. 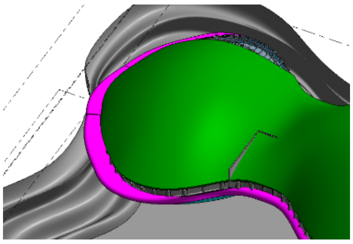	The student tried to attach the wall to the level but it didn't work, so she tried top and base offset in the hopes of making it work, but it didn't. So she decided to create a section in the middle of each wall, then re-edit the walls by deleting the bit that was peeking through the model.	Wall

Student name<br>Project title	Problem	Solution	Classification
5.7<br>Mariam Awad<br>(MET-FT Museum)	When the student exported the components from Rhino to put them in Revit, none of them were registered as anything, neither wall, roof, or floor.	The student adjusted the scale of the model to be smaller, since the radius of the dome was 150 m,	Scale
	In-place masses only was the only option work to identify the model inside Rivet because the entire model was mostly made out of meshes and thin surfaces.<br>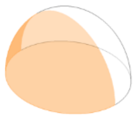	Rebuilt to decrease the meshes, and extruded the surfaces of the dome and spiral to be more volumetric for them to be placed in Revit.<br>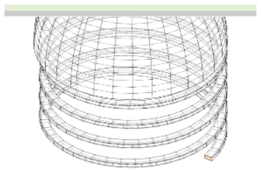	Mesh
	The model was composed entirely of curves and the walls are square like, so they'd be coming out of the dome itself, and the trim function did not solve the issue.	It's solved by using circular-empties tube like walls instead of traditional walls<br>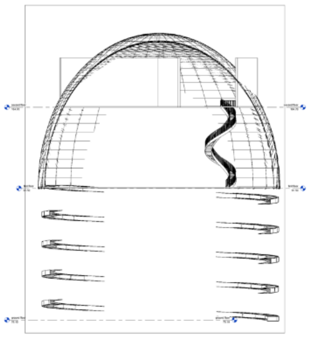	Wall

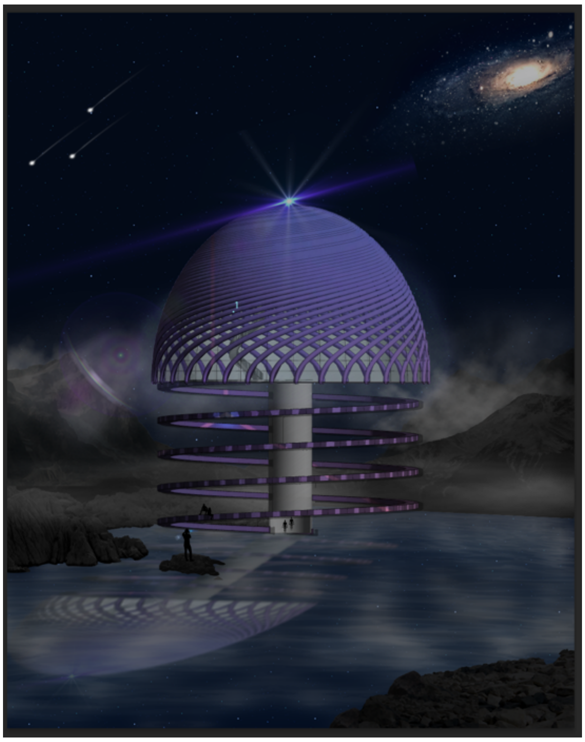

**Student name**<br>**Project title**	**Problem**	**Solution**	**Classification**
5.8<br>Maya Saad<br>(Metamorphosis)	Once The student entered the project elements on Revit. She noticed Revit was not accepting it due to its sheer size.	The student had to go back to Rhino and scale it to an appropriate size which came to around 55 meters, and import it again in Revit.	Scale
	The project was a little too intricate and organic. Revit struggled to read the exports as it had many interlocking curves and organic features.	Go back to rhino and take the brain shape that the student had done and extract its wireframe, erase all unnecessary and complex details and lofting it once again, then import them to Revit as a curtain wall.<br>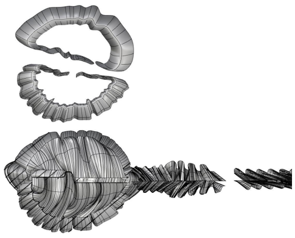	Surface
	Massing the brain shape as curtainwall did not give the desired smooth effect. It was chunky and very square.	The student had to go back to the settings of curtain wall material and play around with its mullion quantity and size until received the desired effect which was almost a mullion every 20 cm.<br>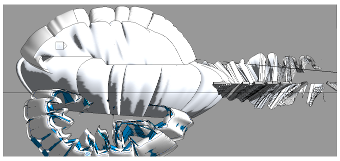	Curtain wall
	Massing the pavilion Spine structure was fairly easy. But the student massed it as a very thin wall but there were some exceptions where no matter who within a wall, it was refusing to mass.	Use curtain wall material to fill in the gaps and it actually gave the student a dynamic effect that would further help the design.<br>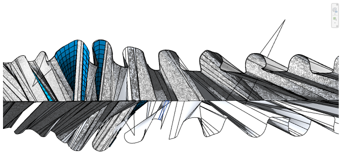	Curtain wall

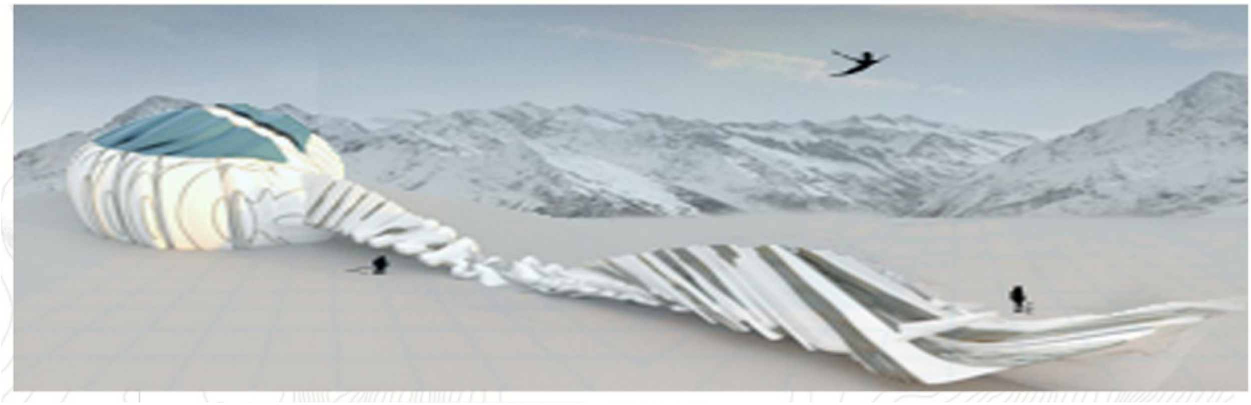

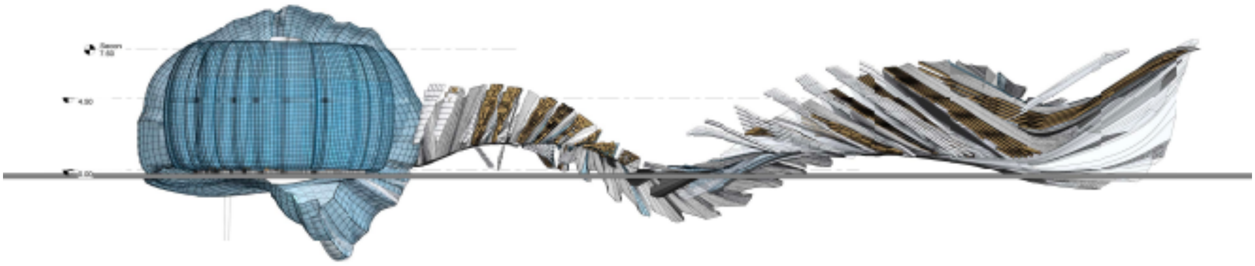

Student Name<br>Project title	Problem	Solution	Classification
5.9<br>Malak Ezz<br>(The CHROME-VERSE)	The project was unreadable in Revit, preventing the student from creating walls, floors, or roofs. To accommodate changes in the model, she started by constructing various mullions for each curtain wall, which was not a good idea.	The student started to fix the problem by trying to use the Explode command; however, it did not work, so she decided to split objects into 4 pieces.<br>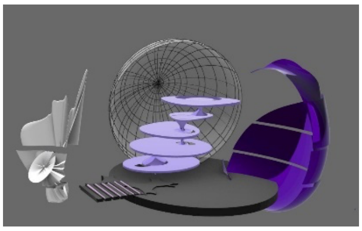	Solid
	The split option worked well for some objects on revit, but not for others, such as the white model, where 2 pieces were read as curtain walls, as a wall, and the other was not identified by revit as a wall. The student also noticed that this method made her model look less neat on Revit.<br>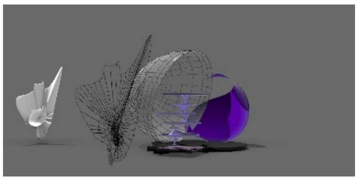	Using Extract WireFrame on Rhino, after eliminating certain lines and leaving the exterior and outer ones in the object, the student began using the CrvNetwork and Join commands to join these outer lines and form objects nicely, then she tried the Loft command, which did not work for all objects, so she used the Patch command to create the surface.	Surface
	The levels in Revit aren't accurate. Furthermore, the student was unable to make walls from the ground floor plan since it was not reading the external lines for it.<br>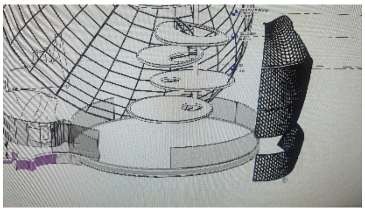	The student attempted to use methods by choosing line, which did not appear to read objects, and circle, which was less accurate. So she was able to create numerous arcs and glass walls using the radius arc option.	Curve

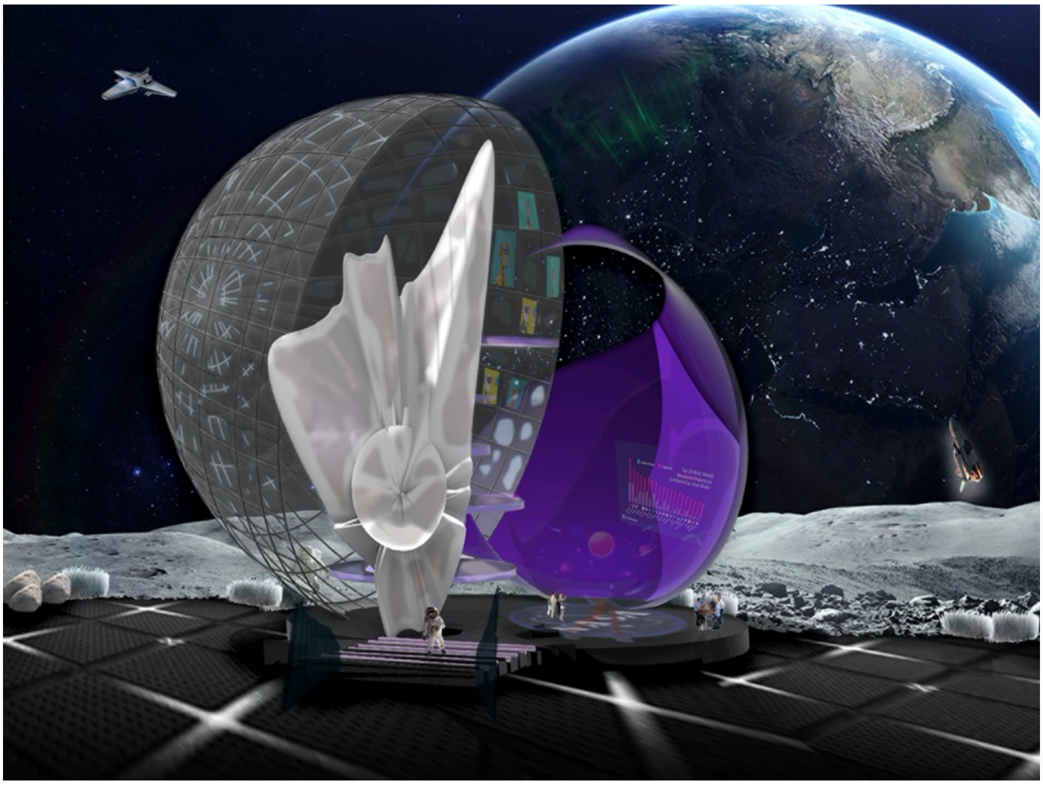

Student name<br>Project title	Problem	Solution	Classification
5.10<br>Rana El Shaer<br>(metaverse)	The student was exporting the full model from Rhino to Revit but it would keep exporting as one mass. When this happened, the student could not section out the levels and the model was too bulky.	Export the glass portion only and add curtain walls instead of exporting the piping in the model.<br>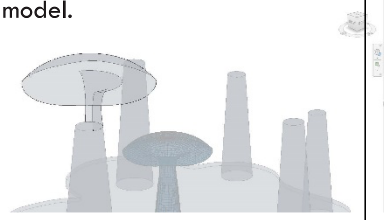	Surface
	A part of the mushroom top would not export properly from Rhino to Revit	Exploded the entire mushroom on Rhino so that the shaft was separated from the mushroom.<br>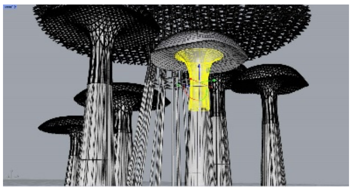	Surface
	When the student exported the biggest mushroom because unlike the rest of the model, it was made fully out of pipes. When it would get exported, it would turn into lines.	Placing it as a component (Wall) and not a mass on Revit and the shaft would also be placed as a component (Columns).	Wall

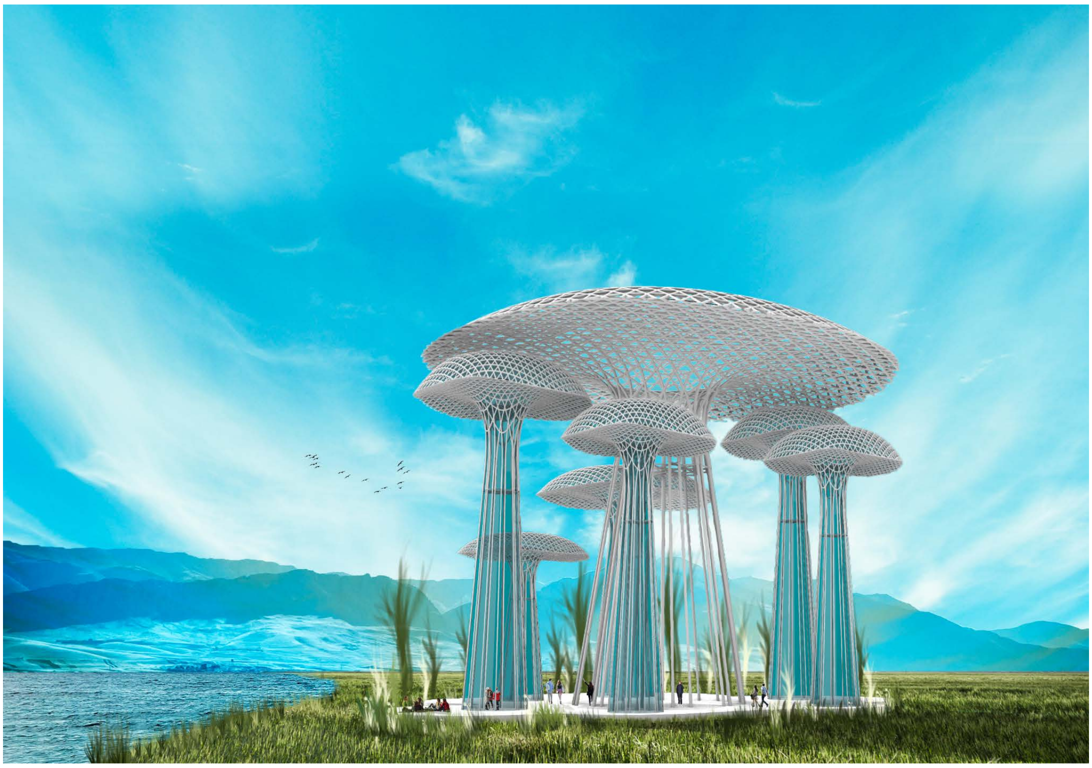

Student Name<br>Project Name	Problem	Solution	Classification
5.11<br>Hana Ali<br>(Love Buzz)	Whenever the student tried to convert and open the items on Revit, it will either become very slow or not even see the items on Revit.	Rescale the model down to a large extent.<br>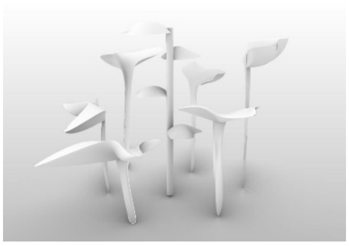	Scale
	The corridors' walls were hard to make at first because the student wanted them curved, and most options either gave her a straight line, an ellipse, or a wide arc.	Using Start-End-Radius Arc on Revit.<br>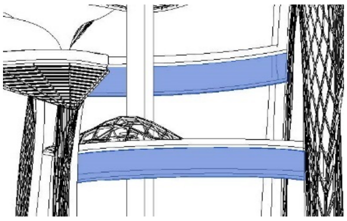	Surface

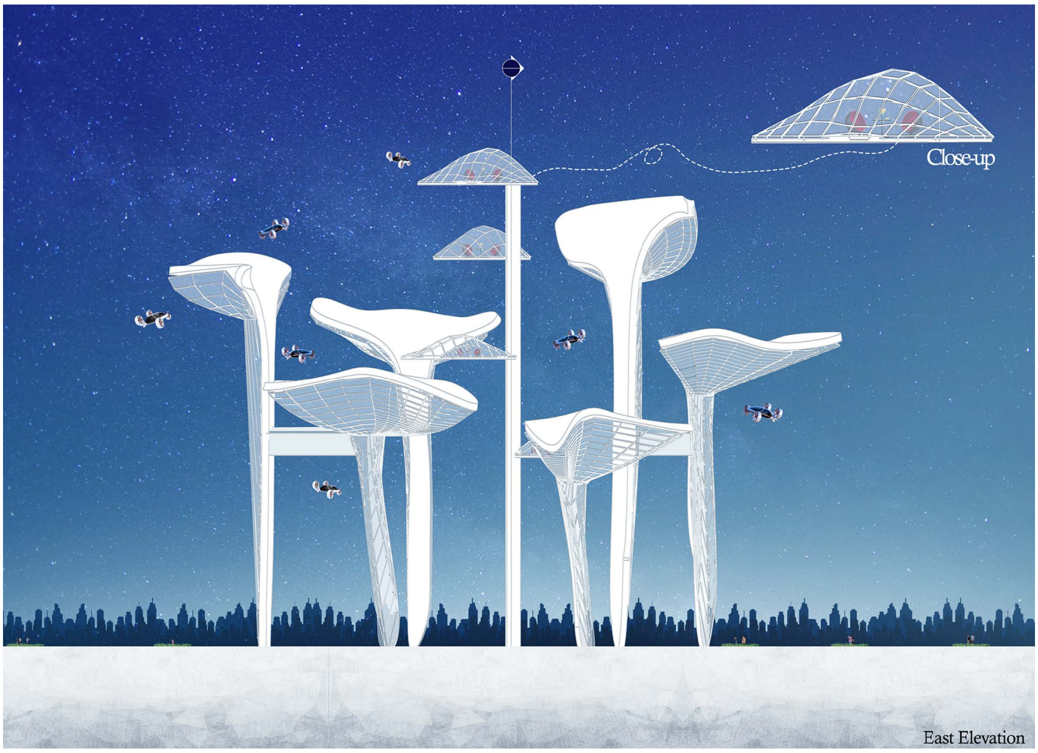

Student name<br>Project title	Problem	Solution	Classification
5.12<br>Omar Abdelzaher<br>(Love Buzz)	When the student tried to export the model for the first time from Rhino and import it to Revit it was very complicated for Revit to understand.<br>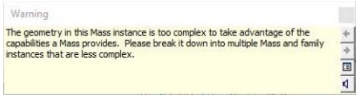	Use to NURBS command that converted the model from SUB-D object to NURBS object and then the student used explode command to less complicate the model, then it was exported successfully to Revit.<br>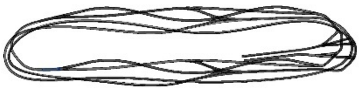	SUB-D
	It was struggling at the beginning to add wall by face since the model is all about glass tubes.	Imported glass texture into Revit and then the student added it to the wall by face category to build the tubes in Revit	Glass wall

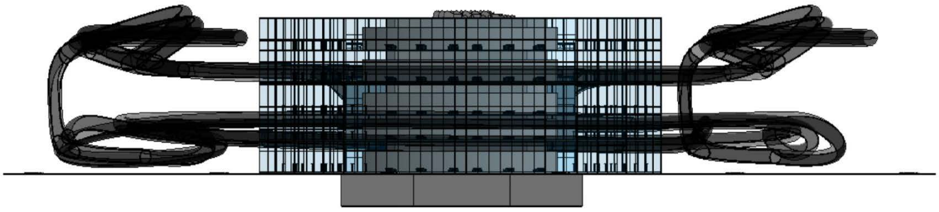

Appendix 6

An example of a report that each student was asked to prepare after completing their project and the interoperability process to highlight the difficulties encountered during the work and the solutions used to overcome them. The ethnographic observations made by the research team while supervising student projects were the basis for our investigation in addition to this report.
